# Brain MRI signatures across sex and CSF Alzheimer’s disease biomarkers

**DOI:** 10.1093/braincomms/fcaf210

**Published:** 2025-05-30

**Authors:** You Cheng, Yingnan He, Karthik Gopinath, Benjamin Billot, Juan Eugenio Iglesias, Chao-Yi Wu, Hiroko Dodge, Anne-Marie Wills, Becky Carlyle, Pia Kivisäkk, Bradley T Hyman, Steven E Arnold, Sudeshna Das, for the Alzheimer’s Disease Neuroimaging Initiative and for the European Prevention of Alzheimer’s Disease (EPAD) Consortium, Frank Tennigkeit, Andrea Pfeifer, Christine Vincenzetti, Fabien Monin, Gautam Maitra, Joseph O'Sullivan, Alex Teligladas, Ana Diaz, Christophe Bintener, Cindy Birck, Dianne Gove, Jean Georges, Katherine Ellis, Stefanie Peulen, Eva Danasi, Georgios Vlachos, Nicolaos Scarmeas, Alexandre Mencik, Cynthia Ng, Hernan J Picard, Mats Ericson, Matt Gaunt, Nicolas Pourbaix, Rob Lenz, Rodolphe Eyraud, Ross McGarrity, Veronique Chauvet, Ala-Eddine Allouche, Béatrice Le Balanger, Bruno Dubois, Edith Benmansour, Francis Nyasse, Hicham Filali, Ihsen Youssef, Leslie Ameil, Marion Dubois, Nabila Lajaail, Nabila Nyasse, Nadja Younsi, Patrick Feukam Talla, Souza Benadjaoud, Stephane Lehericy, Tatiana Akake Beshelemu, Belén Garcia Alonso, Bet Molina, Ian Sherriff, Pedro Pesini, Pedro Tirado, Virgina Pérez, Alan McNeill, Alicia Gibson, Andrew Fox, Bert Overduin, Christine Lee, David Allan, David Taylor, Emma McDonald, Harry Peaker, Karen O'Hanlon, Karen Thornton, Lorna Harper, Pamela Brankin, Robert Bryce, Rodrigo Barnes, Ana Belén Callado, Andreea Radoi, Annabella Beteta, Arcadi Navarro, Beatriz Blasco, Carolina Herrero, Carolina Minguillon, Eider Arenaza, Eva Nebot, Gema Huesa, Gemma Salvadó Blasco, Gloria Oliver, Gonzalo Sánchez Benavides, Gregory Operto, Iva Knezevic, Jordi Camí, Jose Luis Molinuevo, Juan Domingo Gispert, Julieta Pereda, Karine Fauria, Laia Tenas, Mahnaz Shekari, Maria Teresa Buongiorno, Maria Escrivà, Marta Milà, Meritxell Santos, Monica Montserrat, Montse Alegret, Neus Cadevall, Noemí Carranza, Oriol Grau Rivera, Paula Marne, Paula Petrone, Raffaele Cacciaglia, Sofia Menezes-Cabral, Barbara Wendelberger, Mark Fitzgerald, Melanie Quintana, Scott Berry, Shawn Andersson, Tammy Berry, Andy Bolan, Bjorn Sperling, Dominic Walsh, Elena Ratti, Emily Smyth, Fred Lawson, Inna Tsvang, Jeffrey Sevigny, Joao Pinho brandao, John Ogorman, Marcus Unternärer, Marija V Jankovich, Martin Pan, Olga Mitelman, Philip Liston-Kraft, Philipp von Rosenstiel, Samantha Buddhaeberlein, Sean Knox, Tina Olsson, Birgit Goerke, Catherine Debove, Daniela Harder, Dorothee Schwall-rudolph, Gine Plaue, Glen Wunderlich, Jasmin Ghazvini, Julia Meyer-Kleinmann, Kathrin Huber, Lisa Hattemer, Mark Gordon, Martina Eckes, Mary Potter, Melanie Nusser, Miguel Garcia, Pamela Drake, Pauric Mcginty, Rolf Goeggel, Russel Jones, Sabine Rau, Shannon Scanlon, Stephane Pollentier, Stephanie Sommer, Susanna Flaherty, Wilhelm Dieminger, Daniela Savina, Bernard Hanseeuw, Murielle Bortolotti, Jean-Francois Demonet, A A A Warszawa, Marta Biel-Czarnecka, Maciej Czarnecki, Enise I Incesoy, Felix Menne, Katja Lindner, Oliver Peters, Melanie Leroy, Florence Pasquier, Manon Laforce, Audrey Gabelle, Caroline Grasselli, Charlotte Leger, Aurelie Gillet, Christelle Cadiet, Claire Boutoleau, Manon Guiguen, Rachel Chaigneau, Romain Muraz, Samia Melcion, Tiphaine Charriau, Béatrice Dinthilac, Brune Rieunier, Bruno Vellas, Camille Coulange, Delphine Pennetier, Isabelle Carrie, Julien Delrieu Lapeyre, Laure Saint-Aubert, Natalia del Campo, Nicole Batonneau, Pierre Jean Ousset, Sandrine S A Andrieu, Sophie Mourgues, Genoveva Montoya, Mario Riverol, Virginia Blasco, Amy Gerrish, Elisa Majouni, Felicity Sheppard, Georgina Menzies, Harriet Oldham, Julie Williams, Keith Sexton, Rebecca Sims, Sian Jones, Steven Head, Teresa Bowen, Thomas Cushion, Valentina Scott-Price, Gael Chetelat, Ami Saver, Bruce Albala, Danielle Fry, Eve Potter, Johan Luthman, Jude Concepcion, Lan Bandara, Lynn Kramer, Martin Rabe, Michele Valeri, Nadeem Sarwar, Robert Gordon, Sharon Dispoto, Shilpa Kakad, Shobha Dhadda, Sindhuja Musku, Sonjoy Braham, Sue Lyons, Susan DevenishMeares, Alette Wessels, Andrew G Smith, Birgit Steckel-hamann, Chrissie Spears, Dan Holding, Eric Siemers, Florence Bayard, Holger Zimmermann, Jayetta Embry, Jennie Lenora Walgren, Jennifer Zimmer, Michael Hutton, Michael C Irizarry, Trevor Smart, Alexander de Jong, Cornelia van Duijn, Eline Bunnik, Johan van der Lei, Krista Tromp, Laurence Hoezen, Maartje Schermer, Ramona van Dijk, Sander Woerdeman, Fabian Perpeet, Hannah Wolff, Marc Zimmermann, Martin Hofmann-Apitius, Meemansa Sood, Tobias Rechmann, Ana Pancho, Charo Cuevas, Mar Buendia, Merce Boada, Amaya García-Eizaga, Mikel Tainta, Pablo Martinez-Lage, Jon Saldias, Marta Canada, Ben Newton, Christopher Foley, Anita Davies, Heena Mistry, Iracema Leroi, Rachel Rosenhead, Ross Dune, Rowen Norton, Tobias Langheinrich, Adrià Tort, Beatriz Bosch, Guadalupe Fernandez, Mircea Balasa, Andrea Fernandez, Jesus Pascual Sanchez, Sara Lopez, Marta Ferrando, Rocío García, Aixa Ghastin, Rafael Arroyo, Alicia Muñoz, Angeles Barro, Féix Viñuela Fernández, Tom Ashby, Tom Haber, Michael Ropacki, Alessandra Cincotti, Aurélie Gauthier, Aurélie Nier, Charlotte Dumonte, Florent Trecanne, Isabelle Carriere, Isabelle Geahel, Karen Ritchie, Marie Baquet, Marion Mortamais, Vincent Boyer, Andrew Satlin, Alasdair MacDonald, Andrea Morton-Morys, Andrew Danson, Anouska Penny, Ben Cons, Benjamin Spence, Betsy Cooke, Catherine Brooker, Cathy Vanbelle, Derenda West, Elisabeth Gilmour, Emma Parkinson, Fiona Ramage, Gary Johnson, James Rattenbury, Janet Hall, Jennifer Chambers, John Tracey, Joseph Milne, Lynne Hughes, Martin Dvorak, Rebecca Nagle, Salwa Beydoun, Shari Scott, Siva Kumar, Zia Haque, Alberto Redolfi, Anna Mega, Daniele Altomare, Elena Rolandi, Michela Rampini, Moira Marizzoni, Sara Gipponi, Valentina Nicolosi, Valentina Saletti, Samanta Galluzzi, Conor Wilson, Derek Hill, Elin Rees, Heather Welch, Ioana Ababei, Jane Whitrow, Janet Munro, John Hall, John Woodside, Kate Barnes, Kate McLeish, Lea Marai, Maartje Meijer, Mark Austin, Maryam Abaei, Mauro Sousa, Michelle Lax, Mike Radford, Oleg Belov, Rebeca James, Robin Wolz, Susan Lowther, Abbi Gower, Aline Smits, Ann Dierckx, Bart Vannieuwenhuyse, Caroline Sage, Christine Cully, Christophe Verbruggen, David Symnoski, Derya Ayaz, Enchi Liu, Eric Crouthamel, Gary Romano, Gerald Novak, Hendrik Sipma, Howard Greenberg, Isabelle Coste, Jeroen Schuermans, Jerry Novak, John Kemp, Joris Beke, Karen Bachner, Katrin Haeverans, Kyle Wathen, Laura Carrera, Lennert Steukers, Liesbeth Cluckers, Liesbeth Wouters, Lisa Ford, Luc Truyen, Lynn Yieh, Magda Van Dyck, Mahesh Samtani, Marie de Vulder, Marija Jovanovic, Marisa Ehinger, Mark Schmidt, Melanie Warzeski, Michael Fogle, Michele Mangini, Mila Etropolski, Nikolay Manyakov, Serge Van Der Geyten, Seth Ness, Simon Lovestone, Sipma Hendrik, Susan Katz, Suzanne Foy, Theresa Martino, Tim Elliott, Timothy Tracy, Vanessa Bosmans, Vladimir Dragalin, Wouter Deneyer, Yordan Godinov, Alina Solomon, Ann-Marie Stromberg, Applee Akter, Björn Kull, Francesca Mangialasche, Göran Hagman, Gunhild Waldemar, Gunilla Johansson, Hilkka Soininen, Ida Kettley, Katarina Risbecker, Malin Aspö, Marie Larksater, Myroslava Protsiv, Nenad Bogdanovic, Phatcharee Chareonto, Shireen Sindi, Steen Hasselbalch, Stefan Borg, Ulrika Akenine, Vesna Jelic, Miia Kivipelto, Jessica Röber, Robert Perneczky, Bjørn Grønning, Corine Baayen, Ejner Knud Moltzen, Helle Birgitte Mengel, Henrik Bolgan, A A A Thomsen, June Lund Pedersen, Kristian Windfeld, Lisa Hellström, Märta Segerdahl Storck, Morten Rosted, Philip Hougaard, Tilde Dyring Weidlich, Carine Djuika, Lutz Frölich, Agnieszka Trubacz, Christopher Weber, Christopher Randolph, Harald Hampel, Michael Ewers, John Harrison, Andrew Donaldson, Donna Cairney, Sheila MacFayden, Tracy Baird, Adrian Mander, Andrea Wadeson, Brian Tom, Costanza Di Fant, Gavin Lingiah, James Howlett, Keith Merle, Lisa Brown, Rebekah Davies, Robin Huang, Simon White, Steven Hill, Farid Chekani, Jina Swartz, Letitia Reyniers, Rezaul Khandker, Tamara Connelly, Laurie Ryan, Elizabeth Coulthard, James Selwood, Natalie Rosewell, Rebecca Cousins, Cynthia Duggan, Catriona McNeill, Derek Brown, Shona McKay, Alasdair Lawrie, Emma Darling, Julie Scott, Kirsten McClelland-Brooks, Justine Hudson, Kate Ferguson, Peter Connelly, Prasad Guntur Ramkumar, Tiffany Stewart, Ana Graf, Barbara Stolz, Dieter Nussher, Etienne Pigeolet, Giulia Lestini, Gwenaelle Fillon, Ines Paule, Marianne Maman, Sema Cheyun, Stephan Korte, Yvonne Madawela, Jeff Kaye, Bruno Dubois, Patrick Feukam Talla, Sana Akel, Baptiste Porte, Julien Dumurgier, Sandrine Indart, Yaël Slama, Alexandra Fayel, Gerald Luscan, Hannah Hurst, James Eshelby, Mariana Gameiro, Max Mirza, Olga Krylova, Olivier Drap, Paul Marrone, Rachel Schindler, Yves Brault, Ewa Iwaniuk, Magdalena Lapinska, Magdalena Maslowska, Jacek Dobryniewski, Michael Gold, M A-LEK - Maciej Maciejowski, Christina Rabe, Enisa Alibasic, Friedemann Krause, Jason Hanon, Joanna Wright, Jyoti Srivastava, Maryline Simon, Richard Batrla-Utermann, Silvia Barbara Schmid, Simone Wahl, Udo Eichenlaub, Verena Neuhold, Ingo Kilimann, Stefan Teipel, Clemens Peter, Dianne Stephanie Van Houdt, Edo Richard, Jordy Birahy, Marthe Smedinga, Reli Pascale, Sonja Bemelmans, Stephanie Beukers, Wim van Oijen Samodzielny Publiczny Szpital Kliniczny, Katarzyna Lucka, Konrad Rejdak, Aleksandra Stjepanovic-Agovic, Caroline Cohen, Cornelia Mockwitz, Frederic Delalonde, Isabelle Clavier, Jean-François Dedieu, Laurence Mazuranok, Nacera Hamdani, Nathalie Piton, Philippe Rocolle, Romain Hahn, Carlos Díaz, Estefanía Callado, Nina Coll, Nuria Subirats, Saira Ramasastry, Sandra Pla, Achim Fischer, Adam Schwarz, Alex Bowtell, Antonella Chiucchiuini, Henrik Andersson, Melissa Naylor, Polyna Khudyakov, Rajan Perinpanayagam, Wei Zhong, Howard Feldman, Tristy Tara, Timo Grimmer, Carol Brayne, David Ash, Dennis Chan, Emma Barham, Guy Casy, John O'Brien, Judith Wilson, Lesley Pilgrim, Michele York, Nadja Smailagic, Natassia Brenman, Peter Geach, Philippa Farmer, Renata Schaeffer, Richard Milne, Sally Atkinson, Sarah Findon, Sarah Taylor, Shirlene Badger, Susan Black, Tazuko Edwards, Will Clark, Michele York, Zeynep Sahin, Babak Boroojerdi, Bernhard Greve, Daphne Derouane, David Marquet, Johannes Streffer, Katharina Angrosch, Mireille Delval, Peter Verplancke, Simon Dresse, Stefano Zanigni, Suzanne McCabe, Nick Fox, Pawel Markiewicz, Joel Kramer, Sabrina Erlhoff, Michael Schaffer, Adam Waldman, Alan Kennedy, Andy Kodiak, Angela Noble, Anna Anderson, Anna Borthwick, Anne Hall, Bill Bruce, Brian McTeir, Carol Di Perri, Cathie Sudlow, Chloe Kippen, Clare Dolan, Craig Ritchie, David Hill, David Matheson, Doug Young, Eirini Souri, Ellie Mcmaster, Emilie Delpon, Emma Law, Emma Munro, Fiona Campbell, Fiona Edler, Fiona Scott, Graciela Muniz Terrera, Hannah Jobse, Hazel Milligan, Hinesh Topiwala, Howard Marriage, Jean Manson, Jen Middleton, Joanna Swiderska, Joanna Wardlaw, Joanne MacConnell, Judi Syson, Julie Edwards, Kate Forsyth, Kathryn Carruthers, Katia Hervy, Katie Wells, Kristy Draper, Laura Doull, Laura Petrie, Lesley Niezynski, Lindsay Hampton, Loukia Koutsoventi, Lucy Stirland, Marise Bucukoglu, Molly Boughey, Natalie Jenkins, Neil Duncan, Neil Fullerton, Neil Mitchell, Ray French, Rebecca Flory, Ruaridh Buchan, Rustam Al Shahi-Salman, Samuel Danso, Sarah Gregory, Sarah Sparks, Stina Saunders, Susan Shepherd, Tamlyn Watermeyer, Tom MacGillivray, Tom Russ, Tyler Saunders, Val Renton, Vikki Leslie, Alexander Dzerzga, Britta Doelle, Christel Gatzke, Clause Escher, Denise Schneider, Lena Sannemann, Manuela Thelen, Petra Schreiner-Kaub, Simone Stockter, Susan Thielking, Theresa Mueller, Frank Jessen, Andrea Toja, Elena Chipi, Lucia Farotti, Lucilla Parnetti, Owen Lancaster, Anthony J Brookes, Brian Berry, Colin Veal, Dhiwagaran Thangavelu, Jon Sole, Marie Adams, Monika Maini, Nickie Ketcher, Nicola A Ketcher, Vagelis Ladas, Alexandra Agogue, Ana Campillo, Andreas Schmalz, Bianca Auschra, Blanche Pirotte, Christine Trombert, Daria Genaro, Elisa Canzoneri, Emiliano Albanese, Estefania Vilarino, Giovanni Battista Frisoni, Jennyfer Sturzmann, Margherita Mauri, Marina Boccardi, Mathilde Boillat, Maura M P Parapini, Monica Almici, Paulina Andryszak, Sven Haller, Tatjana Stevanovic, Christopher Kipps, Susan Jackson, Charlotte Frick, Henrik Zetterberg, Kaj Blennow, Linnea Petersson, Maria Berglund, Silke Kern, Alexandra Radford, Annalena Venneri, Joanne Sidebottom, Patrick Easton, Rosie Clegg, Walter Kullur, Tom Hartley, Amy Chinner, Antoniya Markova, Ash Sexton, Caroline Jenkins, Clare Mackay, Corinne Prescott, David Ruvolo, Delia Gheorghe, Denis Murphy, Gem Pope, Gill Cane, Gill Wells, Ivan Koychev, Jasmine Blane, Jennifer Lawson, John Gallacher, Juliana Ballaminut, Justin Lowen, Karla Westphal, Katherine Shepherd, Kay McNamee, Leona Wolters, Michael Ben Yehuda, Natalie Morgan, Neil Buckholtz, Nyla Haque, Patricia Burton, Sarah Bauermeister, Sarah Hoosdally, Shona Forster, Tequila Osborne, Vanessa Raymont, Ann van den Eynde, Sebastiaan Engelborghs, Rik Vandenberghe, Carine Schildermans, Alle Meije Wink, André Van der Wal, Aniko Hazelebach, Anna Elisabeth Leeuwis, Casper De Boer, Fiona Heeman, Frederik Barkhof, Isadora Lopes-Alves, Jeroen van Leur, Lea ter Meulen, Lisa Vermunt, Luigi Lorenzini, Lyduine Eisa Collij, Mara ten Kate, Martijn van Houten, Menno Stellingwerff, Merel Hermans, Michel Telkamp, Niels Prins, Philip Scheltens, Pieter Jelle Visser, Silvia Ingala, Wendy van Veen, Michelle McDonald, Clare Taylor, Genevieve Morrison, Mara Golemme, Paresh Malhotra, Sithuya Mahalingam, Christine van Broechhoven, Johan Goeman, Sebastiaan Engelborghs, Wendy Wittebolle

**Affiliations:** Department of Neurology, Massachusetts General Hospital, Boston, MA 02114, USA; Department of Neurology, Harvard Medical School, Boston, MA 02114, USA; Department of Neurology, Massachusetts General Hospital, Boston, MA 02114, USA; Department of Neurology, Harvard Medical School, Boston, MA 02114, USA; Martinos Center for Biomedical Imaging, Massachusetts General Hospital, Charlestown, MA 02129, USA; Computer Science and AI Laboratory, Massachusetts Institute of Technology, Cambridge, MA 02138, USA; Department of Neurology, Harvard Medical School, Boston, MA 02114, USA; Martinos Center for Biomedical Imaging, Massachusetts General Hospital, Charlestown, MA 02129, USA; Computer Science and AI Laboratory, Massachusetts Institute of Technology, Cambridge, MA 02138, USA; Centre for Medical Image Computing, University College London, London WC1V 6LJ, UK; Department of Neurology, Massachusetts General Hospital, Boston, MA 02114, USA; Department of Neurology, Harvard Medical School, Boston, MA 02114, USA; Department of Neurology, Massachusetts General Hospital, Boston, MA 02114, USA; Department of Neurology, Harvard Medical School, Boston, MA 02114, USA; Department of Neurology, Massachusetts General Hospital, Boston, MA 02114, USA; Department of Neurology, Harvard Medical School, Boston, MA 02114, USA; Department of Neurology, Massachusetts General Hospital, Boston, MA 02114, USA; Department of Neurology, Harvard Medical School, Boston, MA 02114, USA; Department of Neurology, Massachusetts General Hospital, Boston, MA 02114, USA; Department of Neurology, Harvard Medical School, Boston, MA 02114, USA; Department of Neurology, Massachusetts General Hospital, Boston, MA 02114, USA; Department of Neurology, Harvard Medical School, Boston, MA 02114, USA; Department of Neurology, Massachusetts General Hospital, Boston, MA 02114, USA; Department of Neurology, Harvard Medical School, Boston, MA 02114, USA; Department of Neurology, Massachusetts General Hospital, Boston, MA 02114, USA; Department of Neurology, Harvard Medical School, Boston, MA 02114, USA

**Keywords:** brain volumetric change, CSF core Alzheimer’s disease biomarkers, morphometric connectome, predictive modelling, sex differences

## Abstract

The relationship between cerebrospinal fluid (CSF) biomarkers of Alzheimer’s disease and neurodegenerative effects is not fully understood. This study investigates neurodegeneration patterns across CSF Alzheimer’s disease biomarker groups, the association of brain volumes with CSF amyloid and tau status and sex differences in these relationships in a clinical neurology sample. MRI and CSF Alzheimer’s disease biomarkers data were analysed in 306 patients of the Mass General Brigham healthcare system aged 50+ (mean age = 68.4 ± 8.8 years; 43.1% female), who had lumbar punctures within 1 year of clinical MRI scans. We first analysed neurodegeneration patterns across four biomarker groups: 60 controls (A−T−&CU; amyloid negative, tau negative, cognitively unimpaired), 25 A+T− (amyloid positive, tau negative), 121 A+T+ (amyloid positive, tau positive) and 100 other dementia (A−T−&CI; amyloid negative, tau negative, cognitively impaired). Second, we examined volumetric associations with amyloid (amyloid positive, tau negative versus control) and tau in the presence of amyloid (amyloid positive, tau positive versus amyloid positive, tau negative) across 52 brain areas. Third, we examined sex differences in these relationships. Finally, we validated core analyses across three independent datasets—NACC (National Alzheimer’s Coordinating Center), ADNI (Alzheimer’s Disease Neuroimaging Initiative) and EPAD (European Prevention of Alzheimer’s Dementia)—totalling 3137 participants, and performed meta-analyses to obtain more robust estimates. We observed distinct neurodegeneration patterns across biomarker groups, with disrupted connectivity (brain volume covariance networks) in amyloid positive and other dementia groups, while amyloid and tau negative, cognitively unimpaired controls exhibited the most connected network. Amyloid was associated with subcortical, cerebellar and brainstem atrophy, with consistent association observations in the thalamus and amygdala across all four datasets. Tau in the presence of amyloid demonstrated general brain shrinkage through enlargement of extracerebral CSF, alongside unexpected ventricle shrinkages. Sex-based analyses revealed that A+T+ (amyloid positive, tau positive) had lower sex differences in connectivity patterns compared with other groups. Sex differences were also noted in amyloid-related ventricular volume changes. This study reveals how amyloid and tau affect brain connectivity and volume across sex and CSF biomarker groups, emphasizing global brain changes and sex differences. By leveraging automated pipelines and advanced MRI and biomarker analyses, we extracted meaningful and replicable findings from heterogeneous clinical samples from real-world data. The meta-analyses across four datasets enhance the generalizability of our results.

## Teaser

Cheng *et al*. report that analysing structural brain changes across four large datasets, using advanced segmentation, reveals robust associations of Alzheimer’s disease CSF biomarkers (amyloid, tau) with brain volumes, morphometric connectomes and sex differences. They conclude that integrating imaging with biomarker profiling yields a comprehensive understanding of disease progression.

## Graphical Abstract

**Graphical Abstract:**
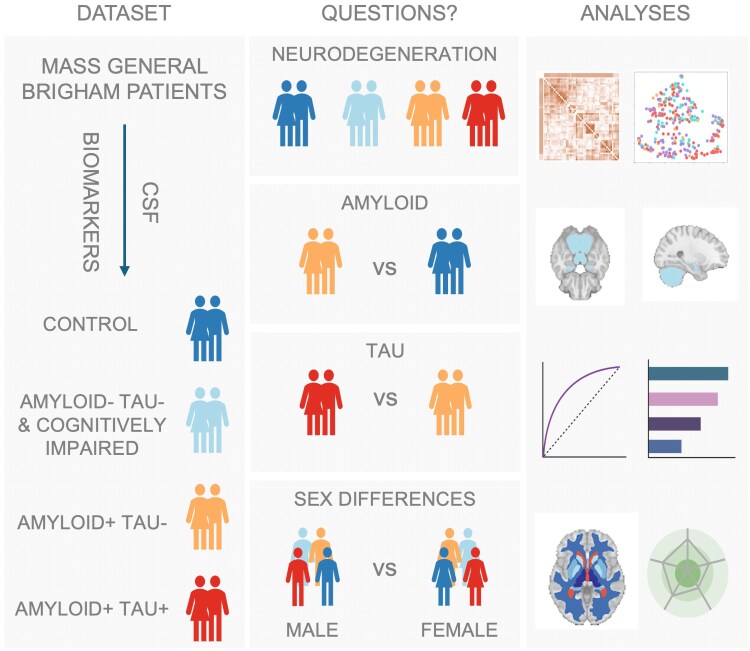

## Introduction

Amyloid and tau are hallmark pathologies of Alzheimer’s disease^1,2^ and have been identified as core biomarkers for Alzheimer’s disease diagnosis, according to the most recent diagnostic criteria published by the Alzheimer’s Association.^3^ The measurement of amyloid and tau in CSF, which demonstrates comparable performance to positron emission tomography (PET) imaging,^4-6^ is becoming increasingly widespread for Alzheimer’s disease diagnosis. However, the relationship between CSF biomarkers and brain neurodegeneration patterns has not yet been fully explored.

Many studies have examined the associations between core Alzheimer’s disease biomarkers and brain atrophy, primarily focusing on amyloid and tau deposition measured by PET imaging. These studies have shown complex relationships between amyloid and brain structure. For instance, global amyloid deposition often correlates with hippocampal atrophy,^7,8^ while regional amyloid deposition can show positive,^9^ negative^8,9^ or no correlation^8,9^ with brain atrophy, depending on the brain region. In contrast, tau-related atrophy more consistently aligns with the distribution of neurofibrillary tangles observed in post-mortem studies,^10-12^ especially in the medial temporal lobe^7-9^. While PET imaging can reveal both total and regional insoluble, fibrillar amyloid and tau accumulation,^13^ less is known about how CSF Alzheimer’s disease biomarkers,^3,14,15^ which reflect net production and clearance rates of soluble amyloid and tau species, relate to brain atrophy. Some studies have found that CSF tau is associated with hippocampal atrophy, while CSF amyloid is not.^16^ Others have reported associations between both core biomarkers and hippocampal atrophy^17,18^ or with whole brain volume—amyloid in controls and p-tau in Alzheimer’s disease dementia.^19^ CSF amyloid, not p-tau, was also associated with ventricular enlargement in preclinical Alzheimer’s disease.^20-23^

The revised Alzheimer’s disease diagnostic criteria highlight that amyloidosis is essentially a prerequisite of Alzheimer’s disease tauopathy,^3^ underscoring the need to study how brain volumes associate with tau in the presence of amyloid. However, only a few studies have investigated the combined effects of CSF amyloid and p-tau on brain volumes, and these were limited to cognitively unimpaired populations. For instance, one study found that elevated p-tau was associated with smaller volumes of the hippocampus, amygdala and entorhinal cortex only in amyloid positive individuals.^24^ Another study reported that individuals with amyloid positivity had higher regional volumes compared with controls without neurodegeneration.^25^ Additionally, the impact of CSF Alzheimer’s disease biomarker groups on grey matter structural connectivity (aka. morphometric connectome) remains underexplored. Although one study identified associations between amyloid status, p-tau levels and structural connectivity metrics such as clustering coefficients,^26^ there has been no direct comparison across groups defined by distinct CSF biomarker groups. Furthermore, despite evidence that amyloid affects hippocampal volume more in females than in males,^27^ sex differences in brain volumes across CSF Alzheimer’s disease biomarker groups have been understudied. Understanding how amyloid and tau pathology differently influence brain volumes in males and females could advance personalized diagnostic and treatment approaches.

In this study, we first examined differences in neurodegeneration patterns across four CSF Alzheimer’s disease biomarker groups: (i) control group defined as A−T−&CU (amyloid negative, tau negative, cognitively unimpaired); (ii) A+T− (amyloid positive, tau negative); (iii) A+T+ (amyloid positive, tau positive) and (iv) A−T−&CI (amyloid negative, tau negative, cognitive impairment due to other non-Alzheimer’s disease conditions). We then investigated the association of brain volumes with amyloid (A+T− versus control) and tau in the presence of amyloid (A+T+ versus A+T−). Finally, we examined sex-based variations in brain volumes across CSF Alzheimer’s disease biomarker groups. Core analyses were conducted using biomarker, clinical and imaging data from participants in the MIND (MassGeneral Institute for Neurodegenerative Diseases) research biobank of the Mass General Brigham (MGB) healthcare system and replicated in three independent samples from the NACC (National Alzheimer’s Coordinating Center),^28^ the ADNI (Alzheimer’s Disease Neuroimaging Initiative)^29^ and the EPAD (European Prevention of Alzheimer’s Dementia)^30^ studies.

## Materials and methods

### MGB patient data

#### Image acquisition and analyses

All image data were pre-existing clinical images from the MGB patient database that are within 1 year of the date of patients’ lumbar puncture, aged 50 years old or above and without early onset autosomal dominant Alzheimer’s disease (*n* = 328). Brain segmentation and volume calculation were performed via the SynthSeg+ pipeline—a deep learning algorithm^31^ for volumetric segmentation of clinical brain images with various contrast and resolutions into subcortical areas, cortical areas, ventricles, cerebellum, brain stem and extracerebral CSF. SynthSeg+ detects its own segmentation failures (e.g. due to insufficient field of view or image quality) via quality control (QC) scores that are automatically estimated for each of the aforementioned regions. The QC scores are defined between zero and one; images with average QC scores from all subcortical regions being above 0.65 were kept. If a patient had multiple clinical images in the same session, we calculated the average brain volume of all images satisfying the QC constraint as the final brain volume for the patient. Volume from each brain area were adjusted with the intracranial volume (estimated by SynthSeg+) by division. To reduce the number of variables and improve the robustness of the regression by minimizing overfitting, we combined all bilateral volumes; we also calculated prefrontal cortex volumes by combining all prefrontal subregions, yielding volumes for a total of 52 brain regions.

#### CSF collection and analysis

CSF data were obtained from the MIND biobanking study. In this study initiated in 2015, all patients who underwent lumbar puncture in the outpatient Neurology Clinical of Massachusetts General Hospital are approached for consent to bank excess or additional (5cc) CSF for research purposes. CSF levels of Aß40, Aß42 and p-Tau181 were measured at the MIND Biomarker Core using Euroimmun immunoassays (Lübeck, Germany), as previously described.^32,33^ Amyloid status was determined using ABR (Aß42/40 ratio), with a threshold of ABR (Aß42/40 ratio) < 0.082 indicating A+ (amyloid positive), and ABR (Aß42/40 ratio) ≥ 0.082 indicating A− (amyloid negative). Tau status was based on p-Tau181 levels, with concentrations > 41.8 pg/mL classified as T+ (tau positive), and concentrations ≤ 41.8 pg/mL classified as T− (tau negative). These thresholds were derived in-house using samples from cognitively unimpaired individuals (*n* = 358) and individuals with a clinical diagnosis of Alzheimer’s disease verified by CSF Alzheimer’s disease biomarkers in clinical testing (Athena ADmark; *n* = 155) and were set at the point where the sensitivity and specificity were equal (91% sensitivity and specificity for both assays). Our control group, classified as having normal Alzheimer’s disease biomarkers, was determined as A−T− (amyloid negative, tau negative) and clinically diagnosed as cognitively unimpaired. The other dementia group was classified as having normal Alzheimer’s disease biomarkers (A−T−, amyloid negative, tau negative) but with cognitive impairment. Cognitive assessments were conducted by a neurologist and psychiatrist specializing in Alzheimer’s disease and related dementia (ADRD) diagnosis through a systematic chart review.

### Statistical analyses

All analyses and plots, except for the structural covariance network (SCN) analyses and functional network correspondence analyses, were performed using R (version 4.2.1).

### Patterns of neurodegeneration across CSF Alzheimer’s disease biomarker groups

All analyses were conducted in each of the four CSF Alzheimer’s disease biomarker groups—control (amyloid negative, tau negative, cognitively unimpaired), A+T− (amyloid positive, tau negative), A+T+ (amyloid positive, tau positive) and other dementia (amyloid negative, tau negative, cognitive impairments)—separately. These analyses included correlation analyses, SCN analyses, high-dimensional clustering, unitary analyses, validation with three independent datasets, meta-analyses and functional network correspondence. To examine sex differences in patterns of neurodegeneration and brain volumes associated with each CSF Alzheimer’s disease biomarker group, we performed the same analyses separately for males and females.

#### Correlation analyses

To study the relationships between brain volumes, we first carried out partial bivariate Pearson correlations using the ‘pcor’ function in the ‘ppcor’ package (version 1.1). These controlled for age and sex, covering brain volume measurements in patients 50 years and older. We performed the correlation analyses for distinct CSF Alzheimer’s disease biomarker groups and then converted all correlation coefficients to Fisher *z*-scores for easier group comparisons. The results were plotted using the ‘heatmap’ function in the R ‘ComplexHeatmap’ package (version 2.15.1).

#### SCN analyses

To elucidate the organizational patterns of the brain volumes, we analysed the SCN of brain volume. In this context, connectivity refers to the degree to which the volumes of various brain regions co-vary, indicating coordinated growth or atrophy patterns among these regions. Our analyses aimed to explore both the local and global organizational principles of the brain, offering insights into how brain regions co-vary in size across individuals with and without Alzheimer’s disease pathology. These included the global clustering coefficients, which shed light on the network’s tendency to form tightly-knit groups (clusters) by evaluating the extent of clustering among nodes (brain regions). We also examined the path length, providing insight into the average shortest distance between all pairs of nodes, offering insight into the network’s overall navigability and efficiency in communication across the entire brain. Global efficiency was assessed to understand the effectiveness of information exchange across the entire network by evaluating how efficiently information is integrated globally. On a more granular level, we measured the nodal degree to determine the number of direct connections each individual node (brain region) has within the network, indicating its level of connectivity, alongside nodal clustering coefficients and nodal efficiency, which respectively evaluate the propensity for local clustering around individual nodes (brain regions) and their efficiency in facilitating information flow. We also measured small-worldness, calculated by dividing the global clustering coefficients by the average path length. This ratio reflects the efficiency of the network in balancing local clustering with short paths for global communication, where higher values indicate a more optimal small-world structure, characterized by efficient information transfer both locally and across the network. The analyses were conducted for each of the distinct CSF Alzheimer’s disease biomarker groups. To ensure meaningful computation of path length and to avoid infinite values resulting from disconnected networks, we retained the top 35% of the strongest connections in the adjacency matrix, thereby ensuring that the SCN was fully connected. Non-parametric Wilcoxon tests were applied to compare SCN metrics between CSF Alzheimer’s disease biomarker groups due to non-Gaussian data distributions, with correction for multiple comparisons. All SCN metrics were calculated using ‘bct’ package (version 0.6.0) and visualized with ‘nilearn’ package (version 0.10.2) from Python 3.10.13.

#### High-dimensional analysis of brain volumes

To explore the high-dimensional, non-linear relationships among brain volumes, we used the ‘umap’ package (version 0.2.10.0) to create UMAP (uniform manifold approximation and projection) visualizations. These projections were generated for the entire brain to capture global volumetric patterns, as well as separately for each brain lobe, subcortical grey matter and other individual structures. UMAP clusters were generated for each CSF Alzheimer’s disease biomarker group, and the first two UMAP components (UMAP1 and UMAP2) were used for visualization, as they capture the most meaningful variation in the data while preserving local and global structures in a low-dimensional space. To compare clustering across different brain regions based on CSF Alzheimer’s disease biomarkers, we calculated the SGCC (standardized global clustering coefficient). This distance-based metric is calculated by taking the difference between the average distance between points in different groups (between-category distance) and the average distance between points within the same category (within-category distance). This difference is then divided by the larger of the two average distances, yielding a value between −1 and 1. A value of 1 indicates that brain volumes are well-separated by CSF Alzheimer’s disease biomarker groups, with strong clustering within their own category and poor overlap with neighbouring groups, suggesting a clear distinction between groups. A value of 0 indicates that brain volumes are positioned near the boundary between two groups, suggesting ambiguity in clustering, while a negative value suggests that brain volumes may be incorrectly grouped, as they are closer to a neighbouring biomarker category than their own.

### Brain volume association with CSF Alzheimer’s disease biomarkers

All analyses were conducted for amyloid status—A+T− (amyloid positive, tau negative) versus control (amyloid negative, tau negative, cognitively unimpaired)—and tau in the presence of amyloid—A+T+ (amyloid positive, tau positive) versus A+T− (amyloid positive, tau negative)—separately.

#### Unitary analyses: logistic regression

To test the cross-sectional association of brain volumes and CSF Alzheimer’s disease core biomarkers, we conducted logistic regression for each brain volume separately, adjusting for age and sex. The analyses were conducted using the ‘glm’ function in the ‘stats’ package (version 4.2.1). Brain volumes that showed statistically significant differences (uncorrected *P* < 0.05) between comparison groups were selected as features for the machine learning model. Comparison groups include: A+T− (amyloid positive, tau negative) versus control (amyloid negative, tau negative-cognitively unimpaired) and A+T+ (amyloid positive, tau positive) versus A+T− (amyloid positive, tau negative).

#### Machine learning

Our binary classification method to ascertain participants’ Alzheimer’s disease core biomarkers’ status leveraged four classifiers: LASSO (least absolute shrinkage and selection operator) logistic regression, ridge logistic regression, Firth logistic regression and random forest. These classifiers were chosen for their capability to assess feature importance. Logistic regressions’ feature importance was determined by the size of standardized coefficients, while for the random forest, it was based on the permutation-based mean decrease in accuracy (MDA).

The MRI datasets contained intracranial volume-adjusted 52 brain volumes which were first scaled to have a mean of 0 and a standard deviation of 1. We divided the data into a training set (75%) and a held-out test set (25%) for each classifier. The models were trained using three times repeated 3-fold cross-validation, ensuring that each fold had a similar proportion of positive cases (about 59% in the amyloid status classification and 58% in the tau status classification). Model performance was determined by the cross-validation mean performance, scrutinizing their positivity detection accuracy.

For the logistic regression, we implemented either L1 regularization (LASSO), L2 regularization (ridge) or Firth’s logistic regression to avoid overfitting and address bias in small samples. The regularization strength (i.e. lambda) for L1 and L2 regularization was fine-tuned from 0.001 to 0.1 in 0.001 increments, using the same repeated 3-fold cross-validation process where a subset of the training data in each fold was used. This helped pinpoint the optimal regularization level, allowing the model to select features that exhibit a strong relationship with predictors. Firth’s logistic regression was utilized to reduce bias in parameter estimates, particularly useful for small sample sizes and rare events, by adjusting the likelihood function to provide more accurate estimates. For Firth’s logistic regression, the maximum number of iterations was set to 100 for both the penalized likelihood control and the logistic regression control to ensure convergence. The random forest classifier underwent a grid search, also using the same repeated 3-fold cross-validation process, to optimize the number of predictors at each split, ranging from 1 to 10 in increments of 1. We fixed the tree count at 500, using all the features in the model. Trees were grown to maximum depth, with each using a bootstrap sample of about 63.2% of the training data. The resampling method was implemented in the models to ensure a balance of different classes. Note that the decision thresholds in our models were set to the default value of 0.5, meaning that the provided accuracy, sensitivity and specificity in the table are based on this standard threshold without any adjustments to balance false positives and false negatives. This modelling was executed with R’s ‘caret’ and ‘logistf’ package.

For both the logistic regression and random forest classifiers, we evaluated the accuracy of the predictions by calculating the AUROC (area under the receiver operating curve) and its 95% confidence intervals, which were estimated using 2000 bootstrap samples. We also assessed the sensitivity and specificity of the test set. The same machine learning techniques were implemented to differentiate between individuals based on their amyloid and tau biomarker statuses, classifying them according to their respective positivity. Multiple metrics were evaluated.

#### Validation

Validation allowed us to evaluate the generalizability of the associations of CSF Alzheimer’s disease biomarkers—‘A’ (amyloid) and ‘T’ (tau)—with brain volumes across different assay techniques in the different samples. See ‘Methods—validation datasets’ in the Supplementary material for details of the three validation datasets.

#### Meta-analyses

To synthesize findings across datasets, we conducted a random-effects meta-analysis, which accounts for variability in effect sizes between datasets, on brain volume features significantly associated with core Alzheimer’s disease biomarkers in at least one dataset. Using the ‘rma’ function from the ‘metafor’ package^34^ (version 4.6-0) with restricted maximum-likelihood estimation (REML), we calculated pooled effect sizes and associated statistics for each relevant feature. We applied multiple comparison corrections to ensure the robustness of findings across these features. This approach aggregated results from multiple studies, offering a quantitative summary of overall effects and their variability. By identifying consistent patterns across datasets, the meta-analysis enhanced our understanding of reliable structural indicators of Alzheimer’s disease pathology, providing a more robust assessment of brain changes associated with Alzheimer’s disease biomarkers.

#### Functional network correspondence

We used brain volumes significantly associated with Alzheimer’s disease core biomarkers from meta-analyses to explore their relationship with established functional networks. By applying the NCT^35^ (Network Correspondence Toolbox, ‘cbig_network_correspondence’ package version 0.2.1) in Python, we quantified the spatial overlap between these volumes and networks using Dice coefficients and evaluated statistical significance through spin tests. This analysis revealed the overlap between structural changes and functional networks, providing insights into the potential neural substrates underlying Alzheimer’s disease pathology and its clinical manifestations.

#### Statistical considerations

All statistical tests were two-tailed unless otherwise noted, with *α* = 0.05 set for significance. Residual and diagnostic plots were examined where applicable to ensure model assumptions (e.g. linearity in the logit for logistic regressions, homoscedasticity for correlation analyses), and non-parametric methods (e.g. Wilcoxon tests) were used for non-Gaussian data; multiple comparison corrections were applied for network metrics and other comparisons as detailed in each subsection. Randomization and blinding were not applicable given the observational design, and the experimental unit was the individual participant (each contributing a single set of brain volume measures to avoid pseudo-replication). No *post hoc* power analyses were performed, and repeated cross-validation was employed to optimize hyperparameters and assess predictive accuracy for logistic regression and machine learning models. Exact *P*-values and 95% confidence intervals are provided in the Results section.

## Results

### Study dataset

**Figure 1:**
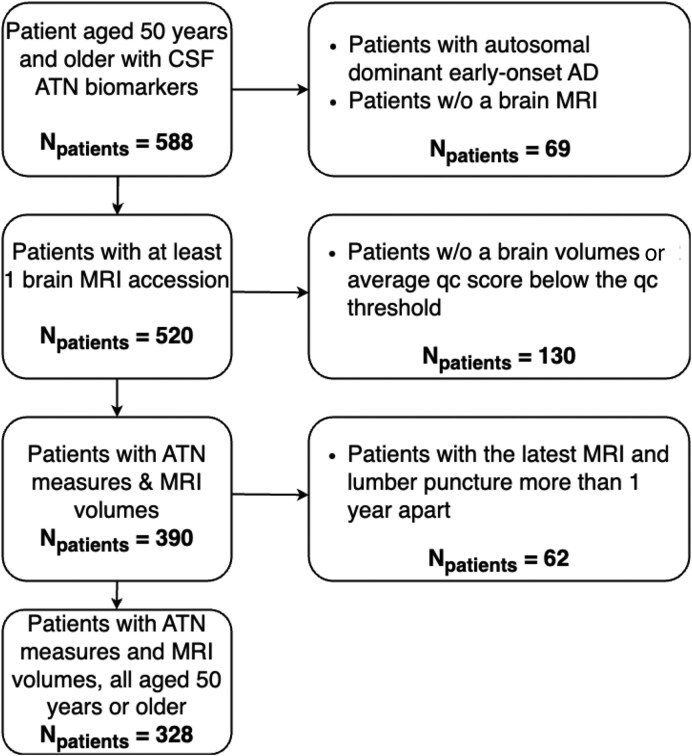
**The MGB dataset.** Consort diagram of the patient selection process. ATN, amyloid/tau/neurodegeneration; AD, Alzheimer’s disease; w/o, without; qc, quality control.

The MGB dataset, used as a discovery set, comprised 306 patients (excluding the 22 A−T+ cases), all 50 years of age or older, selected from the MGB healthcare system. Details of the patient selection process are illustrated in Fig. 1, the age distribution of the participants is shown in Supplementary Fig. 1. The average age of the study population was 68.4 ± 8.8 years, with 43.1% being female. The majority of the patients identified as White (93.8%) and not Hispanic or Latino (89.2%). 17.0% of the participants had 12 years or fewer of education, 6.5% had 13–16 years of education and 61.4% had attained 17 years or more of education. In terms of CSF Alzheimer’s disease biomarker group, 19.6% (*n* = 60) of the patients were cognitively unimpaired non-Alzheimer’s disease control (i.e. A−T− or amyloid negative, tau negative), 8.2% (*n* = 25) were A+T− (amyloid positive, tau negative), 39.5% (*n* = 121) were A+T+ (amyloid positive, tau positive) and 32.7% (*n* = 100) were A−T− (amyloid negative, tau negative) biomarker with CI (cognitive impairment). There were no missing data on age or sex. However, race data were missing for 3.3% of patients, ethnicity for 7.5% and educational background for 15.0%. There were no gaps in the data for brain volumes. For more detailed information, refer to Table 1. For information on the demographic characteristics in the validation datasets, refer to Supplementary Table 1A–C. The clinical characteristics of the A−T−&CI (amyloid negative, tau negative, cognitive impairment) group are summarized in Supplementary Table 1D.

**Table 1:** Summary statistics of the demographic characteristics in the MGB dataset

Characteristics	Total (*n* = 306)	Control (*n* = 60; 19.6%)	A+T− (*n* = 25; 8.2%)	A+T+ (*n* = 121; 39.5%)	A−T−&CI (*n* = 100; 32.7%)
Age, mean (SD), years					
	68.4 (8.8)	63.1 (9)	74.8 (9.4)	69.2 (8.2)	68.9 (7.8)
Sex, *N* (%)					
Female	132 (43.1)	34 (56.7)	12 (48.0)	46 (38.0)	40 (40.0)
Male	174 (56.9)	26 (43.3)	13 (52.0)	75 (62.0)	60 (60.0)
Race, *N* (%)					
White	287 (93.8)	55 (91.7)	23 (92.0)	116 (95.9)	93 (93.0)
Black or AA	5 (1.6)	2 (3.3)	0 (0.0)	1 (0.8)	2 (2.0)
Asian	3 (1.0)	0 (0.0)	0 (0.0)	1 (0.8)	2 (2.0)
AI or AN	1 (0.3)	1 (1.7)	0 (0.0)	0 (0.0)	0 (0.0)
Not available	10 (3.3)	2 (3.3)	2 (8.0)	3 (2.5)	3 (3.0)
Ethnicity, *N* (%)					
Not Hispanic or Latino	273 (89.2)	55 (91.7)	21 (84.0)	103 (85.1)	94 (94.0)
Hispanic or Latino	10 (3.3)	3 (5.0)	1 (4.0)	4 (3.3)	2 (2.0)
Not available	23 (7.5)	2 (3.3)	3 (12.0)	14 (11.6)	4 (4.0)
Education, *N* (%)					
≤12 years	52 (17.0)	13 (21.7)	4 (16.0)	15 (12.4)	20 (20.0)
13–16 years	20 (6.5)	8 (13.3)	0 (0.0)	8 (6.6)	4 (4.0)
17+ years	188 (61.4)	34 (56.7)	18 (72.0)	76 (62.8)	60 (60)
Not available	46 (15.0)	5 (8.3)	3 (12.0)	22 (18.2)	16 (16.0)

The mean age of this cohort was 68.4 ± 8.8 years old, and 43.1% were women. A majority of the cohort identify as White (93.8%) and not Hispanic or Latino (89.2%). 17.0% of the participants had 12 years or fewer of education, 6.5% had 13–16 years of education and 61.4% had attained 17 years or more of education. In terms of CSF Alzheimer’s disease biomarker group, 19.6% (*n* = 60) were control (i.e. A−T−&cognitively unimpaired), 8.2% (*n* = 25) of the patients were A+T−, 39.5% (*n* = 121) were A+T+ and 32.7% (*n* = 100) were A−T−&CI. AA, African American; AI, American Indian; AN, Alaska Native; A+T−, amyloid positive, tau negative; A+T+, amyloid positive, tau positive; A−T−&CI, amyloid negative, tau negative and cognitive impaired; MGB, Mass General Brigham.

### Neurodegeneration patterns and structural connectivity across CSF Alzheimer’s disease biomarker groups

**Figure 2:**
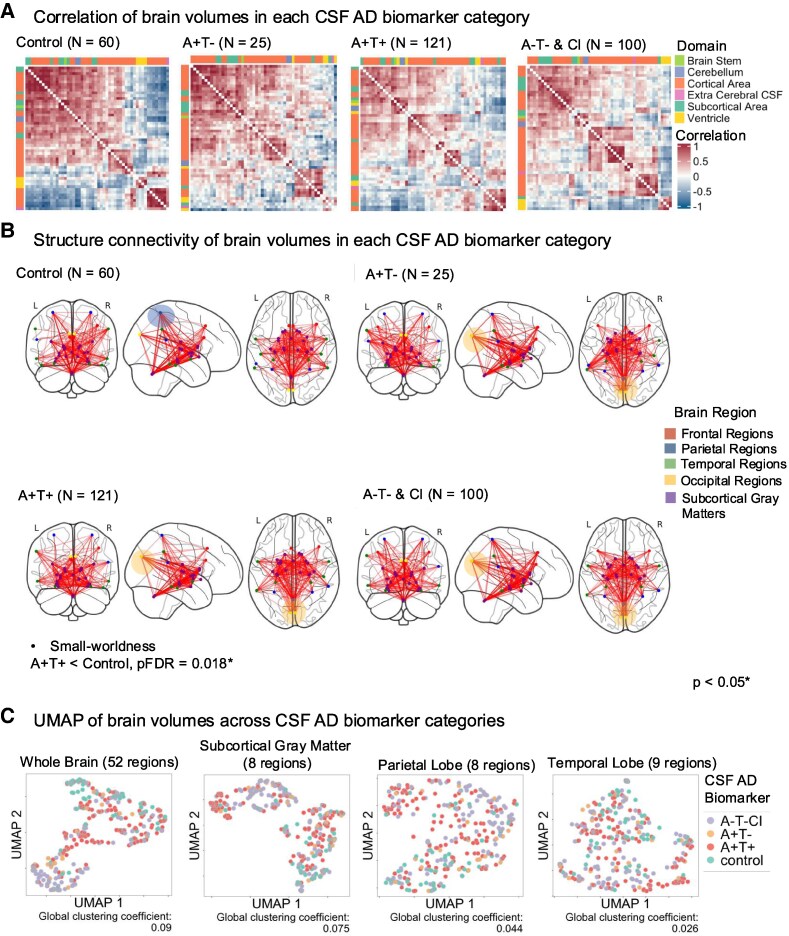
**Neurodegeneration patterns and morphometric connectome across CSF Alzheimer’s disease biomarker groups in the MGB dataset.** Patterns of neurodegeneration in different CSF biomarker categories in the MGB dataset. (**A**) Partial bivariate Pearson correlation of brain volumes in the control group (*N* = 60), A+T− group (*N* = 25), A+T+ group (*N* = 121) and the A−T−&CI group (*N* = 100). Compared with groups with positive Alzheimer’s disease biomarkers or cognitive impairment, the control group exhibited more distinct and robustly correlated clusters. Colour annotations above and to the left of each figure represent the brain regions’ categories (i.e. subcortical area, cortical area, cerebellum, ventricle, brain stem and extracerebral CSF). All correlation coefficients were adjusted for age and sex and were Fisher transformed. (**B**) Visualization of the SCN from the top 10% strongest connections from each group of distinct CSF biomarker categories. The control group had more connections between subcortical and parietal regions, contributing to greater small-worldness than the A+T+ group (*pFDR* = 0.018, Wilcoxon test), indicating a more integrated network structure. Yet, there appear to be less direct connections between occipital regions and temporal regions as well as between occipital regions and subcortical grey matters in the control group compared with the other three groups. Yellow circle: A+T−, A+T+ and A−T−&CI groups had more connections between occipital lobe and temporal lobe, as well as occipital lobe and subcortical grey matter than the control and A−T−&CI groups. Blue circle: control had more direct connection between parietal lobe and subcortical grey matters. Note: Connection patterns were based on visual inspection only; no statistical comparisons were conducted for regional edge differences. (**C**) UMAP of whole brain and subregions (*N* = 306) characterized by CSF biomarker categories. The SGCC quantifies separation between categories, with higher positive values indicating greater separation. The global clustering coefficient at the whole brain level (*SGCC* = 0.09) was higher than that in subregions (*SGCC*: 0.026–0.075). Each data point represents the brain volume of an individual participant projected into the UMAP 2D space. AD, Alzheimer’s disease; A+T−, amyloid positive, tau negative; A+T+, amyloid positive, tau positive; A−T−&CI, amyloid negative, tau negative and cognitive impaired; UMAP, uniform manifold approximation and projection; MGB, Mass General Brigham; L, left hemisphere; R, right hemisphere; *pFDR*, false discovery rate-corrected *P*-value.

To examine brain volume atrophy patterns among different CSF Alzheimer’s disease biomarker groups, we conducted partial bivariate Pearson correlations of brain volumes for each group, adjusting for age and sex (Fig. 2A). The control group exhibited the most well-correlated structural network, whereas the A−T−&CI (amyloid negative, tau negative, cognitive impairment) group and the Alzheimer’s disease groups with biomarker categories indicating only amyloid pathology (A+T−, amyloid positive, tau negative) or both amyloid and tau pathology (A+T+, amyloid positive, tau positive) demonstrated distinct correlation patterns, though these were weaker compared with the control group. These qualitative differences suggest disruptions in structural connectivity associated with AD-related pathologies.

Further SCN analysis highlighted differences between the groups with and without Alzheimer’s disease pathology (Table 2). Specifically, the control group displayed significantly higher small-worldness than the A+T+ (amyloid positive, tau positive) groups (*pFDRs* = 0.018), indicating a more efficient network balance between local clustering and global integration. This suggests that the control group’s brain networks are better organized for specialized processing and effective communication across regions. Visualization of the strongest connections in brain regions with the highest nodal degree (Fig. 2B) reinforced these results, showing that the control group had more direct connections between parietal regions and subcortical grey matters than the A+T+ (amyloid positive, tau positive) group. Conversely, the control group appears to have less connections between occipital regions and temporal regions, as well as between occipital regions and subcortical grey matter than the other groups. Note that ‘connection’ here refers to structural covariance between two brain regions, reflecting how the volumes of these regions co-vary across individuals.

**Table 2:** Structural connectivity network metrics of brain volumes in each CSF Alzheimer’s disease biomarker group

Group (*N*)	Global structural connectivity network metrics			Local structural connectivity network metrics						Small-worldness (Mean ± SD)	
	Global efficiency	Path length	Global clustering coefficients	Nodal efficiency (Mean ± SD)		Nodal clustering coefficients (Mean ± SD)		Nodal degree (Mean ± SD)			
Control (*N* = 60)	0.639	1.845	0.658	versus A+T−	0.814 ± 0.107	versus A+T−	0.658 ± 0.154	versus A+T−	17.23 ± 9.15	versus A+T−	0.381 ± 0.089
				*P* = 0.319		*P* = 0.239		*P* = 0.988		*P* = 0.11	
				versus A+T+		versus A+T+		versus A+T+		versus **A+T+**	
				*P* = 0.319		*P* = 0.33		*P* = 0.988		***P*** **=** **0.018**	
				versus A−T−&Control		versus A−T−&CI		versus A−T−&CI		versus A−T−&Control	
				*P* = 0.319		*P* = 0.207		*P* = 0.988		*P* = 0.161	
A+T− (*N* = 25)	0.656	1.738	0.617	versus A+T+	0.782 ± 0.123	versus A+T+	0.618 ± 0.136	versus A+T+	17.23 ± 7.5	versus A+T+	0.355 ± 0.078
				*P* = 0.978		*P* = 0.712		*P* = 0.988		*P* = 0.11	
				versus A−T−&CI		versus A−T−&CI		versus A−T−&CI		versus A−T−&CI	
				*P* = 0.982		*P* = 0.712		*P* = 0.988		*P* = 0.795	
A+T+ (*N* = 121)	0.658	1.726	0.602	versus A−T−&CI	0.77 ± 0.142	versus A−T−&CI	0.602 ± 0.148	versus A−T−&CI	17.23 ± 7.34	versus A−T−&CI	0.326 ± 0.08
				*P* = 0.982		*P* = 0.712		*P* = 0.988		*P* = 0.11	
A−T−&CI (*N* = 100)	0.664	1.691	0.658		0.798 ± 0.061		0.608 ± 0.11		17.23 ± 5.59		0.359 ± 0.065

The network analysis revealed differences in SCN metrics among groups. Notably, the control group exhibited statistically significantly higher small-worldness than the group with full Alzheimer’s disease pathology, indicating more efficient information transfer both locally and across the network. Results for all group comparisons (i.e. nodal degree, nodal clustering coefficients, nodal efficiency and small-worldness) were corrected for multiple comparisons using false discovery rate (FDR) correction. Significant *P*-values were bolded. A+T−, amyloid positive, tau negative; A+T+, amyloid positive, tau positive; A−T−&CI, amyloid negative, tau negative and cognitive impaired; SD, standard deviation.

The global clustering of brain volumes by CSF Alzheimer’s disease biomarker group (visualized using UMAP in Fig. 2C) revealed a higher SGCC (*SGCC* = 0.09) at the whole brain level compared with specific subregions, such as subcortical grey matter (*SGCC* = 0.075), parietal lobe (*SGCC* = 0.044) and temporal lobe (*SGCC* = 0.026).

### Amyloid pathology associated with subcortical, cerebellar and brainstem atrophy

**Figure 3:**
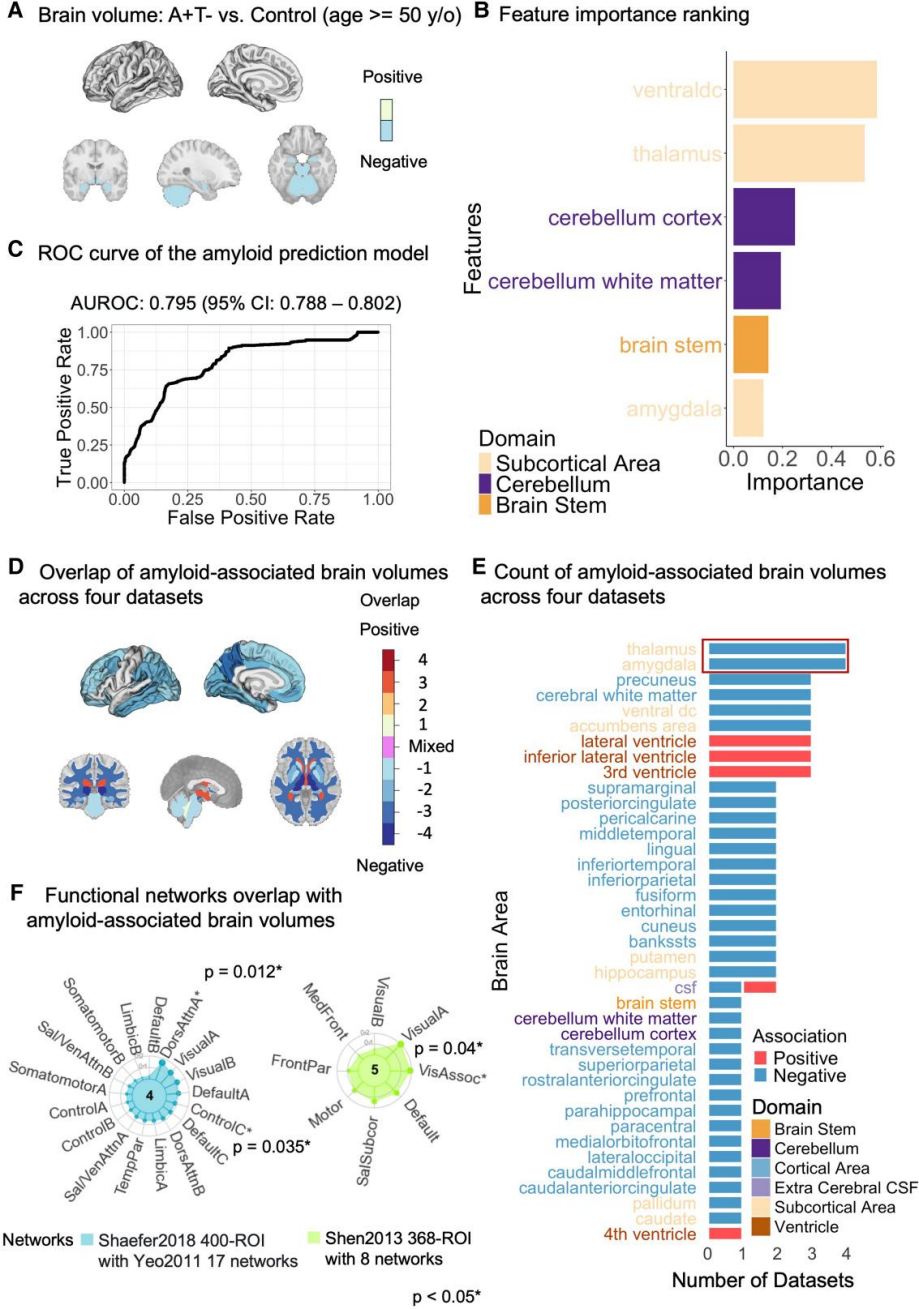
**Brain volumes associated with amyloid.** (**A**) Brain areas associated with amyloid status. Significant brain volumes linked to amyloid status using logistic regression in individuals aged 50+ in the MGB dataset (*N*: A+T− = 25, Control = 60), adjusted for age, sex and intracranial volume (ICV). Beta (log odds): brain stem = −0.512, *P* = 0.043; thalamus = −0.650, *P* = 0.03; amygdala = −0.681, *P* = 0.017; ventral DC = −0.857, *P* = 0.02; cerebellum cortex = −0.937, *P* = 0.011; cerebellum white matter = −1.200, *P* = 0.005. y/o, year old. (**B**) Feature importance ranking. Top predictors of amyloid status in the ridge logistic regression model for patients aged 50+. The *x*-axis (Importance) indicates the magnitude of each feature’s standardized coefficient, with features scaled before model fitting and importance values scaled from 0 to 1 for visualization. (**C**) Model performance. *AUROC* of the ridge logistic regression model for predicting amyloid status in patients aged 50+ (*N*: A+T− = 19, Control = 45; *AUROC* = 0.795, 95% *CI*: [0.788, 0.802], *sensitivity* = 0.67 at *specificity* = 0.87). AUROC, area under receiving operating characteristic; CI, confidence interval. (**D**) Brain visualization. Overlay of significant brain areas from logistic regression tests across datasets for individuals aged 50+. Darker colours indicate greater overlap (numbers show dataset counts per region); purple indicates mixed associations (positive in some datasets, negative in others). (**E**) Significant brain areas in logistic regression tests by dataset. A stacked bar plot displaying the count of brain areas showing significant associations across all four datasets, coloured by the association direction (positive/negative). The red box highlights the two brain areas that were significant in all datasets, indicating consistent associations. (**F**) Functional network overlap. Spin tests of significant brain regions from meta-analyses and functional networks revealed significant overlaps with control C (Dice = 0.08, *P* = 0.035), dorsal attention A (Dice = 0.16, *P* = 0.012) and visual associated networks (Dice = 0.19, *P* = 0.04), indicating consistent amyloid-associated brain volume patterns. ROI, region of interest. Network abbreviations correspond to the functional networks: DorsAttnA/B, dorsal attention network A/B; VisualA/B, visual network A/B; DefaultA/B, default mode network A/B; ControlA/B/C, frontoparietal control network A/B/C; SalVenAttnA/B, salience/ventral attention network A/B; SomatomotorA/B, somatomotor network A/B; LimbicA/B, limbic network A/B; TempPar, temporoparietal network; VisAssoc, visual association network; Default, default mode network (general); SalSubcor, salience/subcortical network; Motor, motor network; FrontPar, frontal-parietal network; MedFront, medial frontal network; InsuB, insular/brainstem network. Asterisks (*) indicate networks with significant overlap. The *P*-value was based on the spin test permutations of the Dice coefficients. (**A**) and (**D**) show sagittal views of cortical areas (left) and coronal, sagittal and sectional views of subcortical and white matter areas (right). A+T−, amyloid positive, tau negative.

For amyloid status in the MGB dataset, we identified six significantly associated brain regions (unadjusted *P* < 0.05), including subcortical areas (e.g. amygdala, thalamus, ventral diencephalon), the cerebellum (cerebellar white matter and cortex) and the brainstem (Fig. 3A, Supplementary Fig. 2). Using these significant features to predict amyloid status, a ridge logistic regression model achieved the best performance with an *AUROC* of 0.795 (95% *CI*: [0.788, 0.802]) and a sensitivity of 0.67 at specificity of 0.87. The ventral diencephalon, thalamus and cerebellum cortex emerged as the features with the highest predictive power (Fig. 3B and C).

Validation across three independent datasets partially confirmed the findings in the discovery sample (MGB). The amygdala and thalamus demonstrated significance across all four datasets (Fig. 3D and E). Next, we performed meta-analysis on brain regions with significant associations with amyloid in at least one dataset. Meta-analyses conducted across four datasets confirmed significant associations in 14 brain areas (Supplementary Fig. 3 and Table 2). Dice coefficient tests were then performed on the amyloid-associated regions identified from the meta-analyses, comparing them with functional networks from commonly used atlases. This analysis revealed significant overlap with dorsal attention A, control C and visual association networks (Fig. 3F, Supplementary Fig. 4), which are key functional systems involved in attention regulation, executive control and visual processing, respectively.

### Tau pathology in the presence of amyloid associated with extracerebral CSF enlargement and unexpected ventricular shrinkage

**Figure 4:**
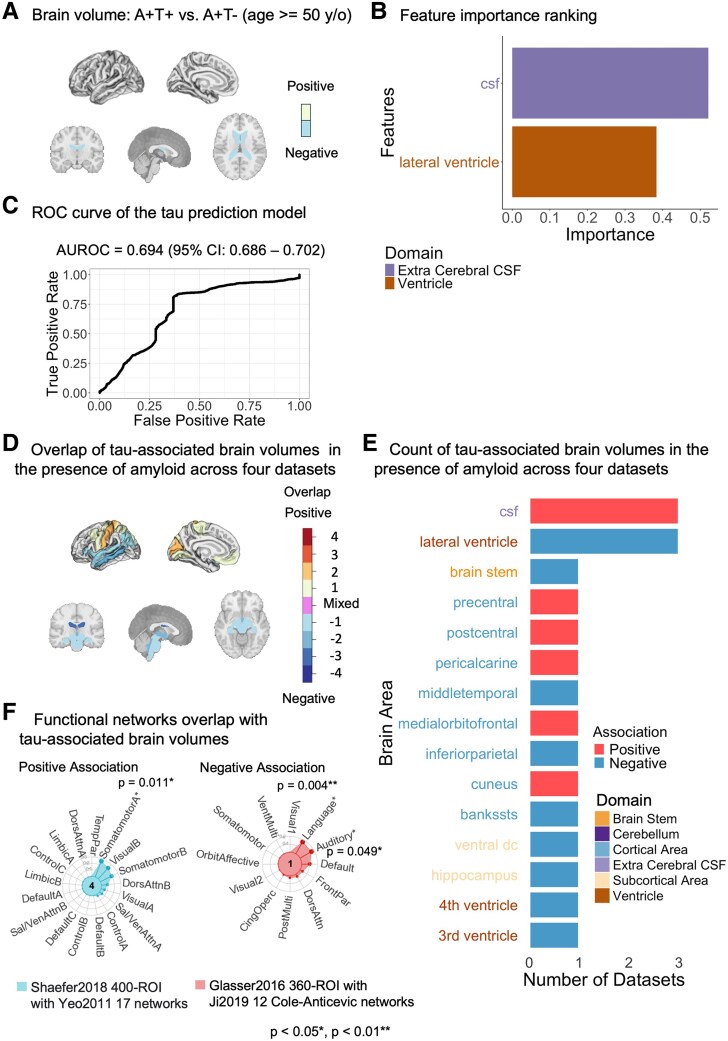
**Brain volumes associated with tau in the presence of amyloid.** (**A**) Brain areas associated with tau status. Significant brain volumes linked to tau status using logistic regression in amyloid-positive individuals aged 50+ in the MGB dataset (*N*: A+T+ = 121, A+T− = 25), adjusted for age, sex and intracranial volume (ICV). Beta (log odds): CSF = 0.668, *P* = 0.006; lateral ventricle = −0.545, *P* = 0.009. y/o, year old. (**B**) Feature importance ranking. Top predictors of tau status in the ridge logistic regression model for patients aged 50+. The *x*-axis (Importance) indicates the magnitude of each feature’s standardized coefficient, with features *z*-scored before model fitting and importance values scaled from 0 to 1 for visualization. (**C**) Model performance. AUROC of the random forest model for predicting amyloid status in patients aged 50+ (*N*: A+T−=19, A+T+=91; *AUROC* = 0.694, 95% *CI*: [0.686, 0.702], *sensitivity* = 0.73 at *specificity* = 0.5). ROC, receiving operating characteristic; AUROC, area under receiving operating characteristic; CI, confidence interval. (**D**) Brain visualization. Overlay of significant brain areas from logistic regression tests across datasets for individuals aged 50+. Darker colours indicate greater overlap (numbers show dataset counts per region); purple indicates mixed associations (positive in some datasets, negative in others). (**E**) Significant brain areas logistic regression tests by dataset. A stacked bar plot displaying the count of brain areas showing significant associations across all four datasets, coloured by the association direction (positive/negative). (**F**) Functional network overlap. Spin tests of significant brain regions from meta-analyses and functional networks revealed significant overlaps of tau-associated brain volumes in the presence of amyloid revealed with somatomotor A (increased volumes; Dice = 0.26, *P* = 0.011) and language (decreased volumes; Dice = 0.17, *P* = 0.004) and auditory networks (decreased volumes; Dice = 0.17, *P* = 0.049). ROI, region of interest. Network abbreviations correspond to the functional networks: SomatomotorA/B, somatomotor network A/B; VisualA/B/2, visual network A/B/2; DorsAttn, dorsal attention network; Default, default mode network; ControlA/B/C, frontoparietal control network A/B/C; SalVenAttnA/B, salience/ventral attention network A/B; LimbicA/B, limbic network A/B; TempPar, temporoparietal network; FrontPar, frontal-parietal network; VentMulti, ventral multimodal network; PostMulti, posterior multimodal network; CingOperc, cingulo-opercular network; OrbitAffective, orbitofrontal/affective network; Auditory, auditory network; Language, language network. Asterisks (*) indicate networks with significant overlap. The *P-*value was based on the spin test permutations of the Dice coefficients. (**A**) and (**D**) show sagittal views of cortical areas (left) and coronal, sagittal and sectional views of subcortical and white matter areas (right). A+T−, amyloid positive, tau negative; A+T+, amyloid positive, tau positive.

For tau status in the presence of amyloid, we only identified two significant features, which were lateral ventricle and extracerebral CSF (Fig. 4A, Supplementary Fig. 5). Ridge logistic regression model yielded the best performance utilizing these significant features for predicting tau status in the presence of amyloid, with an *AUROC* of 0.694 (95% *CI*: 0.686–0.702) and a sensitivity of 0.73 at specificity of 0.5. Extracerebral CSF showed higher predictive power than lateral ventricle in terms of feature importance ranking (Fig. 4B and C).

Validation across three independent datasets confirmed the findings in the MGB discovery sample; extracerebral CSF and the lateral ventricle were significant in three datasets (Fig. 4D and E). This observed overlap informed the selection of brain regions for meta-analyses, focusing on those with significant associations with tau in at least one dataset. Next, we performed meta-analysis on brain regions with significant associations with p-tau in at least one dataset (Supplementary Fig. 6 and Table 3). Dice coefficient tests were then applied to these regions, comparing them with functional networks from commonly used atlases. This analysis revealed significant overlaps: increased brain volumes were associated with the somatomotor networks (Fig. 4F, Supplementary Fig. 7), while decreased brain volumes were associated with language and auditory networks (Fig. 4F, Supplementary Fig. 8).

### Sex differences in brain volumes across CSF Alzheimer’s disease biomarker groups

**Figure 5:**
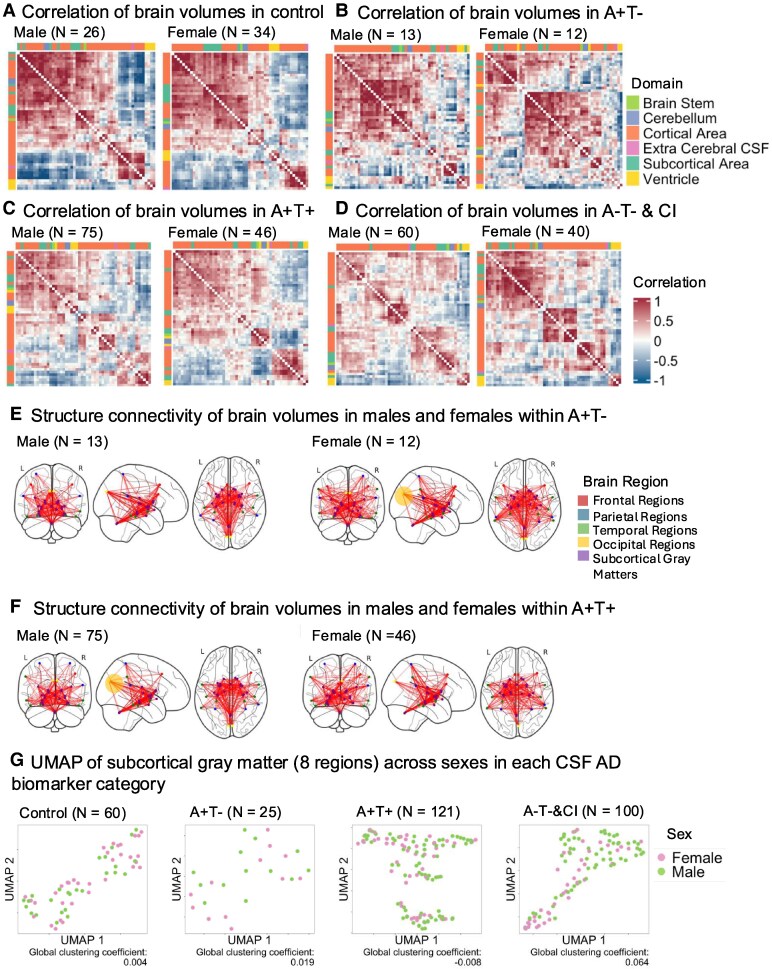
**Sex differences in neurodegeneration patterns across CSF Alzheimer’s disease biomarker groups in the MGB dataset.** (**A–D**) Brain volume correlations by sex: partial bivariate Pearson correlations of brain volumes in males and females within each CSF biomarker group: (**A**) Control group (*N* = 60), (**B**) A+T− group (*N* = 25), (**C**) A+T+ (*N* = 121) group and (**D**) the A−T−&CI group (*N* = 100). Different connectivity patterns were observed between sexes across all groups. Brain regions are colour-coded (subcortical, cortical, cerebellum, ventricle, brain stem and extracerebral CSF). Correlations are adjusted for age and Fisher transformed. (**E** and **F**) SCNs in A+T+ group: Visualization of the top 10% strongest connections in males and females. Connection patterns were based on visual inspection only; no statistical comparisons were conducted for regional edge differences. (**E**) Female-specific patterns: More connections between the occipital and parietal lobes (yellow circle). (**F**) Male-specific patterns: More connections between the occipital lobe and subcortical grey matter (yellow circle). (**G**) Subcortical grey matter clustering by sex. UMAP visualization shows sex-based clustering of subcortical grey matter volumes in control, A+T− and A−T−&CI groups, but not in the A+T+ group. A+T−, amyloid positive, tau negative; A+T+, amyloid positive tau positive; A−T−&CI, amyloid negative, tau negative and cognitive impaired; UMAP, uniform manifold approximation and projection; MGB, Mass General Brigham; L, left hemisphere; R, right hemisphere.

To examine neurodegeneration patterns between sexes across CSF Alzheimer’s disease biomarker groups, we first conducted sex-stratified partial bivariate Pearson correlations of brain volumes for each group, adjusting for age (Fig. 5A–D). Sex differences in brain volume clustering patterns were clear in the control, A+T− and A−T−&CI groups (Fig. 5A, B and D), while the A+T+ group revealed less prominent sex difference (Fig. 5C).

Next, SCN analyses revealed no significant sex difference in any groups (*Ps* > 0.005; Supplementary Table 4) although network visualization exhibited stronger connections near occipital lobe in female A+T− and male A+T+ groups (Fig. 5E and F).

UMAP visualization (Fig. 5G) of global brain volume clustering by sex across CSF Alzheimer’s disease biomarker groups revealed sex-based clustering in subcortical grey matter for control (*SGCC* = 0.004), A+T− (*SGCC* = 0.019) and A−T−&CI (*SGCC* = 0.064). Additionally, sex clustering was observed at the whole brain level in control, A+T+ and A−T−&CI in the parietal for control and A−T−&CI groups, in the temporal lobe for all groups and in the occipital lobe for A+T− and A+T+ groups (Supplementary Fig. 9). No sex clustering was detected in the frontal lobe.

**Figure 6:**
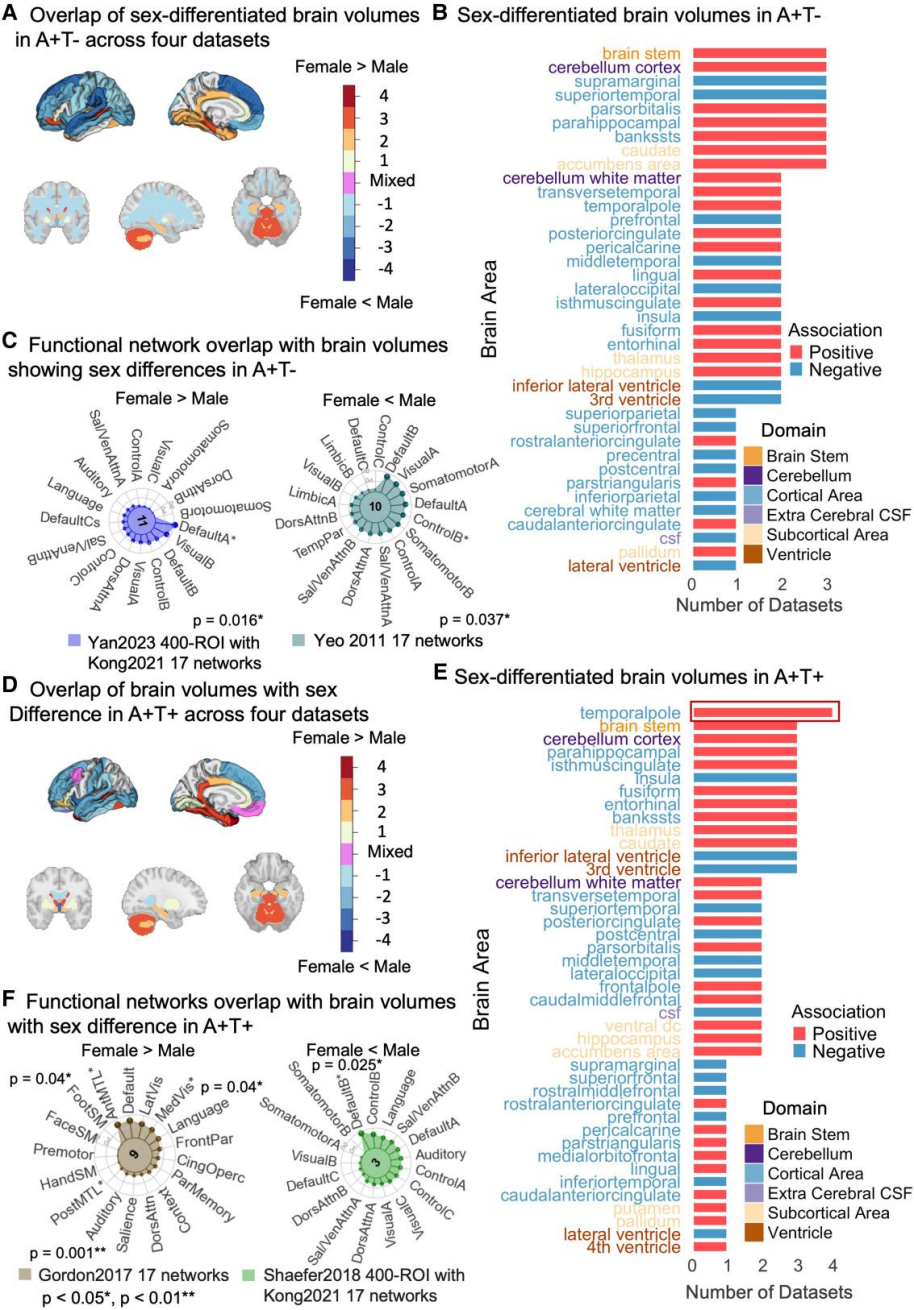
**Sex-differentiated brain regions in A+T− and A+T+ groups.** (**A**) Overlap of sex-differentiated brain volumes in A+T−. Visualization of overlapping regions across four datasets. A+T−, amyloid positive, tau negative. (**B**) Count of sex-differentiated brain volumes in A+T−. Stacked bar plot showing the count of significant brain volumes in individuals aged 50+, coloured by association direction (Female > Male or Male > Female). (**C**) Functional network overlap in A+T−. Spin tests of significant brain regions from meta-analyses and functional networks revealed significant overlap with default A network (larger brain volumes in females; Dice = 0.21, *P* = 0.016) and control B network (larger brain volumes in males; Dice = 0.13, *P* = 0.037). (**D**) Overlap of sex-differentiated brain volumes in A+T+. Visualization of overlapping regions across four datasets. (**E**) Count of sex-differentiated brain volumes in A+T+. Stacked bar plot showing significant regions for individuals aged 50+, with colours indicating association direction. The red box highlights the consistently significant brain area (temporal pole) across all datasets. A+T+, amyloid positive, tau positive. (**F**) Functional network overlap in A+T+. Spin tests of significant brain regions from meta-analyses and functional networks revealed overlap with anterior medial temporal lobe (larger brain volumes in females; Dice = 0.16, *P* = 0.04), posterior medial temporal lobe (larger brain volumes in females; Dice = 0.019, *P* = 0.001) and medial visual networks (larger brain volumes in females; Dice = 0.012, *P* = 0.044) and default B network (larger brain volumes in males; Dice = 0.017, *P* = 0.025). Network abbreviations correspond to the functional networks: DefaultA/B/C, default mode network A/B/C; ControlA/B/C, frontoparietal control network A/B/C; VisualA/B/2, visual network A/B/2; DorsAttnA/B, dorsal attention network A/B; SalVenAttnA/B, salience/ventral attention network A/B; SomatomotorA/B, somatomotor network A/B; LimbicA/B, limbic network A/B; TempPar, temporoparietal network; Language, language network; Auditory, auditory network; VentMulti, ventral multimodal network; PostMulti, posterior multimodal network; VisualCs, visual central strip network; VisualCb, visual cerebellar network; CingOperc, cingulo-opercular network; OrbitAffective, orbitofrontal/affective network; MedVis, medial visual network; LatVis, lateral visual network; Context, contextual association network; ParMemory, parietal memory network; FrontPar, frontal-parietal network; Premotor, premotor network; PostMTL, posterior medial temporal lobe network; TLMN, temporal lobe midline network; FootSM, HandSM, FaceSM, somatomotor subregions (foot, hand, face). Asterisks indicate networks with significant overlap. The *P-*value was based on the spin test permutations of the Dice coefficients. In (**A**) and (**D**), darker colours indicate greater overlap (numbers show dataset counts per region); purple indicates mixed associations (positive in some datasets, negative in others).

Sex-differentiated brain volumes were observed across CSF Alzheimer’s disease biomarker groups. In the A+T− group, females had larger volumes overlapping with the default mode network A (involved in autobiographical and prospective memory^36^), while males exhibited larger volumes in the frontal and parietal lobes, overlapping with the control network B (associated with executive functions and cognitive control) (Fig. 6A–C, Supplementary Fig. 10 and Table 5). In the A+T+ group, females showed larger volumes in temporal and visual networks (involved in memory and visual processing), while males had overlaps with the default mode network B (linked to Theory of Mind^36,37^) (Fig. 6D–F, Supplementary Fig. 11 and Table 6). Additional analyses across the other dementia and control groups showed sex differences, with further details provided in Supplementary Fig. 12.

Moreover, meta-analyses with multiple comparison corrections revealed a significant sex difference in the association between inferior lateral ventricle volume and amyloid status. Specifically, males in the A+T− group exhibited significantly greater enlargement of the inferior lateral ventricles compared with females when contrasted with the control group (*β* = −0.28, *pFDR* = 0.005) (Supplementary Fig. 13). However, no sex difference was observed in the association with tau in the presence of amyloid.

## Discussion

In this study, we examined neurodegeneration patterns across four CSF Alzheimer’s disease biomarker groups. We found disrupted connectivity (brain volume covariance networks) in groups with Alzheimer’s disease pathology (A+T−, amyloid positive, tau negative; A+T+, amyloid positive, tau positive) and other non-Alzheimer’s disease dementias (A−T−&CI, amyloid negative, tau negative, cognitive impairment), while the control group had the most connected structural network and exhibited significantly higher small-worldness compared with the A+T+ (amyloid positive, tau positive) group. High-dimensional clustering analysis showed that whole brain volumes demonstrated more separation between groups than subregions. We also identified associations between brain region volumes and amyloid and tau in the presence of amyloid, with subcortical, cerebellar and brainstem atrophy linked to amyloid, including the ventral diencephalon, thalamus and cerebellum cortex showing the highest predictive power. The amygdala and thalamus had consistent cross-dataset associations. For tau in the presence of amyloid, we mostly identified brain volume changes that reflect the whole brain shrinkage, with the lateral ventricle (negative, unexpected) and extracerebral CSF (positive) being the most predictive. High overlap was found across datasets in these two regions. Finally, we revealed distinct sex-based variations in brain volumes in all biomarker groups but no significant difference in connectivity (brain volume covariance networks) across any group. High-dimensional analysis also identified distinct sex-based clustering patterns.

This study builds upon previous research by leveraging a large dataset from the MGB healthcare system, validated with three public datasets, totalling 3443 participants. This sample size is significantly larger than prior studies, which often focused on a smaller number of brain regions such as the hippocampus,^16-18^ and included fewer participants^16-19,24,25,38-40^ By assessing 52 brain subregions, we were able to capture a more comprehensive view of structural brain changes across the whole brain and across a broader spectrum of Alzheimer’s disease. We also accounted for variations in CSF assay methods across the datasets, leading to more robust and generalizable findings. We focused on core Alzheimer’s disease biomarkers,^3^ especially tau in the presence of amyloid, which, to our knowledge, has not been directly studied in relation to CSF Alzheimer’s disease biomarkers and brain volume.

Our results highlight significant disruptions in brain network connectivity (brain volume covariance networks) associated with Alzheimer’s disease pathology and other dementias, with the control group showing the most connected network. This higher small-worldness in controls may support cognitive resilience and preserved brain function during aging. The greater differences observed in whole brain volumes across the four groups compared with subregions suggest widespread structural changes, rather than localized effects, across the Alzheimer’s disease CSF biomarker groups. Our study also emphasized the importance of examining global structural connectivity when assessing dementia.

Further, our study contributes to resolving discrepancies in prior research concerning the relationship between CSF Alzheimer’s disease biomarkers and brain volume.^16-19^ We identified consistent amyloid-related structural changes, such as those in the thalamus and amygdala, areas known to be affected early by amyloid accumulation.^41-44^ Despite the small sample size of 25 participants in the A+T− group within the discovery set, our three independent validation sets consistently supported these findings. Regarding the accumbens, while amyloid-associated atrophy has not been directly reported, it may result from cholinergic neuronal loss in the basal forebrain, which is closely linked to amyloid accumulation through the cholinergic pathway.^45^ Thalamus atrophy has also been observed in Alzheimer’s disease,^46^ which may be part of this broader neurodegenerative pattern. Additionally, brain regions showing greater variability across datasets may reflect patterns specific to each dataset. For instance, cerebellum and brainstem atrophy were associated with amyloid only in the MGB dataset but have been reported in post-mortem studies.^47-49^ The MGB dataset includes patients at more advanced disease stages compared with other datasets like ADNI and EPAD, suggesting that cohort characteristics, such as disease severity, may influence the observed associations. Furthermore, regions showing significant volume reduction in amyloid-positive individuals overlap with multiple functional networks. This is supported by cognitive tasks highly associated with amyloid status, such as picture sequence memory (involving the DMN, control and visual networks)^50^ and list sorting working memory (engaging control and attention networks).^50^

In contrast, we primarily observed general shrinkage of the whole brain (i.e. increase of extracerebral CSF^51,52^) for tau in the presence of amyloid. Meta-analyses revealed that there was an increase in volumes in the occipital and parietal regions and a decrease in ventricular size associated with tau in the presence of amyloid. The observed ventricular shrinkage is particularly puzzling, as it contradicts the expected ventricular dilation typically associated with neurodegeneration. This counterintuitive relationship may reflect heterogeneity in disease stages and/or the presence of mixed pathologies. Additionally, accurately estimating brain volumes from T_1_-weighted MRIs may present challenges in cases of significant neurodegeneration. As brain tissue shrinks and CSF volume increases, partial volume effects can occur—where individual voxels contain a mixture of CSF and atrophied brain tissue—thereby reducing contrast and complicating precise measurements. Moreover, severe neurodegeneration and elevated CSF volumes may dilute protein concentrations, potentially skewing biomarker values. Interestingly, the volume increases we observed in occipital regions (A+T+ versus A+T−, adjusted for intracranial brain volume), such as the cuneus, pericalcarine and lingual gyrus, are consistent with previous studies reporting enhanced functional connectivity in these areas among Alzheimer’s disease patients.^53,54^ We performed visual inspections of multiple MRIs to confirm the automated image segmentations, but the observed decrease in ventricle size remained. These results warrant further investigation into the complex interactions between tau, amyloid and brain structure.

Our findings on sex differences in brain volumes revealed complex but distinct patterns across CSF Alzheimer’s disease biomarkers. Interestingly, the A+T+ group appears to have less prominent sex differences than other groups, which indicates that sex differences in brain atrophy may vary across different stages of Alzheimer’s disease. In the A+T− group, we observed sex-based clustering in the subcortical grey matter, temporal and occipital lobes, with males showing larger volumes in regions (e.g. middle and superior temporal gyri, supramarginal gyrus) linked to executive function (i.e. control B subnetwork^55^), while females had larger volumes in areas related to autobiographical and prospective memory^36^ (DMN A subnetwork,^56^ e.g. temporal pole, parahippocampal areas, posterior cingulate cortex). These results align with previous findings that females may have greater cognitive reserve, though experiencing more rapid cognitive decline^57^ including executive function,^27,58^ particularly at higher amyloid levels. In the A+T+ group, we observed sex-based clustering in occipital areas and at the whole brain level, with females exhibiting larger volumes in the temporal pole cross-datasets. Further, our meta-analyses revealed that sex differences in the enlargement of inferior lateral ventricles are particularly associated with amyloid, in line with previous findings that ventricular enlargement is more associated with CSF amyloid than p-tau.^20,21,23^ It is important to note that multiple comparison corrections were applied to the meta-analyses to reduce the risk of false positives. Additionally, recent findings suggest that CSF glial reactivity may also be related to sex differences in preclinical Alzheimer’s disease groups.^59^ Specifically, women showed increased amyloid burden and CSF p-tau levels with elevated CSF glial markers, while men with higher tau burden exhibited lower hippocampal volumes with increased CSF glial reactivity. These results indicate that CSF glial reactivity may help explain some of the variations in the relationships between CSF Alzheimer’s disease biomarkers and brain structure observed across datasets, suggesting a valuable direction for future research.

## Limitations

This study has several limitations. First, the slice thickness of clinical images posed challenges for calculating other morphometric measures, particularly cortical thickness. Previous studies have shown that tau is more strongly associated with cortical thinning than hippocampal volume, while the reverse is true for amyloid in preclinical Alzheimer’s disease.^39^ Thus, it is possible that tau in the presence of amyloid may exhibit more consistent and widespread associations with cortical thickness than amyloid alone. Second, we employed a cross-sectional design to examine the association between brain volume and CSF Alzheimer’s disease biomarkers. Future longitudinal studies will be necessary to fully track how brain volume changes with these biomarkers over time. Third, the average education level of our participants was higher than that of the general population, which may limit the generalizability of our findings to less educated populations. This potential bias should be considered when interpreting our results. Lastly, hormone therapy, known to affect the volumes of several brain regions including the hippocampus and frontal lobe,^60^ was not consistently recorded across all datasets.

## Conclusions

In this comprehensive study of neurodegeneration patterns across CSF Alzheimer’s disease biomarker groups, we leveraged a dataset from the MGB healthcare system, validated with three public datasets, totalling 3443 participants. Our findings revealed disrupted connectivity in groups with Alzheimer’s disease pathology and other dementias, contrasting with the well-connected networks in the control group. Whole brain volumes showed greater differences between groups than subregions, emphasizing the importance of global structural analysis in the assessment of neurodegenerative patterns. We identified consistent amyloid-related structural changes in amygdala and thalamus, while tau in the presence of amyloid showed extracerebral CSF enlargement and unexpected ventricular shrinkage. There were pronounced differences between sexes in brain volumes within each CSF Alzheimer’s disease biomarker group, but no significant differences were observed in connectivity between sexes. These findings enhance our understanding of Alzheimer’s disease neurodegeneration patterns and demonstrate the effectiveness of automated analyses on real-world datasets.

## Supplementary Material

fcaf210_Supplementary_Data

## Data Availability

Data from the MGB (Mass General Brigham) healthcare system cannot be shared due to privacy concerns. For NACC (National Alzheimer’s Coordinating Center), ADNI (Alzheimer’s Disease Neuroimaging Initiative) and EPAD (European Prevent of Alzheimer’s Dementia), access to data is available through a formal application and review process to maintain participant confidentiality. Details on how to apply can be found at the following links: NACC’s data request page (https://naccdata.org/), ADNI’s data request page (https://ida.loni.usc.edu/) and EPAD’s data request platform (https://fair.addi.ad-datainitiative.org/#/data/datasets/v_imi_epadlcs). The code used for data analysis is available at https://github.com/mindds/brain-csf-mri/.

## Appendix I

Members of the European Prevention of Alzheimer’s Disease (EPAD) Consortium: Frank Tennigkeit, Andrea Pfeifer, Christine Vincenzetti, Fabien Monin, Gautam Maitra, Joseph O’Sullivan, Alex Teligladas, Ana Diaz, Christophe Bintener, Cindy Birck, Dianne Gove, Jean Georges, Katherine Ellis, Stefanie Peulen, Eva Danasi, Georgios Vlachos, Nicolaos Scarmeas, Alexandre Mencik, Cynthia Ng, Hernan J. Picard, Mats Ericson, Matt Gaunt, Nicolas Pourbaix, Rob Lenz, Rodolphe Eyraud, Ross McGarrity, Veronique Chauvet, Ala-Eddine Allouche, Béatrice Le Balanger, Bruno Dubois, Edith Benmansour, Francis Nyasse, Hicham Filali, Ihsen Youssef, Leslie Ameil, Marion Dubois, Nabila Lajaail, Nabila Nyasse, Nadja Younsi, Patrick Feukam Talla, Souza Benadjaoud, Stephane Lehericy, Tatiana Akake Beshelemu, Belén Garcia Alonso, Bet Molina, Ian Sherriff, Pedro Pesini, Pedro Tirado, Virgina Pérez, Alan McNeill, Alicia Gibson, Andrew Fox, Bert Overduin, Christine Lee, David Allan, David Taylor, Emma McDonald, Harry Peaker, Karen O’Hanlon, Karen Thornton, Lorna Harper, Pamela Brankin, Robert Bryce, Rodrigo Barnes, Ana Belén Callado, Andreea Radoi, Annabella Beteta, Arcadi Navarro, Beatriz Blasco, Carolina Herrero, Carolina Minguillon, Eider Arenaza, Eva Nebot, Gema Huesa, Gemma Salvadó Blasco, Gloria Oliver, Gonzalo Sánchez Benavides, Gregory Operto, Iva Knezevic, Jordi Camí, Jose Luis Molinuevo, Juan Domingo Gispert, Julieta Pereda, Karine Fauria, Laia Tenas, Mahnaz Shekari, Maria Teresa Buongiorno, Maria Escrivà, Marta Milà, Meritxell Santos, Monica Montserrat, Montse Alegret, Neus Cadevall, Noemí Carranza, Oriol Grau Rivera, Paula Marne, Paula Petrone, Raffaele Cacciaglia, Sofia Menezes-Cabral, Barbara Wendelberger, Mark Fitzgerald, Melanie Quintana, Scott Berry, Shawn Andersson, Tammy Berry, Andy Bolan, Bjorn Sperling, Dominic Walsh, Elena Ratti, Emily Smyth, Fred Lawson, Inna Tsvang, Jeffrey Sevigny, Joao Pinho brandao, John Ogorman, Marcus Unternärer, Marija V. Jankovich, Martin Pan, Olga Mitelman, Philip Liston-Kraft, Philipp von Rosenstiel, Samantha Buddhaeberlein, Sean Knox, Tina Olsson, Birgit Goerke, Catherine Debove, Daniela Harder, Dorothee Schwall-rudolph, Gine Plaue, Glen Wunderlich, Jasmin Ghazvini, Julia Meyer-Kleinmann, Kathrin Huber, Lisa Hattemer, Mark Gordon, Martina Eckes, Mary Potter, Melanie Nusser, Miguel Garcia, Pamela Drake, Pauric Mcginty, Rolf Goeggel, Russel Jones, Sabine Rau, Shannon Scanlon, Stephane Pollentier, Stephanie Sommer, Susanna Flaherty, Wilhelm Dieminger, Daniela Savina, Bernard Hanseeuw, Murielle Bortolotti, Jean-Francois Demonet, Warszawa, Marta Biel-Czarnecka, Maciej Czarnecki, Enise I. Incesoy, Felix Menne, Katja Lindner, Oliver Peters, Melanie Leroy, Florence Pasquier, Manon Laforce, Audrey Gabelle, Caroline Grasselli, Charlotte Leger, Aurelie Gillet, Christelle Cadiet, Claire Boutoleau, Manon Guiguen, Rachel Chaigneau, Romain Muraz, Samia Melcion, Tiphaine Charriau, Béatrice Dinthilac, Brune Rieunier, Bruno Vellas, Camille Coulange, Delphine Pennetier, Isabelle Carrie, Julien Delrieu Lapeyre, Laure Saint-Aubert, Natalia del Campo, Nicole Batonneau, Pierre Jean Ousset, Sandrine S.A. Andrieu, Sophie Mourgues, Genoveva Montoya, Mario Riverol, Virginia Blasco, Amy Gerrish, Elisa Majouni, Felicity Sheppard, Georgina Menzies, Harriet Oldham, Julie Williams, Keith Sexton, Rebecca Sims, Sian Jones, Steven Head, Teresa Bowen, Thomas Cushion, Valentina Scott-Price, Gael Chetelat, Ami Saver, Bruce Albala, Danielle Fry, Eve Potter, Johan Luthman, Jude Concepcion, Lan Bandara, Lynn Kramer, Martin Rabe, Michele Valeri, Nadeem Sarwar, Robert Gordon, Sharon Dispoto, Shilpa Kakad, Shobha Dhadda, Sindhuja Musku, Sonjoy Braham, Sue Lyons, Susan DevenishMeares, Alette Wessels, Andrew G Smith, Birgit Steckel-hamann, Chrissie Spears, Dan Holding, Eric Siemers, Florence Bayard, Holger Zimmermann, Jayetta Embry, Jennie Lenora Walgren, Jennifer Zimmer, Michael Hutton, Michael C. Irizarry, Trevor Smart, Alexander de Jong, Cornelia van Duijn, Eline Bunnik, Johan van der Lei, Krista Tromp, Laurence Hoezen, Maartje Schermer, Ramona van Dijk, Sander Woerdeman, Fabian Perpeet, Hannah Wolff, Marc Zimmermann, Martin Hofmann-Apitius, Meemansa Sood, Tobias Rechmann, Ana Pancho, Charo Cuevas, Mar Buendia, Merce Boada, Amaya García-Eizaga, Mikel Tainta, Pablo Martinez-Lage, Jon Saldias, Marta Canada, Ben Newton, Christopher Foley, Anita Davies, Heena Mistry, Iracema Leroi, Rachel Rosenhead, Ross Dune, Rowen Norton, Tobias Langheinrich, Adrià Tort, Beatriz Bosch, Guadalupe Fernandez, Mircea Balasa, Andrea Fernandez, Jesus Pascual Sanchez, Sara Lopez, Marta Ferrando, Rocío García, Aixa Ghastin, Rafael Arroyo, Alicia Muñoz, Angeles Barro, Féix Viñuela Fernández, Tom Ashby, Tom Haber, Michael Ropacki, Alessandra Cincotti, Aurélie Gauthier, Aurélie Nier, Charlotte Dumonte, Florent Trecanne, Isabelle Carriere, Isabelle Geahel, Karen Ritchie, Marie Baquet, Marion Mortamais, Vincent Boyer, Andrew Satlin, Alasdair MacDonald, Andrea Morton-Morys, Andrew Danson, Anouska Penny, Ben Cons, Benjamin Spence, Betsy Cooke, Catherine Brooker, Cathy Vanbelle, Derenda West, Elisabeth, Gilmour, Emma Parkinson, Fiona Ramage, Gary Johnson, James Rattenbury, Janet Hall, Jennifer Chambers, John Tracey, Joseph Milne, Lynne Hughes, Martin Dvorak, Rebecca Nagle, Salwa Beydoun, Shari Scott, Siva Kumar, Zia Haque, Alberto Redolfi, Anna Mega, Daniele Altomare, Elena Rolandi, Michela Rampini, Moira Marizzoni, Sara Gipponi, Valentina Nicolosi, Valentina Saletti, Samanta Galluzzi, Conor Wilson, Derek Hill, Elin Rees, Heather Welch, Ioana Ababei, Jane Whitrow, Janet Munro, John Hall, John Woodside, Kate Barnes, Kate McLeish, Lea Marai, Maartje Meijer, Mark Austin, Maryam Abaei, Mauro Sousa, Michelle Lax, Mike Radford, Oleg Belov, Rebeca James, Robin Wolz, Susan Lowther, Abbi Gower, Aline Smits, Ann Dierckx, Bart Vannieuwenhuyse, Caroline Sage, Christine Cully, Christophe Verbruggen, David Symnoski, Derya Ayaz, Enchi Liu, Eric Crouthamel, Gary Romano, Gerald Novak, Hendrik Sipma, Howard Greenberg, Isabelle Coste, Jeroen Schuermans, Jerry Novak, John Kemp, Joris Beke, Karen Bachner, Katrin Haeverans, Kyle Wathen, Laura Carrera, Lennert Steukers, Liesbeth Cluckers, Liesbeth Wouters, Lisa Ford, Luc Truyen, Lynn Yieh, Magda Van Dyck, Mahesh Samtani, Marie de Vulder, Marija Jovanovic, Marisa Ehinger, Mark Schmidt, Melanie Warzeski, Michael Fogle, Michele Mangini, Mila Etropolski, Nikolay Manyakov, Serge Van Der Geyten, Seth Ness, Simon Lovestone, Sipma Hendrik, Susan Katz, Suzanne Foy, Theresa Martino, Tim Elliott, Timothy, Tracy, Vanessa Bosmans, Vladimir Dragalin, Wouter Deneyer, Yordan Godinov, Alina Solomon, Ann-Marie Stromberg, Applee Akter, Björn Kull, Francesca Mangialasche, Göran Hagman, Gunhild Waldemar, Gunilla Johansson, Hilkka Soininen, Ida Kettley, Katarina Risbecker, Malin Aspö, Marie Larksater, Myroslava Protsiv, Nenad Bogdanovic, Phatcharee Chareonto, Shireen Sindi, Steen Hasselbalch, Stefan Borg, Ulrika Akenine, Vesna Jelic, Miia Kivipelto, Jessica Röber, Robert Perneczky, Bjørn Grønning, Corine Baayen, Ejner Knud Moltzen, Helle Birgitte Mengel, Henrik Bolgan, Thomsen, June Lund Pedersen, Kristian Windfeld, Lisa Hellström, Märta Segerdahl Storck, Morten Rosted, Philip Hougaard, Tilde Dyring Weidlich, Carine Djuika, Lutz Frölich, Agnieszka Trubacz, Christopher Weber, Christopher Randolph, Harald Hampel, Michael Ewers, John Harrison, Andrew Donaldson, Donna Cairney, Sheila MacFayden, Tracy Baird, Adrian Mander, Andrea Wadeson, Brian Tom, Costanza Di Fant, Gavin Lingiah, James Howlett, Keith Merle, Lisa Brown, Rebekah Davies, Robin, Huang, Simon White, Steven Hill, Farid Chekani, Jina Swartz, Letitia Reyniers, Rezaul Khandker, Tamara Connelly, Laurie Ryan, Elizabeth Coulthard, James Selwood, Natalie Rosewell, Rebecca Cousins, Cynthia Duggan, Catriona McNeill, Derek Brown, Shona McKay, Alasdair Lawrie, Emma Darling, Julie Scott, Kirsten McClelland-Brooks, Justine Hudson, Kate Ferguson, Peter Connelly, Prasad Guntur Ramkumar, Tiffany Stewart, Ana Graf, Barbara Stolz, Dieter Nussher, Etienne Pigeolet, Giulia Lestini, Gwenaelle Fillon, Ines Paule, Marianne Maman, Sema Cheyun, Stephan Korte, Yvonne Madawela, Jeff Kaye, Bruno Dubois, Patrick Feukam Talla, Sana Akel, Baptiste Porte, Julien Dumurgier, Sandrine Indart, Yaël Slama, Alexandra Fayel, Gerald Luscan, Hannah Hurst, James Eshelby, Mariana Gameiro, Max Mirza, Olga Krylova, Olivier Drap, Paul Marrone, Rachel Schindler, Yves Brault, Ewa Iwaniuk, Magdalena Lapinska, Magdalena Maslowska, Jacek Dobryniewski, Michael Gold, M.A.-LEK-Maciej Maciejowski, Christina Rabe, Enisa Alibasic, Friedemann Krause, Jason Hanon, Joanna Wright, Jyoti Srivastava, Maryline Simon, Richard Batrla-Utermann, Silvia Barbara Schmid, Simone Wahl, Udo Eichenlaub, Verena Neuhold, Ingo Kilimann, Stefan Teipel, Clemens Peter, Dianne Stephanie Van Houdt, Edo Richard, Jordy Birahy, Marthe Smedinga, Reli Pascale, Sonja Bemelmans, Stephanie Beukers, Wim van Oijen Samodzielny Publiczny Szpital Kliniczny, Katarzyna Lucka, Konrad Rejdak, Aleksandra Stjepanovic-Agovic, Caroline Cohen, Cornelia Mockwitz, Frederic Delalonde, Isabelle Clavier, Jean-François Dedieu, Laurence Mazuranok, Nacera Hamdani, Nathalie Piton, Philippe Rocolle, Romain Hahn, Carlos Díaz, Estefanía Callado, Nina Coll, Nuria Subirats, Saira Ramasastry, Sandra Pla, Achim Fischer, Adam Schwarz, Alex Bowtell, Antonella Chiucchiuini, Henrik Andersson, Melissa Naylor, Polyna Khudyakov, Rajan Perinpanayagam, Wei Zhong, Howard Feldman, Tristy Tara, Timo Grimmer, Carol Brayne, David Ash, Dennis Chan, Emma Barham, Guy Casy, John O’Brien, Judith Wilson, Lesley Pilgrim, Michele York, Nadja Smailagic, Natassia Brenman, Peter Geach, Philippa Farmer, Renata Schaeffer, Richard Milne, Sally Atkinson, Sarah Findon, Sarah Taylor, Shirlene Badger, Susan Black, Tazuko Edwards, Will Clark, Michele York, Zeynep Sahin, Babak Boroojerdi, Bernhard Greve, Daphne Derouane, David Marquet, Johannes Streffer, Katharina Angrosch, Mireille Delval, Peter Verplancke, Simon Dresse, Stefano Zanigni, Suzanne McCabe, Nick Fox, Pawel Markiewicz, Joel Kramer, Sabrina Erlhoff, Michael Schaffer, Adam Waldman, Alan Kennedy, Andy Kodiak, Angela Noble, Anna Anderson, Anna Borthwick, Anne Hall, Bill Bruce, Brian McTeir, Carol Di Perri, Cathie Sudlow, Chloe Kippen, Clare Dolan, Craig Ritchie, David Hill, David Matheson, Doug Young, Eirini Souri, Ellie Mcmaster, Emilie Delpon, Emma Law, Emma Munro, Fiona Campbell, Fiona Edler, Fiona Scott, Graciela Muniz Terrera, Hannah Jobse, Hazel Milligan, Hinesh Topiwala, Howard Marriage, Jean Manson, Jen Middleton, Joanna Swiderska, Joanna Wardlaw, Joanne MacConnell, Judi Syson, Julie Edwards, Kate Forsyth, Kathryn Carruthers, Katia Hervy, Katie Wells, Kristy Draper, Laura Doull, Laura Petrie, Lesley Niezynski, Lindsay Hampton, Loukia Koutsoventi, Lucy Stirland, Marise Bucukoglu, Molly Boughey, Natalie Jenkins, Neil Duncan, Neil Fullerton, Neil Mitchell, Ray French, Rebecca Flory, Ruaridh Buchan, Rustam Al Shahi-Salman, Samuel Danso, Sarah Gregory, Sarah Sparks, Stina Saunders, Susan Shepherd, Tamlyn Watermeyer, Tom MacGillivray, Tom Russ, Tyler Saunders, Val Renton, Vikki Leslie, Alexander Dzerzga, Britta Doelle, Christel Gatzke, Clause Escher, Denise Schneider, Lena Sannemann, Manuela Thelen, Petra Schreiner-Kaub, Simone Stockter, Susan Thielking, Theresa Mueller, Frank Jessen, Andrea Toja, Elena Chipi, Lucia Farotti, Lucilla Parnetti, Owen Lancaster, Anthony J. Brookes, Brian Berry, Colin Veal, Dhiwagaran Thangavelu, Jon Sole, Marie Adams, Monika Maini, Nickie Ketcher, Nicola A. Ketcher, Vagelis Ladas, Alexandra Agogue, Ana Campillo, Andreas Schmalz, Bianca Auschra, Blanche Pirotte, Christine Trombert, Daria Genaro, Elisa Canzoneri, Emiliano Albanese, Estefania Vilarino, Giovanni Battista Frisoni, Jennyfer Sturzmann, Margherita Mauri, Marina Boccardi, Mathilde Boillat, Maura M.P. Parapini, Monica Almici, Paulina Andryszak, Sven Haller, Tatjana Stevanovic, Christopher Kipps, Susan Jackson, Charlotte Frick, Henrik Zetterberg, Kaj Blennow, Linnea Petersson, Maria Berglund, Silke Kern, Alexandra Radford, Annalena Venneri, Joanne Sidebottom, Patrick Easton, Rosie Clegg, Walter Kullur, Tom Hartley, Amy Chinner, Antoniya Markova, Ash Sexton, Caroline Jenkins, Clare Mackay, Corinne Prescott, David Ruvolo, Delia Gheorghe, Denis Murphy, Gem Pope, Gill Cane, Gill Wells, Ivan Koychev, Jasmine Blane, Jennifer Lawson, John Gallacher, Juliana Ballaminut, Justin Lowen, Karla Westphal, Katherine Shepherd, Kay McNamee, Leona Wolters, Michael Ben Yehuda, Natalie Morgan, Neil Buckholtz, Nyla Haque, Patricia Burton, Sarah Bauermeister, Sarah Hoosdally, Shona Forster, Tequila Osborne, Vanessa Raymont, Ann van den Eynde, Sebastiaan Engelborghs, Rik Vandenberghe, Carine Schildermans, Alle Meije Wink, André Van der Wal, Aniko Hazelebach, Anna Elisabeth Leeuwis, Casper De Boer, Fiona Heeman, Frederik Barkhof, Isadora Lopes-Alves, Jeroen van Leur, Lea ter Meulen, Lisa Vermunt, Luigi Lorenzini, Lyduine Eisa Collij, Mara ten Kate, Martijn van Houten, Menno Stellingwerff, Merel Hermans, Michel Telkamp, Niels Prins, Philip Scheltens, Pieter Jelle Visser, Silvia Ingala, Wendy van Veen, Michelle McDonald, Clare Taylor, Genevieve Morrison, Mara Golemme, Paresh Malhotra, Sithuya Mahalingam, Christine van Broechhoven, Johan Goeman, Sebastiaan Engelborghs, Wendy Wittebolle.

## References

[1] Jack CR, Holtzman DM. Biomarker modeling of Alzheimer’s disease. Neuron. 2013;80(6):1347–1358. 24360540 10.1016/j.neuron.2013.12.003 PMC3928967

[2] Alzheimer’s Association . 2024 Alzheimer’s disease facts and figures. Alzheimers Dement. 2024;20(5):3708–3821. 38689398 10.1002/alz.13809 PMC11095490

[3] Jack CR, Andrews JS, Beach TG, et al Revised criteria for diagnosis and staging of Alzheimer’s disease: Alzheimer’s Association Workgroup. Alzheimers Dement. 2024;20:5143–5169. 38934362 10.1002/alz.13859 PMC11350039

[4] Boerwinkle AH, Wisch JK, Chen CD, et al Temporal correlation of CSF and neuroimaging in the amyloid-tau-neurodegeneration model of Alzheimer disease. Neurology. 2021;97(1):e76–e87. 33931538 10.1212/WNL.0000000000012123 PMC8312859

[5] Palmqvist S, Mattsson N, Hansson O. Cerebrospinal fluid analysis detects cerebral amyloid-β accumulation earlier than positron emission tomography. Brain. 2016;139(4):1226–1236. 26936941 10.1093/brain/aww015 PMC4806222

[6] Palmqvist S, Zetterberg H, Mattsson N, et al Detailed comparison of amyloid PET and CSF biomarkers for identifying early Alzheimer disease. Neurology. 2015;85(14):1240–1249. 26354982 10.1212/WNL.0000000000001991 PMC4607601

[7] Jack CR, Lowe VJ, Senjem ML, et al 11C PiB and structural MRI provide complementary information in imaging of Alzheimer’s disease and amnestic mild cognitive impairment. Brain. 2008;131(3):665–680. 18263627 10.1093/brain/awm336 PMC2730157

[8] Chételat G, Villemagne VL, Bourgeat P, et al Relationship between atrophy and β-amyloid deposition in Alzheimer disease. Ann Neurol. 2010;67(3):317–324. 20373343 10.1002/ana.21955

[9] La Joie R, Perrotin A, Barré L, et al Region-specific hierarchy between atrophy, hypometabolism, and β-amyloid (Aβ) load in Alzheimer’s disease dementia. J Neurosci. 2012;32(46):16265–16273. 23152610 10.1523/JNEUROSCI.2170-12.2012 PMC6794030

[10] Braak H, Braak E. Neuropathological staging of Alzheimer-related changes. Acta Neuropathol. 1991;82(4):239–259. 1759558 10.1007/BF00308809

[11] Johnson KA, Fox NC, Sperling RA, Klunk WE. Brain imaging in Alzheimer disease. Cold Spring Harb Perspect Med. 2012;2(4):a006213. 22474610 10.1101/cshperspect.a006213 PMC3312396

[12] Tissot CL, Benedet A, Therriault J, et al Plasma pTau181 predicts cortical brain atrophy in aging and Alzheimer’s disease. Alzheimers Res Ther. 2021;13(1):69. 33781319 10.1186/s13195-021-00802-x PMC8008680

[13] Ikonomovic MD, Klunk WE, Abrahamson EE, et al Post-mortem correlates of in vivo PiB-PET amyloid imaging in a typical case of Alzheimer’s disease. Brain. 2008;131(Pt 6):1630–1645. 18339640 10.1093/brain/awn016 PMC2408940

[14] Mattsson N, Insel PS, Donohue M, et al Independent information from cerebrospinal fluid amyloid-β and florbetapir imaging in Alzheimer’s disease. Brain. 2015;138(Pt 3):772–783. 25541191 10.1093/brain/awu367 PMC4339769

[15] Reimand J, De Wilde A, Teunissen CE, et al PET and CSF amyloid-β status are differently predicted by patient features: Information from discordant cases. Alzheimers Res Ther. 2019;11(1):100. 31810489 10.1186/s13195-019-0561-5 PMC6898919

[16] de Souza LC, Chupin M, Lamari F, et al CSF tau markers are correlated with hippocampal volume in Alzheimer’s disease. Neurobiol Aging. 2012;33(7):1253–1257. 21489655 10.1016/j.neurobiolaging.2011.02.022

[17] Apostolova LG, Hwang KS, Andrawis JP, et al 3D PIB and CSF biomarker associations with hippocampal atrophy in ADNI subjects. Neurobiol Aging. 2010;31(8):1284–1303. 20538372 10.1016/j.neurobiolaging.2010.05.003 PMC3051831

[18] Fjell AM, Walhovd KB, Amlien I, et al Morphometric changes in the episodic memory network and tau pathologic features correlate with memory performance in patients with mild cognitive impairment. AJNR Am J Neuroradiol. 2008;29(6):1183–1189. 18544670 10.3174/ajnr.A1059 PMC8118812

[19] Fagan AM, Head D, Shah AR, et al Decreased CSF Aβ42 correlates with brain atrophy in cognitively normal elderly. Ann Neurol. 2009;65(2):176–183. 19260027 10.1002/ana.21559 PMC2763631

[20] Ott BR, Cohen RA, Gongvatana A, et al Brain ventricular volume and cerebrospinal fluid biomarkers of Alzheimer’s disease. J Alzheimers Dis. 2010;20(2):647–657. 20182051 10.3233/JAD-2010-1406 PMC3078034

[21] Chou Y-Y, Leporé N, Avedissian C, et al Mapping correlations between ventricular expansion and CSF amyloid and tau biomarkers in 240 subjects with Alzheimer’s disease, mild cognitive impairment and elderly controls. NeuroImage. 2009;46(2):394–410. 19236926 10.1016/j.neuroimage.2009.02.015 PMC2696357

[22] Lidén S, Farahmand D, Laurell K. Ventricular volume in relation to lumbar CSF levels of amyloid-β 1–42, tau and phosphorylated tau in iNPH, is there a dilution effect? Fluids Barriers CNS. 2022;19(1):59. 35843939 10.1186/s12987-022-00353-9 PMC9288679

[23] Llibre-Guerra JJ, Li Y, Schindler SE, et al Association of longitudinal changes in cerebrospinal fluid total tau and phosphorylated tau 181 and brain atrophy with disease progression in patients with Alzheimer disease. JAMA Netw Open. 2019;2(12):e1917126. 31825500 10.1001/jamanetworkopen.2019.17126 PMC6991202

[24] Seidu NM, Kern S, Sacuiu S, et al Association of CSF biomarkers with MRI brain changes in Alzheimer’s disease. Alzheimers Dement. 2024;16(1):e12556. 10.1002/dad2.12556 PMC10884990 38406609

[25] Ingala S, De Boer C, Masselink LA, et al Application of the ATN classification scheme in a population without dementia: Findings from the EPAD cohort. Alzheimers Dement. 2021;17(7):1189–1204. 33811742 10.1002/alz.12292 PMC8359976

[26] Lorenzini L, Ingala S, Wottschel V, et al Gray matter network properties show distinct associations with CSF p-tau 181 levels and amyloid status in individuals without dementia. Aging Brain. 2022;2:100054. 36908898 10.1016/j.nbas.2022.100054 PMC9997148

[27] Koran MEI, Wagener M, Hohman TJ. Sex differences in the association between AD biomarkers and cognitive decline. Brain Imaging Behav. 2017;11(1):205–213. 26843008 10.1007/s11682-016-9523-8 PMC4972701

[28] Beekly DL, Ramos EM, Lee WW, et al The National Alzheimer’s Coordinating Center (NACC) database: The uniform data set. Alzheimer Dis Assoc Disord. 2007;21(3):249–258. 17804958 10.1097/WAD.0b013e318142774e

[29] Petersen RC, Aisen PS, Beckett LA, et al Alzheimer’s Disease Neuroimaging Initiative (ADNI) clinical characterization. Neurology. 2010;74(3):201–209. 20042704 10.1212/WNL.0b013e3181cb3e25 PMC2809036

[30] Lorenzini L, Ingala S, Wink AM, et al The Open-Access European Prevention of Alzheimer’s Dementia (EPAD) MRI dataset and processing workflow. Neuroimage Clin. 2022;35:103106. 35839659 10.1016/j.nicl.2022.103106 PMC9421463

[31] Billot B, Magdamo C, Cheng Y, Arnold SE, Das S, Iglesias JE. Robust machine learning segmentation for large-scale analysis of heterogeneous clinical brain MRI datasets. Proc Natl Acad Sci U S A. 2023;120(9):e2216399120. 36802420 10.1073/pnas.2216399120 PMC9992854

[32] Fong TG, Vasunilashorn SM, Gou Y, et al Association of CSF Alzheimer’s disease biomarkers with postoperative delirium in older adults. Alzheimers Dement. 2021;7(1):e12125. 10.1002/trc2.12125 PMC7968120 33748398

[33] Trombetta BA, Wu C-Y, Kuo E, et al Cerebrospinal fluid biomarker profiling of diverse pathophysiological domains in Alzheimer’s disease. Alzheimers Dement. 2024;10(1):e12440. 10.1002/trc2.12440 PMC10865489 38356471

[34] Viechtbauer W . Conducting meta-analyses in R with the metafor package. J Stat Softw. 2010;36(3):1–48.

[35] Kong RQ, Spreng RN, Xue A, et al A network correspondence toolbox for quantitative evaluation of novel neuroimaging results. Nat Commun. 2025;16(1):2930. 40133295 10.1038/s41467-025-58176-9 PMC11937327

[36] Andrews-Hanna JR . The brain’s default network and its adaptive role in internal mentation. Neuroscientist. 2012;18(3):251–270. 21677128 10.1177/1073858411403316 PMC3553600

[37] Moreau N, Rauzy S, Viallet F, Champagne-Lavau M. Theory of mind in Alzheimer disease: Evidence of authentic impairment during social interaction. Neuropsychology. 2016;30(3):312–321. 26146852 10.1037/neu0000220

[38] Tosun D, Schuff N, Truran-Sacrey D, et al Relations between brain tissue loss, CSF biomarkers, and the ApoE genetic profile: A longitudinal MRI study. Neurobiol Aging. 2010;31(8):1340–1354. 20570401 10.1016/j.neurobiolaging.2010.04.030 PMC2902689

[39] Wang L, Benzinger TL, Hassenstab J, et al Spatially distinct atrophy is linked to β-amyloid and tau in preclinical Alzheimer disease. Neurology. 2015;84(12):1254–1260. 25716355 10.1212/WNL.0000000000001401 PMC4366088

[40] Seidu NM, Kern S, Sacuiu S, et al Associations between cerebrospinal fluid biomarkers and brain regions of relevance for very early Alzheimer’s disease processes. Alzheimers Dement. 2023;19(S2):1–2.

[41] Palmqvist S, Schöll M, Strandberg O, et al Earliest accumulation of β-amyloid occurs within the default-mode network and concurrently affects brain connectivity. Nat Commun. 2017;8(1):1214. 29089479 10.1038/s41467-017-01150-x PMC5663717

[42] Grimmer T, Faust M, Auer F, et al White matter hyperintensities predict amyloid increase in Alzheimer’s disease. Neurobiol Aging. 2012;33(12):2766–2773. 22410648 10.1016/j.neurobiolaging.2012.01.016

[43] Al-Ani L, Tao A, Dyke J, Chiang G, Ishii M. Amygdala atrophy as an early manifestation of Alzheimer’s disease (S39.005). Neurology. 2023;100(Suppl 2):1800.

[44] Nosheny RL, Insel PS, Mattsson N, et al Associations among amyloid status, age, and longitudinal regional brain atrophy in cognitively unimpaired older adults. Neurobiol Aging. 2019;82:110–119. 31437719 10.1016/j.neurobiolaging.2019.07.005 PMC7198229

[45] Kerbler GM, Fripp J, Rowe CC, et al Basal forebrain atrophy correlates with amyloid β burden in Alzheimer’s disease. Neuroimage Clin. 2015;7:105–113. 25610772 10.1016/j.nicl.2014.11.015 PMC4299972

[46] Forno G, Saranathan M, Contador J, et al Thalamic nuclei changes in early and late onset Alzheimer’s disease. Curr Res Neurobiol. 2023;4:100084. 37397807 10.1016/j.crneur.2023.100084 PMC10313877

[47] Cole G, Neal JW, Singhrao SK, Jasani B, Newman GR. The distribution of amyloid plaques in the cerebellum and brain stem in Down’s syndrome and Alzheimer’s disease: A light microscopical analysis. Acta Neuropathol. 1993;85(5):542–552. 8493862 10.1007/BF00230495

[48] Larner AJ . The cerebellum in Alzheimer’s disease. Dement Geriatr Cogn Disord. 1997;8(4):203–209. 9213064 10.1159/000106632

[49] Brilliant M, Elble RJ, Ghobrial M, Struble RG. Distribution of amyloid in the brainstem of patients with Alzheimer disease. Neurosci Lett. 1992;148(1–2):23–26. 1300498 10.1016/0304-3940(92)90795-9

[50] Cheng Y, Ho E, Weintraub S, et al Predicting brain amyloid status using the National Institute of Health Toolbox (NIHTB) for assessment of neurological and behavioral function. J Prev Alzheimers Dis. 2024;11:943–957. 39044505 10.14283/jpad.2024.77 PMC11269772

[51] Schäfer A, Chaggar P, Thompson TB, Goriely A, Kuhl E. Predicting brain atrophy from tau pathology: A summary of clinical findings and their translation into personalized models. Brain Multiphys. 2021;2:100039.

[52] Sluimer JD, Bouwman FH, Vrenken H, et al Whole-brain atrophy rate and CSF biomarker levels in MCI and AD: A longitudinal study. Neurobiol Aging. 2010;31(5):758–764. 18692273 10.1016/j.neurobiolaging.2008.06.016

[53] He Y, Wang L, Zang Y, et al Regional coherence changes in the early stages of Alzheimer’s disease: A combined structural and resting-state functional MRI study. NeuroImage. 2007;35(2):488–500. 17254803 10.1016/j.neuroimage.2006.11.042

[54] De Marco M, Duzzi D, Meneghello F, Venneri A. Cognitive efficiency in Alzheimer’s disease is associated with increased occipital connectivity. J Alzheimers Dis. 2017;57(2):541–556. 28269781 10.3233/JAD-161164

[55] Yeo BT, Krienen FM, Sepulcre J, et al The organization of the human cerebral cortex estimated by intrinsic functional connectivity. J Neurophysiol. 2011;106(3):1125–1165. 21653723 10.1152/jn.00338.2011 PMC3174820

[56] Kong R, Yang Q, Gordon E, et al Individual-specific areal-level parcellations improve functional connectivity prediction of behavior. Cereb Cortex. 2021;31(10):4477–4500. 33942058 10.1093/cercor/bhab101 PMC8757323

[57] Lindbergh CA, Casaletto KB, Staffaroni AM, et al Sex-related differences in the relationship between β-amyloid and cognitive trajectories in older adults. Neuropsychology. 2020;34(8):835–850. 33030915 10.1037/neu0000696 PMC7839841

[58] Buckley RF, Mormino EC, Amariglio RE, et al Sex, amyloid, and APOE ε4 and risk of cognitive decline in preclinical Alzheimer’s disease: Findings from three well-characterized cohorts. Alzheimers Dement. 2018;14(9):1193–1203. 29803541 10.1016/j.jalz.2018.04.010 PMC6131023

[59] Vila-Castelar C, Akinci M, Palpatzis E, et al Sex/gender effects of glial reactivity on preclinical Alzheimer’s disease pathology. Mol Psychiatry. 2025;30(4):1430–1439. 39384963 10.1038/s41380-024-02753-9 PMC11919761

[60] Resnick SM, Espeland MA, Jaramillo SA, et al Postmenopausal hormone therapy and regional brain volumes: The WHIMS-MRI study. Neurology. 2009;72(2):135–142. 19139364 10.1212/01.wnl.0000339037.76336.cf PMC2677493

