# Supplementary material for: Brain MRI signatures across sex and CSF Alzheimer’s disease biomarkers
Source: Brain Commun. 2025 May 30;7(3):fcaf210. doi: 10.1093/braincomms/fcaf210 (PMC12152538; doi:10.1093/braincomms/fcaf210)
Supplement: fcaf210_Supplementary_Data [file fcaf210_supplementary_data.docx]

# Supplementary Materials

## Supplementary Figures


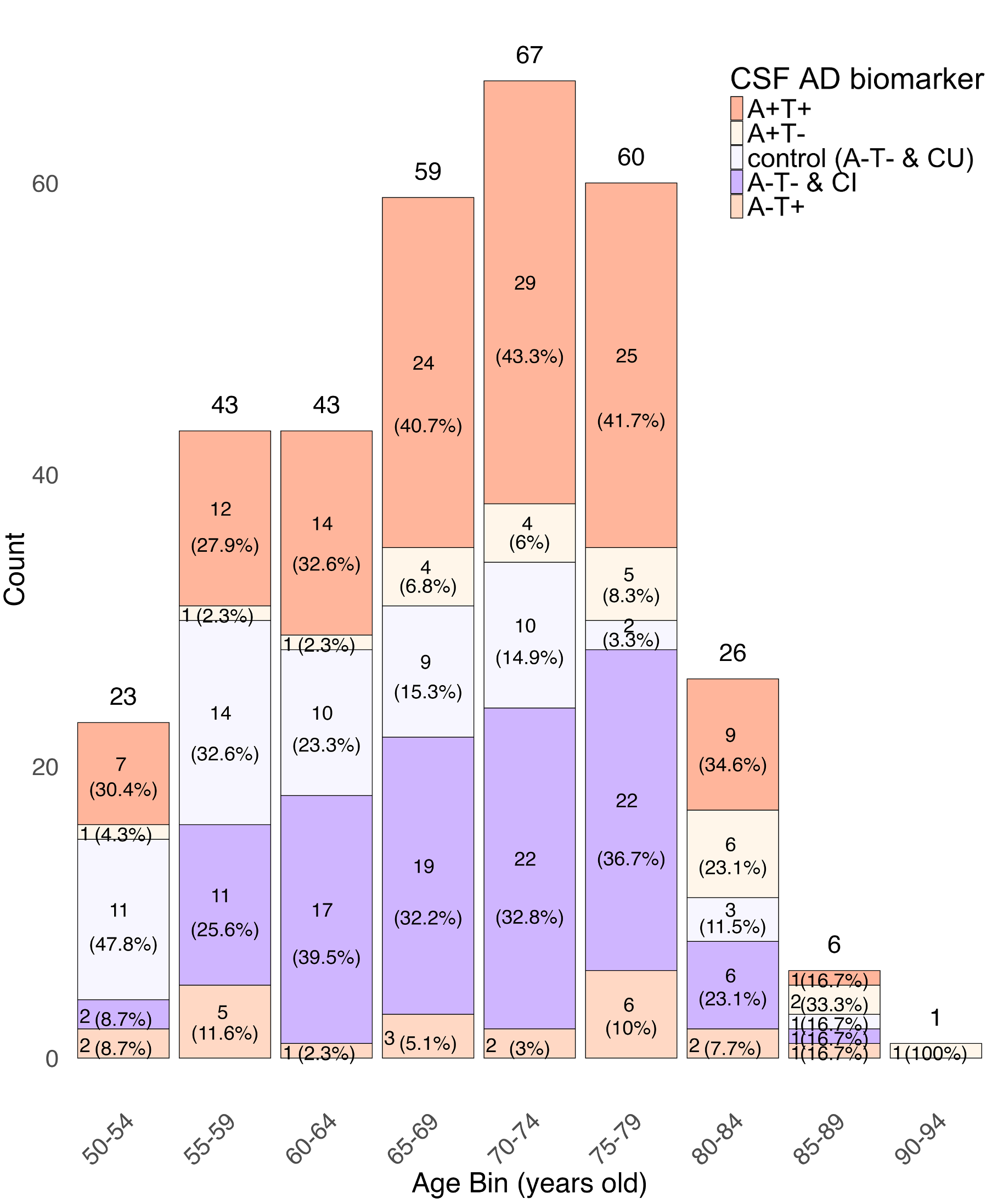


**Supplementary Figure 1. Stacked bar plots of patients’ age distribution (N=328), characterized by CSF Alzheimer’s disease biomarker groups.** Number on top of each bar represents the total number of patients of the corresponding age range. Numbers within each bar represents the number of patients with corresponding Alzheimer’s disease biomarker groups in the corresponding age range while percentage represents the percentage of participants in the CSF biomarker groups in the total participants of that age range. Amyloid positive tau negative (A+T-) was defined by *Aß42/40 ratio* (*ABR*) < 0.082 and *p-Tau* 181 ≤ 41.8 pg/mL. Amyloid negative tau positive (A-T+) was defined by *ABR* ≥ 0.082 and *p-Tau 181* ≤ 41.8 pg/mL. Amyloid positive tau positive (A+T+) was defined by *ABR* < 0.082 and *p-Tau 181* > 41.8 pg/mL. The control group (amyloid negative tau negative, A-T-) was defined by *ABR* ≥ 0.082, *p-Tau 181* ≤ 41.8 pg/mL, and cognitively unimpaired (CU). CI: cognitively impaired.

**
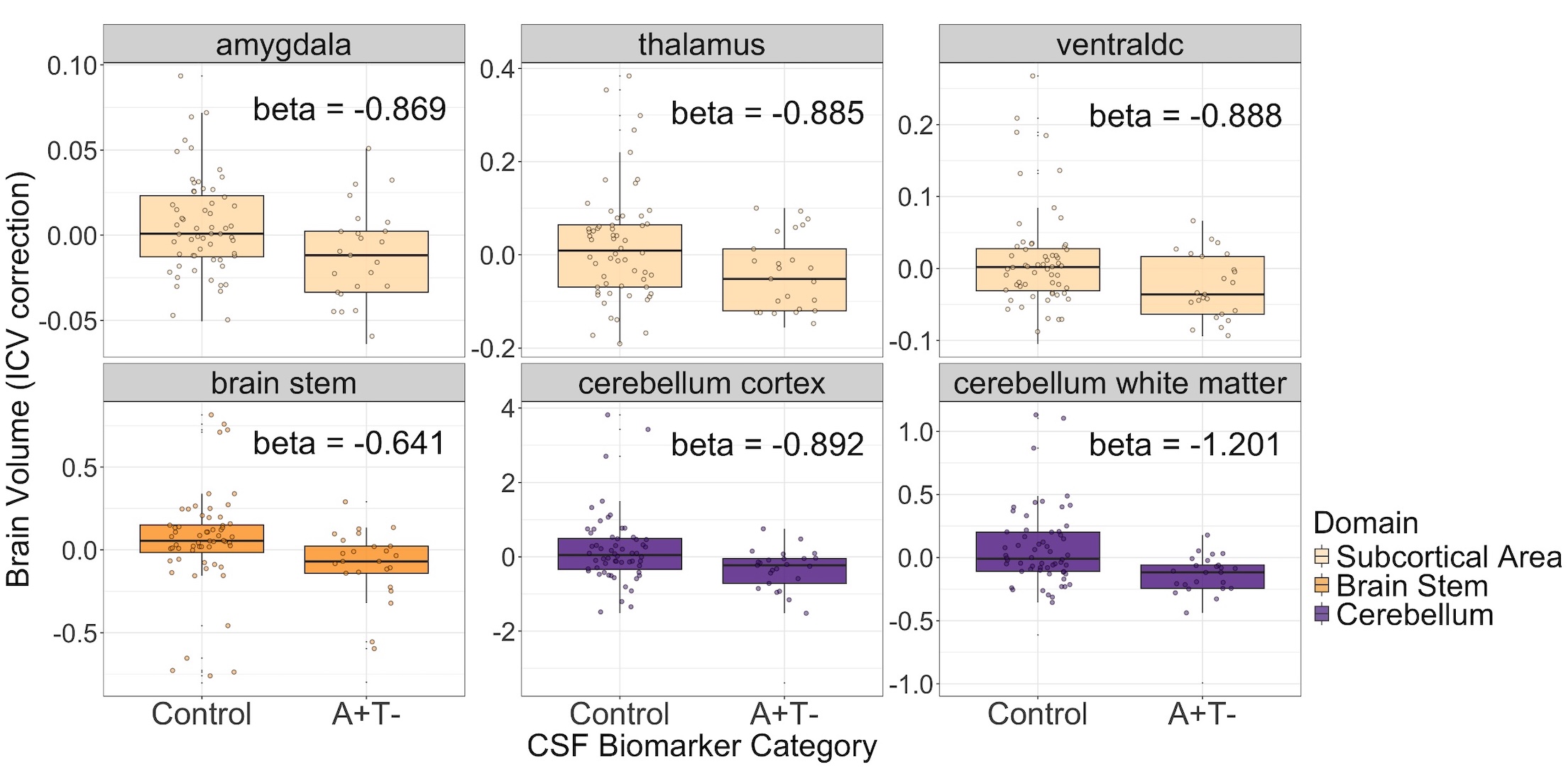
**

**Supplementary Figure 2. Box plots showing brain volumes associated with amyloid.** Features that were significantly associated with amyloid status (brain volumes were adjusted for age and sex) in logistic regression tests for patients equal or above 50 years old. Beta values were log odds. Each individual data point represents ICV-adjusted brain volume of one participant. Sample sizes: A+T- (N = 25), Control (N = 100). A+T-: amyloid positive tau negative. ICV: intracranial volume.

**
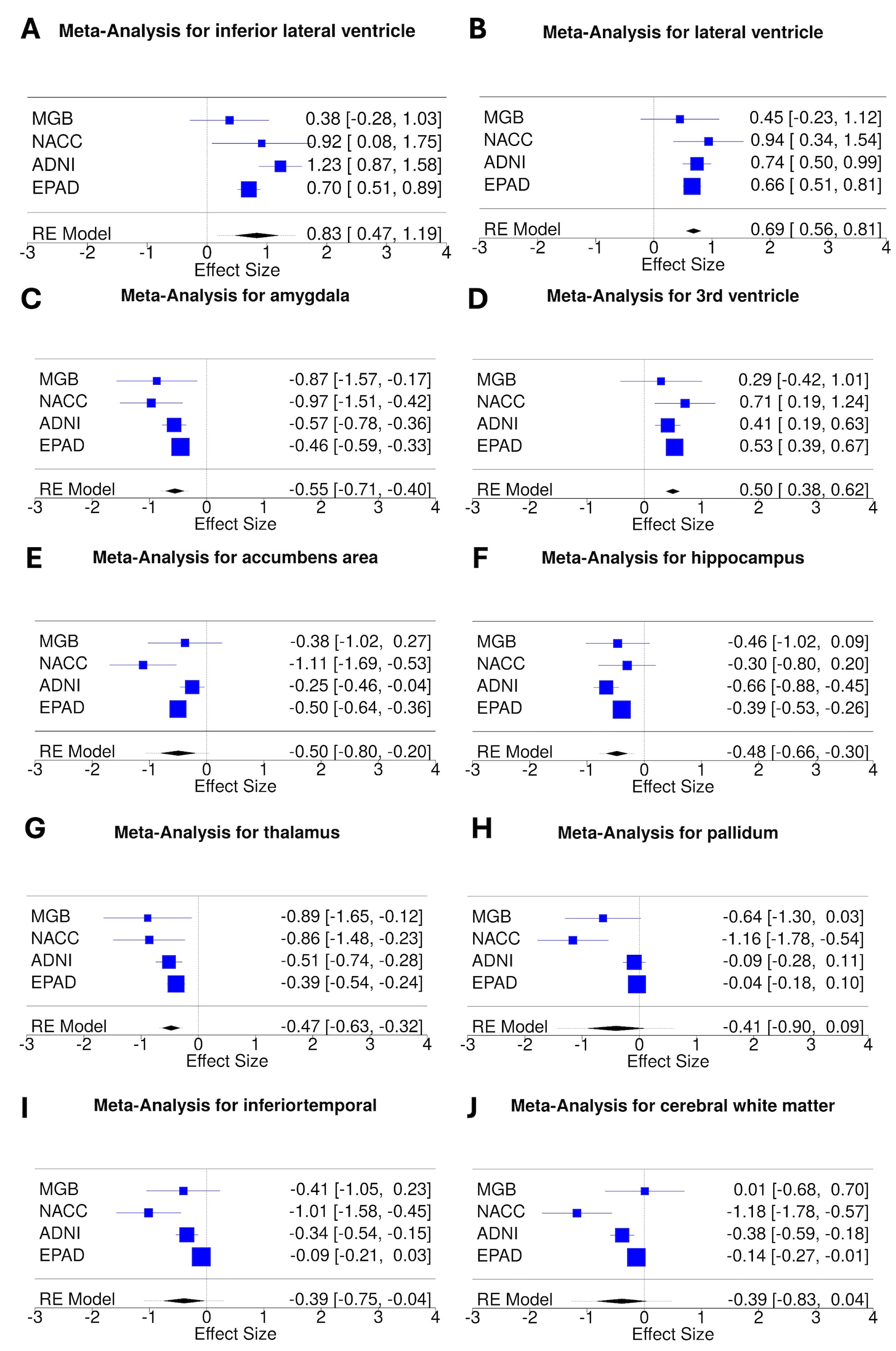
**

**Supplementary Figure 3.** Meta-analysis of brain volumes significantly associated with amyloid status. Forest plots of top 10 brain areas with largest effect sizes from the random-effects model with REML estimation. Each box represents the effect size from a logistic regression analysis in an individual dataset, where the center marks the mean, the box size reflects the sample size, and the horizontal lines indicate the confidence interval. The bottom row in each subplot shows the overall meta-analytic effect size. Sample sizes: MGB (total: 125; A+T−: 25, Control: 100), NACC (total: 47; A+T−: 39, Control: 8), ADNI (total: 575; A+T−: 280, Control: 295), EPAD (total: 615; A+T−: 432, Control: 183). RE model: Random-Effects model. REML: Restricted Maximum Likelihood. A+T-: amyloid positive, tau negative. MGB: Mass General Brigham. NACC: National Alzheimer’s Coordinating Center. ADNI: Alzheimer’s Disease Neuroimaging Initiative. EPAD: European Prevention of Alzheimer’s Dementia).

**
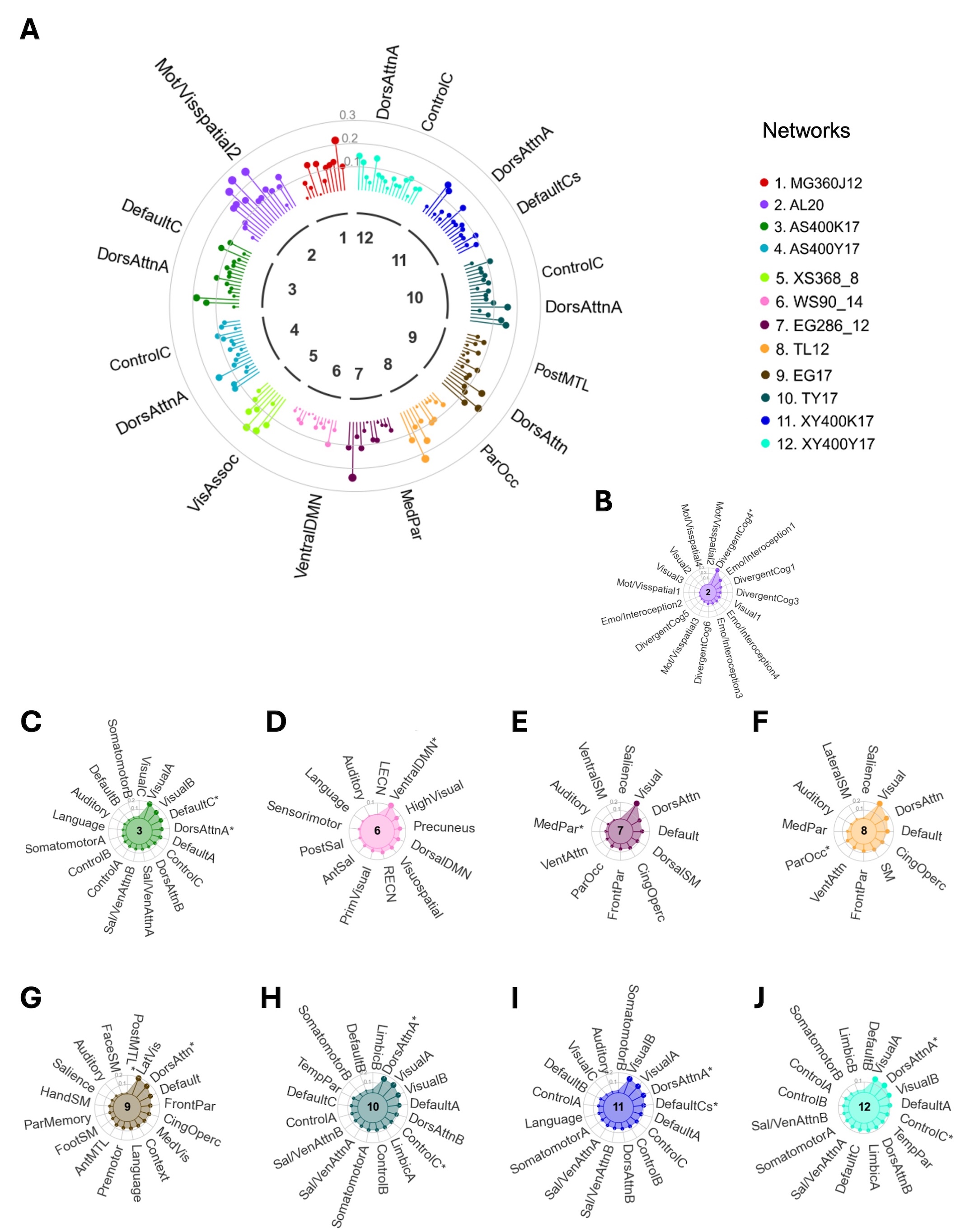
**

**Supplementary Figure 4. Functional networks significantly overlap with amyloid-associated brain volumes.** This includes a circular bar chart at the top demonstrating 11 atlases identified significant overlap brain regions (12 brain regions as highlighted in Table 2, excluding ventricles) significantly negatively associated with amyloid in at least one function network (*ps* < 0.05) using spin tests and the radar plots of the results from each network (except the network already displayed in the main figure). The brain parcellation atlases: MG360J12 – Glasser et al. (2016) 360-ROI atlas combined with Ji et al. (2019) 12 Cole–Anticevic networks; AL20 – Laird et al. (2011) 20-node ICA maps; AS400K17 and AS400Y17 – Schaefer et al. (2018) 400-ROI atlas using network labels from Kong et al. (2021) and Yeo et al. (2011), respectively; XS368_8 – Shen et al. (2013) 368-ROI parcellation with 8-network grouping; WS90_14 – Shirer et al. (2012) 90-ROI, 14-network atlas; EG286_12 – Gordon et al. (2016) 286-ROI atlas with 12 networks; TL12 – Laumann et al. (2015) 12-network parcellation based on Power et al. (2011); EG17 – Gordon et al. (2017) 17-network atlas; TY17 – Yeo et al. (2011) 17-network atlas; XY400K17 and XY400Y17 – Yan et al. (2023) 400-ROI atlas with network definitions from Kong et al. (2021) and Yeo et al. (2011), respectively. Network abbreviations include: DefaultA/B/C – Default Mode Network A/B/C; ControlA/B/C – Frontoparietal Control Network A/B/C; VisualA/B/2 – Visual Network A/B/2; SalVenAttnA/B – Salience/Ventral Attention Network A/B; DorsAttnA/B – Dorsal Attention Network A/B; SomatomotorA/B – Somatomotor Network A/B; LimbicA/B – Limbic Network A/B; MedPar – Medial Parietal Network; ParOcc – Parietal-Occipital Network; TempPar – Temporoparietal Network; VentralDMN – Ventral Default Mode Network; VisAssoc – Visual Association Network; PostMTL – Posterior Medial Temporal Lobe Network; FrontPar – Frontal-Parietal Network; Language – Language Network; Auditory – Auditory Network; CingOperc – Cingulo-Opercular Network; Context – Contextual Association Network; ParMemory – Parietal Memory Network; OrbitAffective – Orbitofrontal/Affective Network; LatVis – Lateral Visual Network; MedVis – Medial Visual Network; HighVisual – High-Level Visual Network; Sensorimotor – Sensorimotor Network; LECN/RECN – Left/Right Executive Control Networks; Primary – Primary Visual Network; DivergentCog1–3 – Divergent Cognitive Subnetworks; Emot/Interoception1–2 – Emotional/Interoceptive Subnetworks. Asterisks (*) indicate networks with significant overlap. The *p value* was based on the spin test permutations of the Dice coefficients.

**
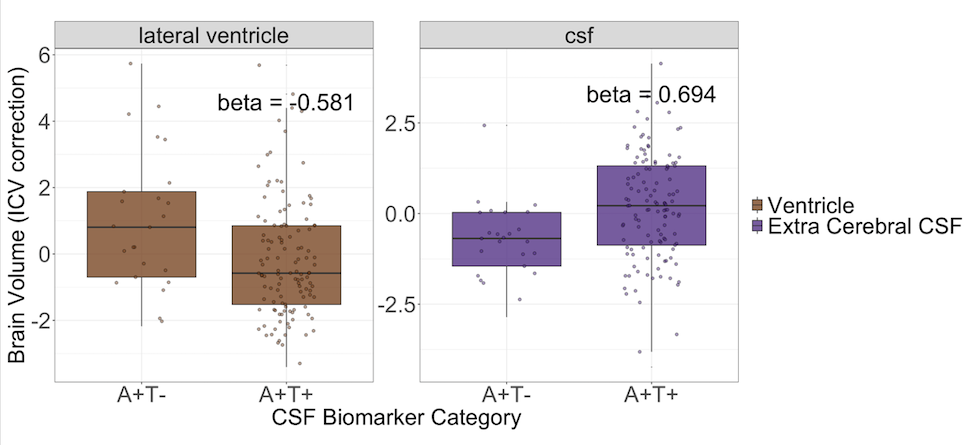
**

**Supplementary Figure 5. Box plots showing brain volumes associated with tau in the presence of amyloid.** Features that were significantly associated with tau status in the presence of amyloid (brain volumes were adjusted for age and sex) in logistic regression tests for patients equal or above 50 years old. Beta values were log odds. Each individual data point represents ICV-adjusted brain volume of one participant. Sample sizes: A+T- (N = 25), A+T+ (N = 121). A+T-: amyloid positive tau negative. A+T+: amyloid positive tau positive. ICV: intracranial volume.

**
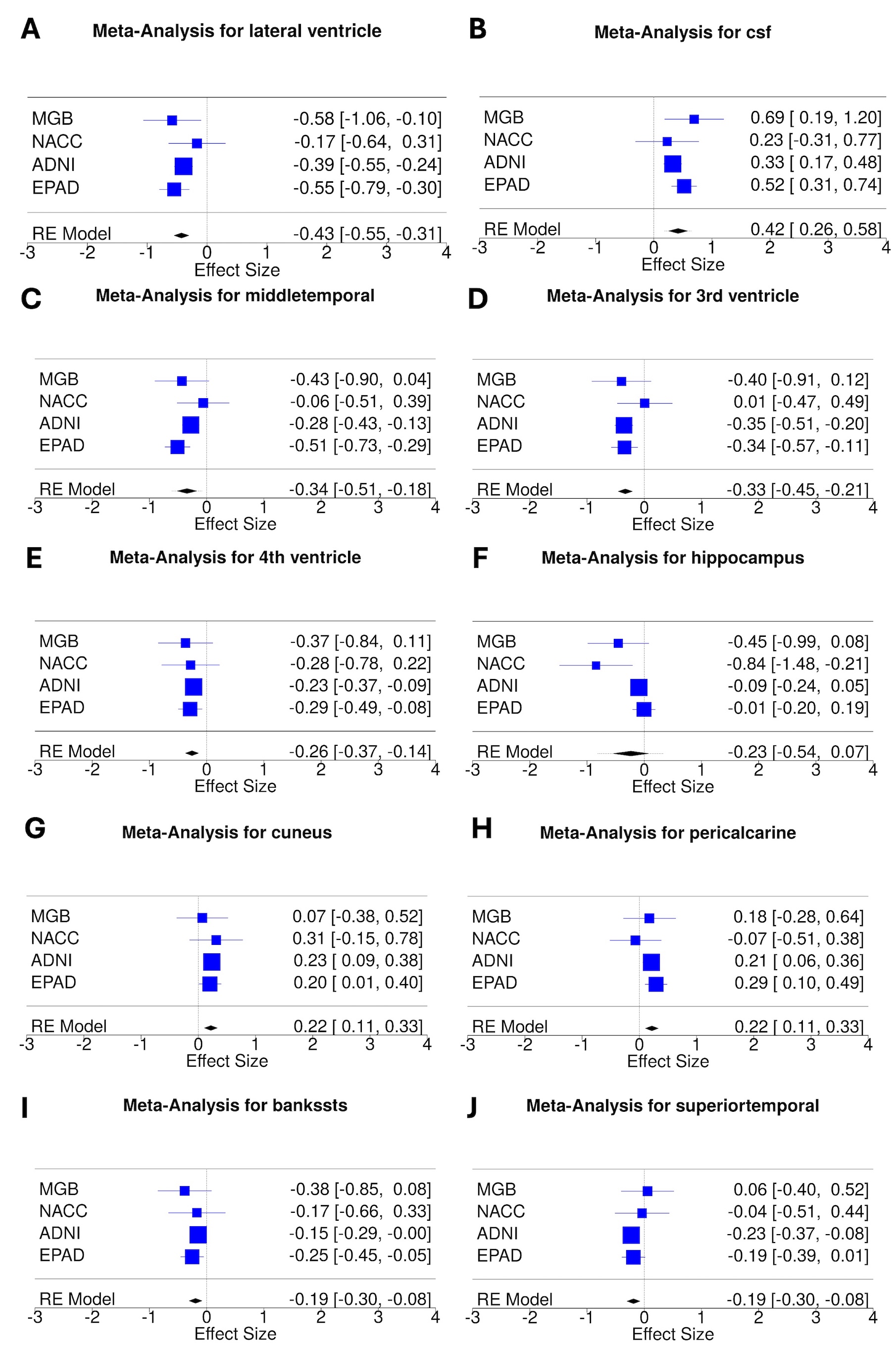
**

**Supplementary Figure 6.** Forest plot of 10 brain areas where the brain volumes were significant associated with tau status in the presence of amyloid and had the largest effect sizes from the random-effects model with REML estimation. Each box represents the effect size from a logistic regression analysis in an individual dataset, where the center marks the mean, the box size reflects the sample size, and the horizontal lines indicate the confidence interval. The bottom row in each subplot shows the overall meta-analytic effect size. Sample sizes: MGB (total: 146; A+T−: 25, A+T+: 121), NACC (total: 81; A+T−: 39, A+T+: 42), ADNI (total: 852; A+T−: 280, A+T+: 572), EPAD (total: 597; A+T−: 432, A+T+: 165). RE model: Random-Effects model. REML: Restricted Maximum Likelihood. A+T-: amyloid positive, tau negative. A+T+: amyloid positive, tau positive. MGB: Mass General Brigham. NACC: National Alzheimer’s Coordinating Center. ADNI: Alzheimer’s Disease Neuroimaging Initiative. EPAD: European Prevention of Alzheimer’s Dementia.

**
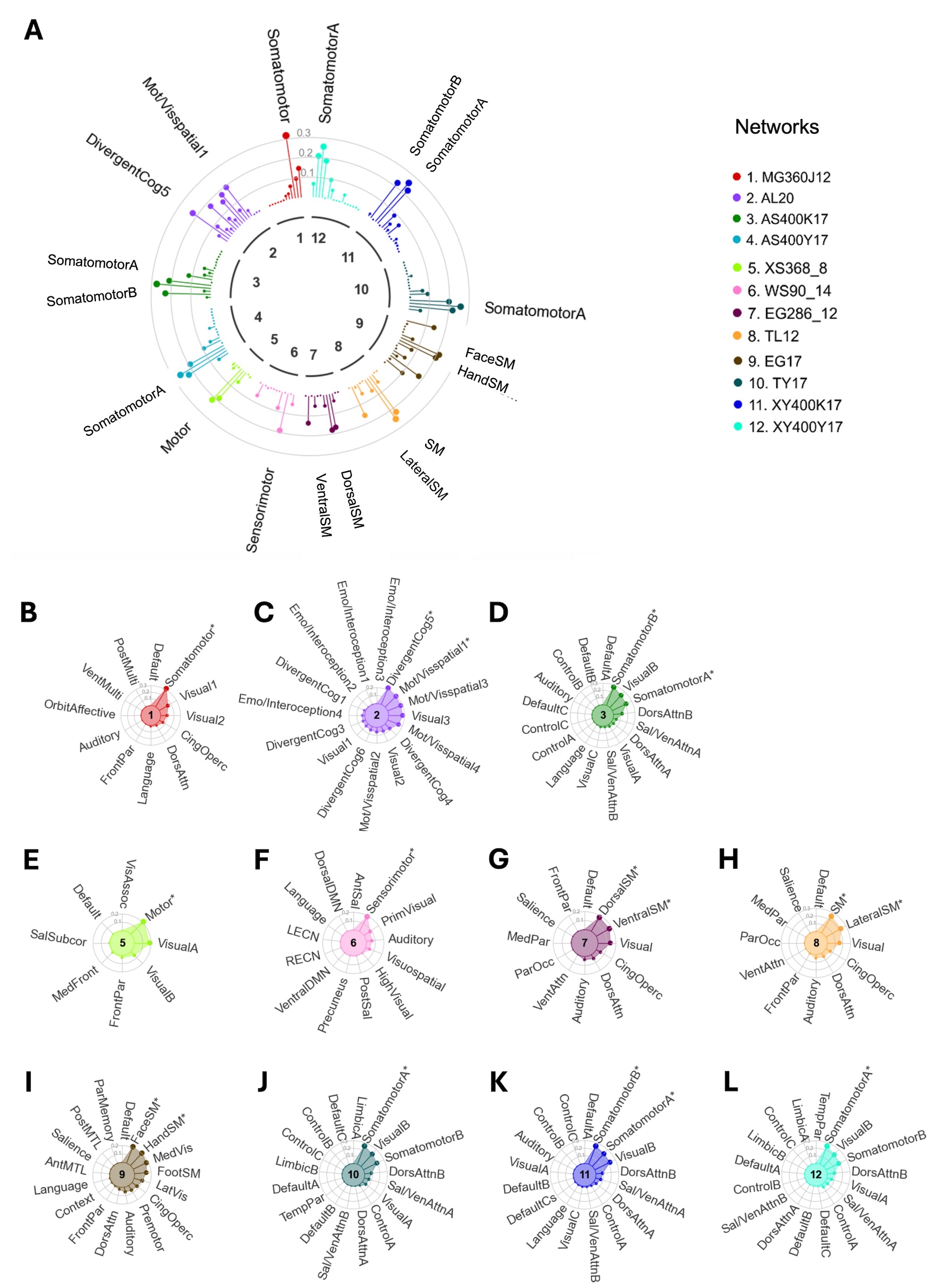
**

**Supplementary Figure 7. Functional networks show significant overlap with brain volumes positively associated with tau in the presence of amyloid.** At the top, a circular bar chart highlights that all 12 atlases identified significant overlap brain regions (5 brain regions as highlighted in Table 3, excluding csf) significantly positively associated with tau in the presence of amyloid (*ps* < 0.05) in at least one functional network using spin tests. Radar plots display results from each atlas with significant overlap, excluding the network already shown in the main figure. The brain parcellation atlases: MG360J12 – Glasser et al. (2016) 360-region atlas with Ji et al. (2019) 12-network Cole–Anticevic system; AL20 – Laird et al. (2011) 20-node ICA maps; AS400K17 and AS400Y17 – Schaefer et al. (2018) 400-ROI parcellations with Kong et al. (2021) and Yeo et al. (2011) 17-network assignments, respectively; XS368_8 – Shen et al. (2013) 368-region atlas grouped into 8 networks; WS90_14 – Shirer et al. (2012) 90-region atlas with 14 functional networks; EG286_12 – Gordon et al. (2016) 286-region atlas with 12-network organization; TL12 – Laumann et al. (2015) 12-network partition based on Power et al. (2011); EG17 – Gordon et al. (2017) 17-network atlas; TY17 – Yeo et al. (2011) 17-network atlas; XY400K17 and XY400Y17 – Yan et al. (2023) 400-ROI parcellations with Kong et al. (2021) and Yeo et al. (2011) 17-network definitions, respectively. Functional network abbreviations include: DefaultA/B/C – Default Mode Network A/B/C; ControlA/B/C – Frontoparietal Control Network A/B/C; VisualA/B/2/3 – Visual Networks; SalVenAttnA/B – Salience/Ventral Attention Network A/B; DorsAttnA/B – Dorsal Attention Network A/B; SomatomotorA/B – Somatomotor Network A/B; LimbicA/B – Limbic Network A/B; MedPar – Medial Parietal; ParOcc – Parietal-Occipital; TempPar – Temporoparietal; VentralDMN – Ventral Default Mode Network; VisAssoc – Visual Association; PostMTL – Posterior Medial Temporal Lobe; FrontPar – Frontal-Parietal; Language – Language Network; Auditory – Auditory Network; CingOperc – Cingulo-Opercular; Context – Contextual Association; ParMemory – Parietal Memory; OrbitAffective – Orbitofrontal/Affective; LatVis – Lateral Visual; MedVis – Medial Visual; HighVisual – High-Level Visual; Sensorimotor – Sensorimotor Network; LECN/RECN – Left/Right Executive Control Network; Primary – Primary Visual; SalSubcor – Salience/Subcortical; FootSM, HandSM, FaceSM – Somatomotor subregions; DivergentCog1–5 – Divergent Cognitive Subnetworks; Emot/Interoception1–4 – Emotional/Interoceptive Subnetworks; MoVisspatial1–4 – Motor/Visual-Spatial Subnetworks. Asterisks (*) indicate networks with significant spatial overlap. The *p value* was based on the spin test permutations of the Dice coefficients.


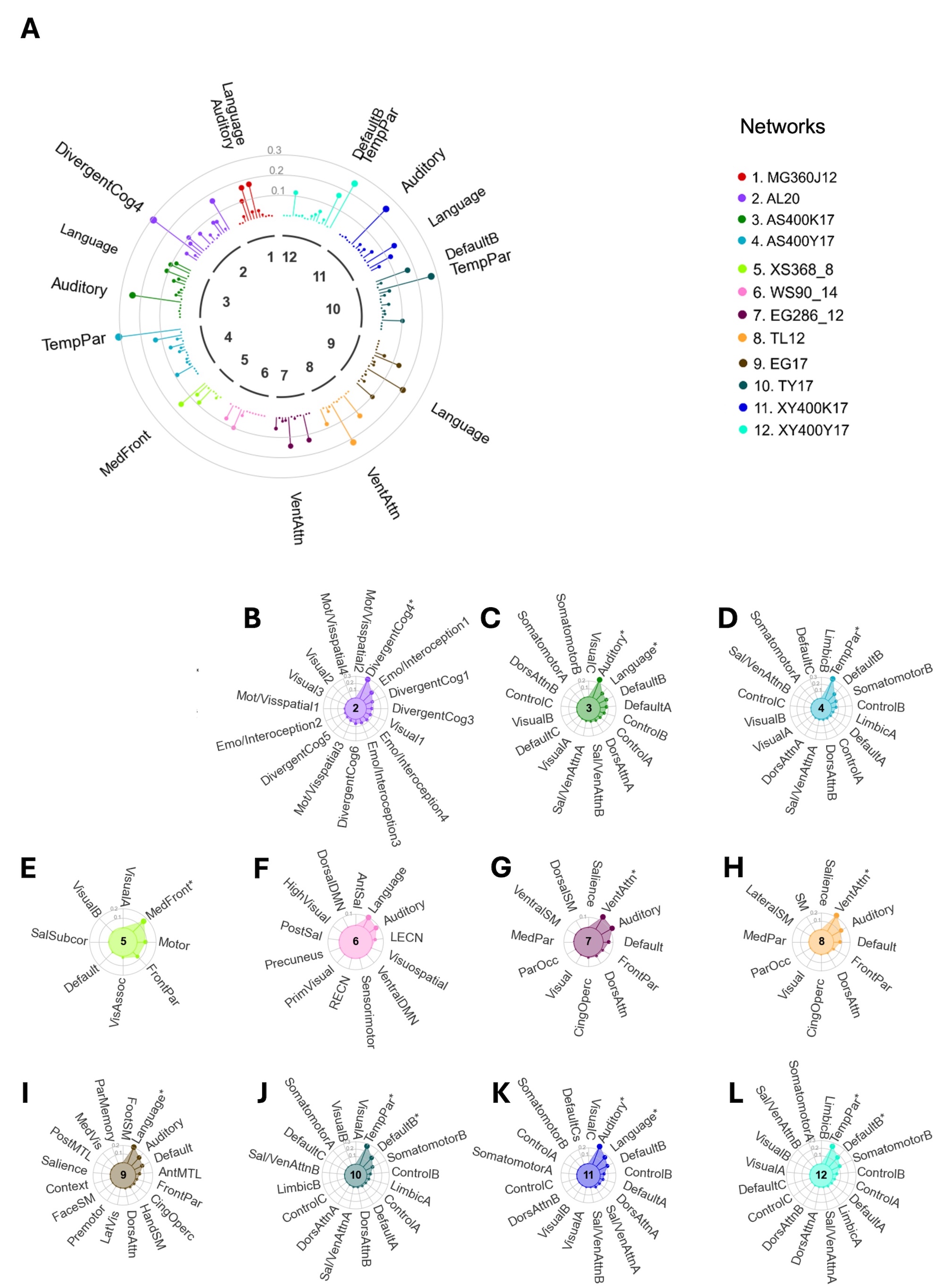


**Supplementary Figure 8. Functional networks show significant overlap with brain volumes negatively associated with tau in the presence of amyloid.** At the top, a circular bar chart highlights that all 12 atlases identified significant overlap brain regions (3 brain regions as highlighted in Table 3, excluding ventricles) significantly negatively associated with tau in the presence of amyloid (*ps* < 0.05) in at least one functional network using spin tests. Radar plots display results from each atlas with significant overlap, excluding the network already shown in the main figure. The brain parcellation atlases: MG360J12 – Glasser et al. (2016) 360-region parcellation with Ji et al. (2019) 12-network Cole–Anticevic mapping; AL20 – Laird et al. (2011) 20-node ICA parcellation; AS400K17 and AS400Y17 – Schaefer et al. (2018) 400-region parcellations with Kong et al. (2021) and Yeo et al. (2011) 17-network labels, respectively; XS368_8 – Shen et al. (2013) 368-region atlas grouped into 8 networks; WS90_14 – Shirer et al. (2012) 90-region, 14-network atlas; EG286_12 – Gordon et al. (2016) 286-region parcellation with 12-network structure; TL12 – Laumann et al. (2015) 12-network definition based on Power et al. (2011); EG17 – Gordon et al. (2017) 17-network atlas; TY17 – Yeo et al. (2011) 17-network atlas; XY400K17 and XY400Y17 – Yan et al. (2023) 400-region parcellations labeled using Kong et al. (2021) and Yeo et al. (2011) network schemes, respectively. Functional network abbreviations include: DefaultA/B/C – Default Mode Network A/B/C; ControlA/B/C – Frontoparietal Control Network A/B/C; VisualA/B/2/3 – Visual Networks; SalVenAttnA/B – Salience/Ventral Attention Networks A/B; DorsAttnA/B – Dorsal Attention Network A/B; SomatomotorA/B – Somatomotor Network A/B; LimbicA/B – Limbic Network A/B; Language – Language Network; Auditory – Auditory Network; MedFront – Medial Frontal Network; TempPar – Temporoparietal Network; ParOcc – Parietal-Occipital Network; MedPar – Medial Parietal Network; PostMTL – Posterior Medial Temporal Lobe; VentAttn – Ventral Attention; SalSubcor – Salience/Subcortical; OrbitAffective – Orbitofrontal/Affective; CingOperc – Cingulo-Opercular; LECN/RECN – Left/Right Executive Control Networks; Context – Contextual Association; ParMemory – Parietal Memory; HighVisual – High-Level Visual Network; Primary – Primary Visual; AntMTL – Anterior Medial Temporal Lobe; FootSM, HandSM, FaceSM – Somatomotor subregions (Foot, Hand, Face); Emot/Interoception1–4 – Emotional/Interoceptive Networks; DivergentCog1–5 – Divergent Cognitive Networks; Mot/Visospatial1–4 – Motor/Visual-Spatial Networks. Asterisks (*) indicate networks with significant spatial overlap. The *p value* was based on the spin test permutations of the Dice coefficients.

**
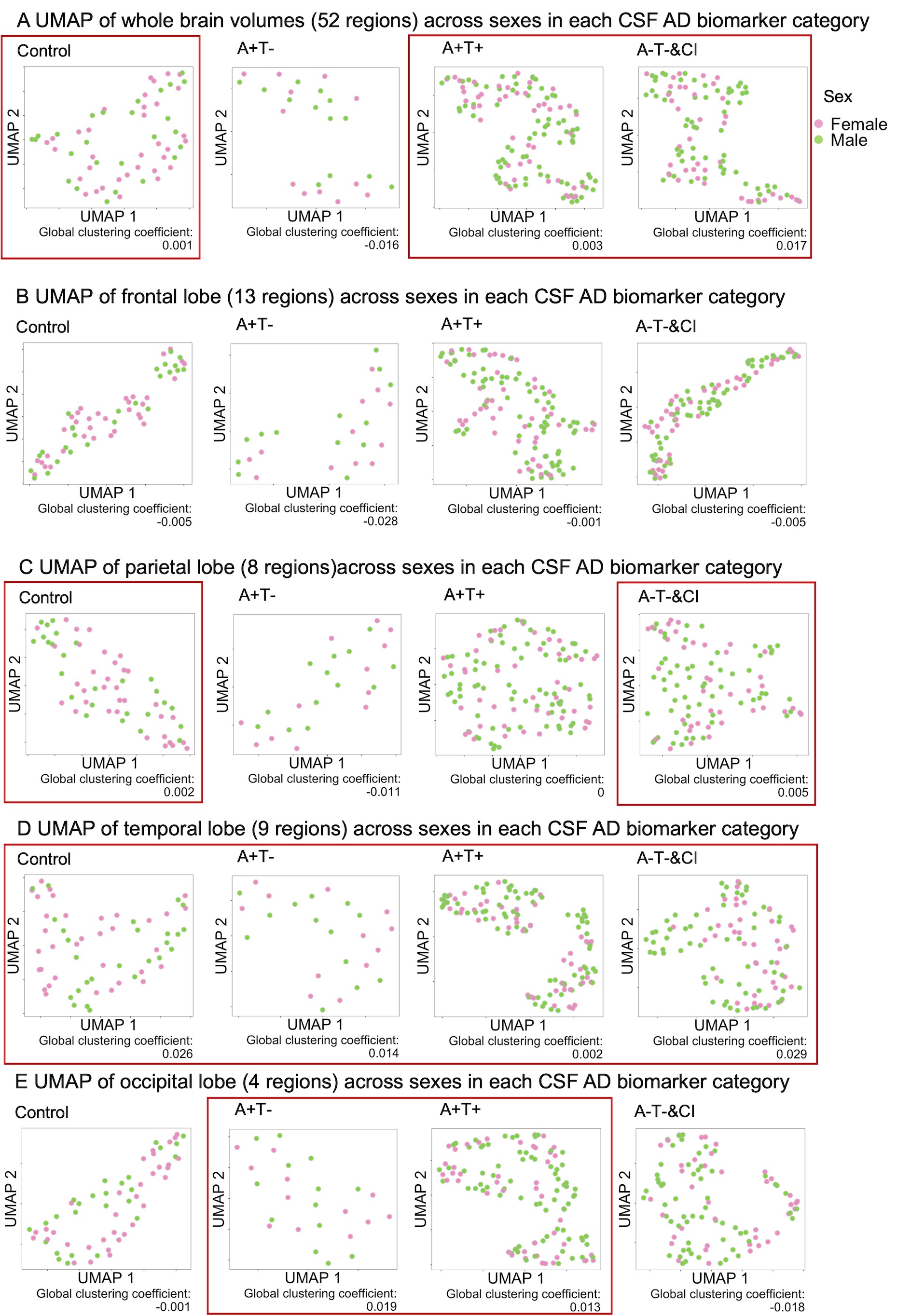
**

**Supplementary Figure 9. Clustering of brain volumes by sex in the whole brain and other subregions.** Control, A+T+, and A-T-&CI showed sex clustering (i.e., positive global clustering coefficient) at the whole brain level. No group showed sex clustering in the frontal lobe. Both control and A-T-&CI showed sex clustering in parietal and all groups showed sex clustering in the temporal lobe. A+T- and A+T+ groups both showed sex clustering in the occipital lobe. Red boxes highlight the brain regions that showed sex clustering. Each data point represents the brain volume of an individual participant projected into the UMAP 2D space. Sample sizes: Control (total: 60; female: 34, male: 26), A+T- (total: 25; female: 12, male: 13), A+T+ (total: 121; female: 46, male: 75), A-T-&CI (total: 100; female: 40, male: 60). A+T-: amyloid positive, tau negative. A+T+: amyloid positive tau positive. A-T- & CI: amyloid negative, tau negative, and cognitive impaired. UMAP: Uniform Manifold Approximation and Projection. AD: Alzheimer’s disease.


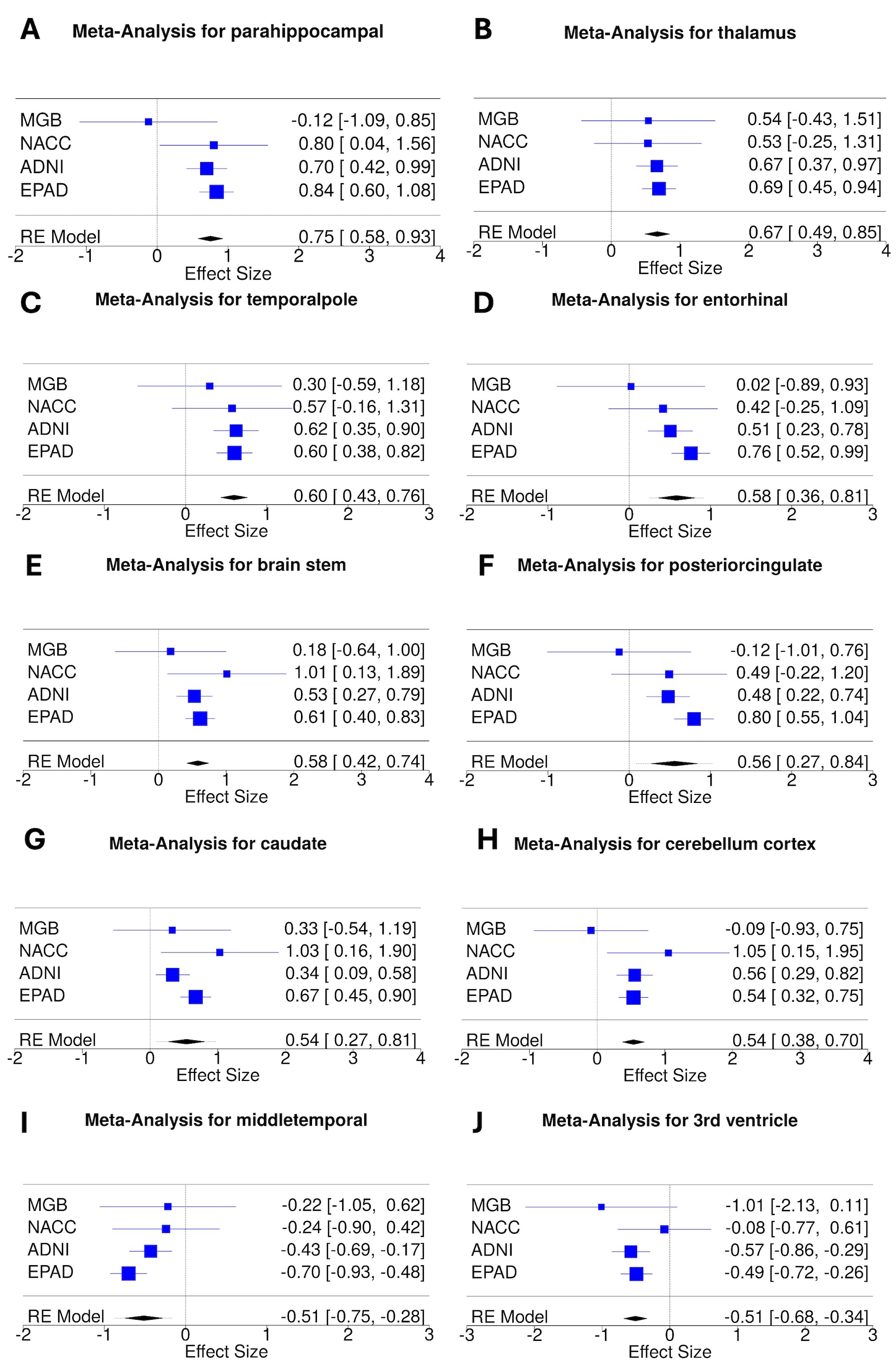


**Supplementary Figure 10.** Forest plot showing the 10 brain areas where brain volumes exhibited significant sex differences in A+T- and had the largest effect sizes from the random-effects model with REML estimation. Each box represents the effect size from a logistic regression analysis in an individual dataset, where the center marks the mean, the box size reflects the sample size, and the horizontal lines indicate the confidence interval. The bottom row in each subplot shows the overall meta-analytic effect size. Sample sizes: MGB (total: 25; female: 12, male: 13), NACC (total: 39; female: 20, male: 19), ADNI (total: 280; female: 122, male: 158), EPAD (total: 432; female: 226, male: 206). RE model: Random-Effects model. REML: Restricted Maximum Likelihood. A+T-: amyloid positive, tau negative. MGB: Mass General Brigham. NACC: National Alzheimer’s Coordinating Center. ADNI: Alzheimer’s Disease Neuroimaging Initiative. EPAD: European Prevention of Alzheimer’s Dementia.


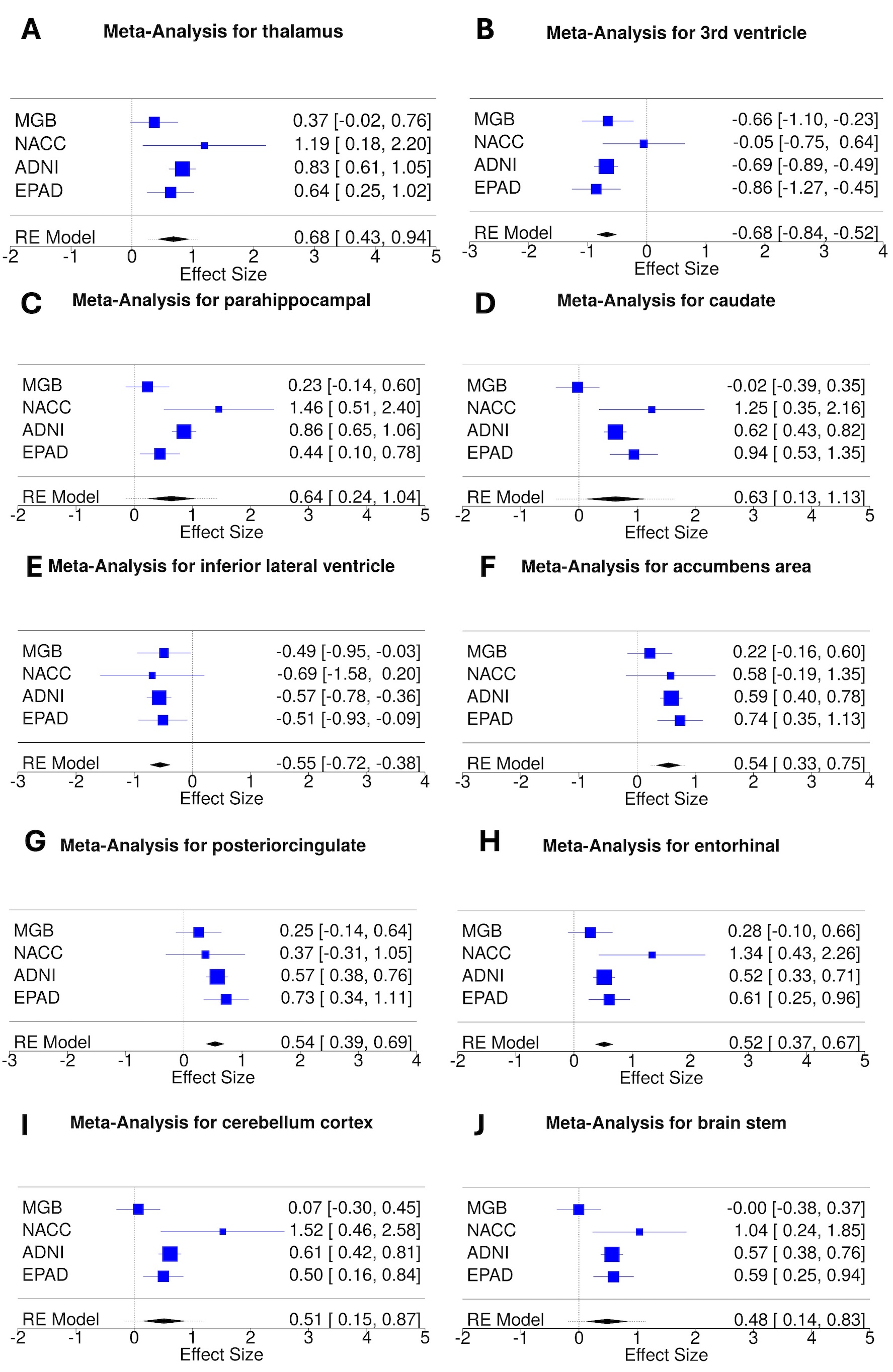


**Supplementary Figure 11.** Forest plots showing the 10 brain areas where brain volumes exhibited significant sex differences in A+T+ and had the largest effect sizes from the random-effects model with REML estimation. Each box represents the effect size from a logistic regression analysis in an individual dataset, where the center marks the mean, the box size reflects the sample size, and the horizontal lines indicate the confidence interval. The bottom row in each subplot shows the overall meta-analytic effect size. Sample sizes: MGB (total: 121; female: 46, male: 75), NACC (total: 42; female: 18, male: 24), ADNI (total: 572; female: 271, male: 301), EPAD (total: 165; female: 84, male: 81). RE model: Random-Effects model. REML: Restricted Maximum Likelihood. A+T+: amyloid positive, tau positive. MGB: Mass General Brigham. NACC: National Alzheimer’s Coordinating Center. ADNI: Alzheimer’s Disease Neuroimaging Initiative. EPAD: European Prevention of Alzheimer’s Dementia.


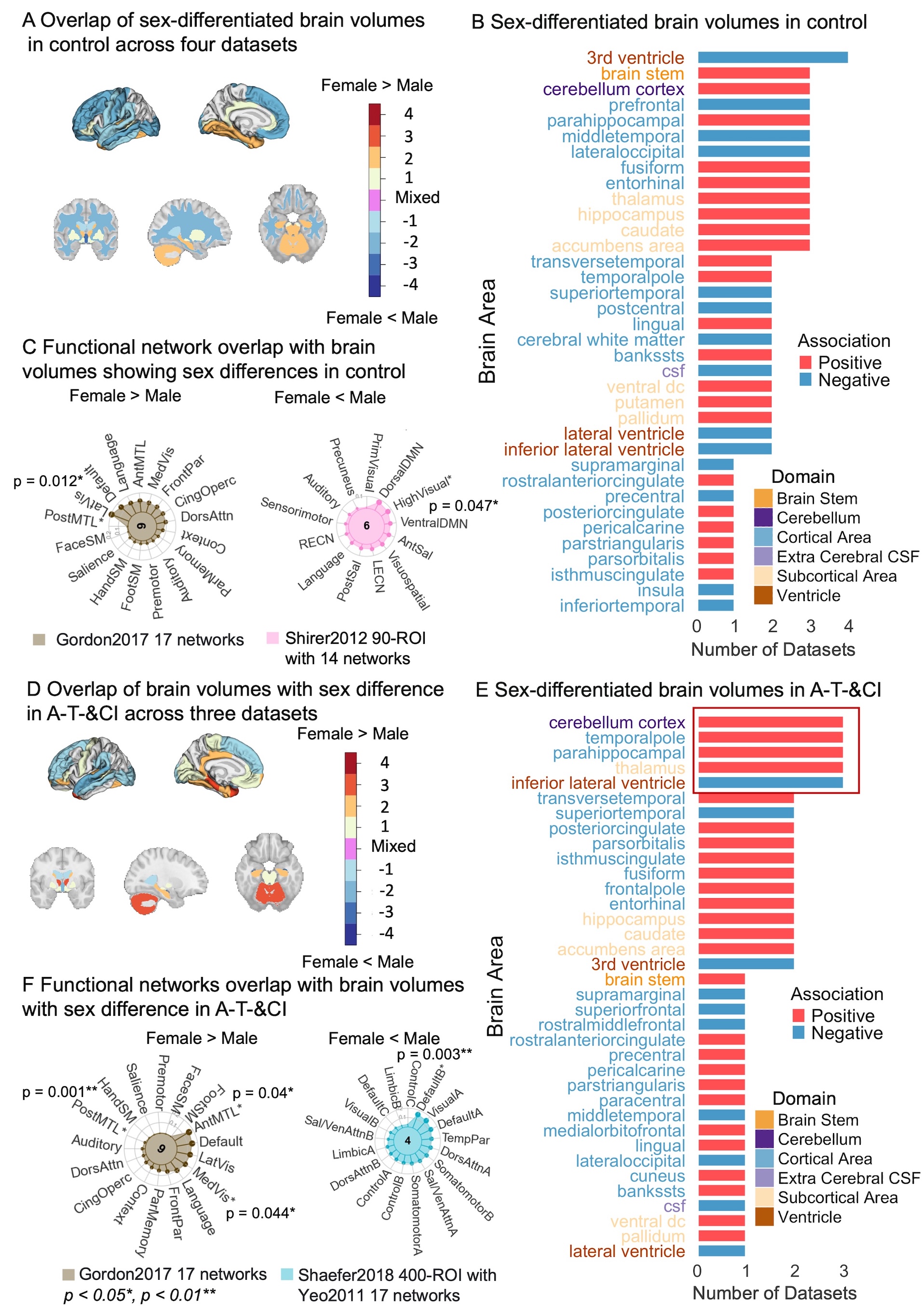


**Supplementary Figure 12. Sex-differentiated brain regions in Control and A-T-&CI (other dementia) groups.** (**A**) Overlap of sex-differentiated brain volumes in control. Visualization of overlapping regions across four datasets. (**B**) Count of sex-differentiated brain volumes in control. Stacked bar plot showing significant brain volumes in individuals aged 50+, colored by association direction (Female > Male or Male > Female). (**C**) Functional network overlap in Control. Spin tests of brain regions significantly associated with sex differences in Control in the meta-analyses and function networks show significant overlap (*ps* < 0.05) with Posterior MTL Network (larger brain volumes in females) and High Visual and Sensory Motor Networks (larger brain volumes in males). (**D**) Overlap of sex-differentiated brain volumes in A-T-&CI. Visualization of overlapping regions across three datasets (NACC dataset excluded due to small sample size). (**E**) Count of sex-differentiated brain volumes in A-T-&CI. Stacked bar plot showing significant regions for individuals aged 50+, with colors indicating association direction (Female > Male or Male > Female). The red box highlights five consistently significant brain areas across all datasets. (**F**) Functional network overlap in A-T-&CI. Spin tests of brain regions significantly associated with sex differences in A-T-&CI in the meta-analyses and function networks reveal significant overlap (*ps* < 0.05) with Anterior/Posterior Medial Temporal Lobe and Medial Visual Networks (larger brain volumes in females) and Default B Network (larger brain volumes in males). In (A) and (D), darker colors indicate greater overlap (numbers show dataset counts per region); purple indicates mixed associations (positive in some datasets, negative in others). In (C) and (F), asterisks (*) indicate networks with significant overlap and the *p value* was based on the spin test permutations of the Dice coefficients. Network abbreviations correspond to the functional networks: DorsAttn/DorsAttnA/B – Dorsal Attention Network A/B; VisualA/B/SVis/PrimaryVisual/HighVisual – Visual Networks A/B, Secondary, Primary, and Higher-Order Visual Networks; Default/DefaultA/B/DorsalDMN/VentralDMN – Default Mode Networks A/B, Dorsal, and Ventral; ControlA/B/C – Frontoparietal Control Networks A/B/C; Sal/Salience/AntSal/PostSal/SalVenAttnA/B – Salience and Ventral Attention Networks; SomatomotorA/B/FaceSM/HandSM/FootSM – Somatomotor Networks A/B and Face, Hand, Foot Somatomotor Regions; CingOperc – Cingulo-Opercular Network; FromPar – Fronto-Parietal Network; MedVis – Medial Visual Network; AntMTL/PostMTL – Anterior/Posterior Medial Temporal Lobe Networks; RECN – Retrosplenial/Extended Cingulate Network; LECN – Left Executive Control Network; TempPar – Temporo-Parietal Network; LimbicA/B – Limbic Networks A/B; Context – Contextual Association Network; Memory – Memory Network; Precuneus – Precuneus Network; Sensorimotor – Sensorimotor Network; Language – Language Network; Premotor – Premotor Network; and Auditory – Auditory Network. A-T- & CI: amyloid negative, tau negative, and cognitive impaired.


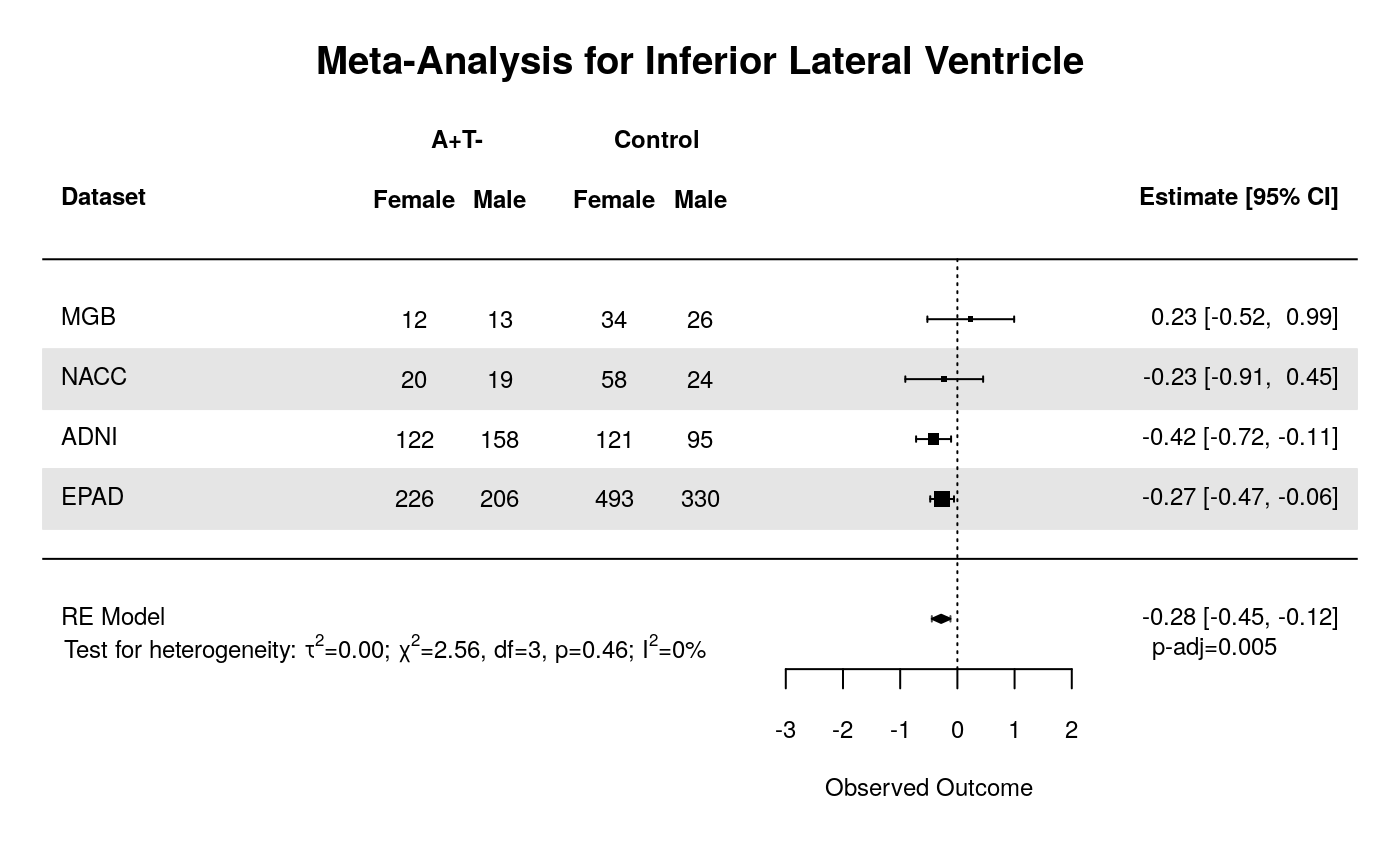


**Supplementary Figure 13.** **Forest plot from the meta-analysis of the inferior lateral ventricle.** It highlights a significant sex difference associated with amyloid across four datasets using a random-effects model with REML estimation. The resulting beta estimate is -0.28 [-0.45, -0.12], with *pFDR* = 0.005, and no observed *heterogeneity* (0%). Values to the left of the forest plot represents sample size of each group. Each box represents the effect size from a logistic regression analysis in an individual dataset, where the center marks the mean, the box size reflects the sample size, and the horizontal lines indicate the confidence interval. The bottom row in each subplot shows the overall meta-analytic effect size. RE model: Random-Effects model. REML: Restricted Maximum Likelihood. CI: confidence interval. A+T-: amyloid positive, tau negative. A+T+: amyloid positive, tau positive. MGB: Mass General Brigham. NACC: National Alzheimer’s Coordinating Center. ADNI: Alzheimer’s Disease Neuroimaging Initiative. EPAD: European Prevention of Alzheimer’s Dementia. p-adj/pFDR: false discovery rate-corrected p value.

## Supplementary Tables

**Supplementary Table 1a. Summary statistics of the demographic characteristics in the NACC dataset.**

| **Characteristics** | **Total (N = 171)** | **Control (N = 82, 48%)** | **A+T- (N = 39, 22.8%)** | **A+T+ (N = 42, 24.6%)** | **A-T- & CI (N = 8, 4.7%)** |
| --- | --- | --- | --- | --- | --- |
| **Age, mean (SD), years** |  |  |  |  |  |
|  | 65.5 (9.7) | 61.8 (8.4) | 68.3 (10.2) | 71.1 (8.7) | 61.8 (7.7) |
| **Sex, N (%)** |  |  |  |  |  |
| Female | 97 (56.7) | 58 (70.7) | 20 (51.3) | 18 (42.9) | 1 (23.5) |
| Male | 74 (43.3) | 24 (29.3) | 19 (48.7) | 24 (57.1) | 7 (87.5) |
| **Race, N (%)** |  |  |  |  |  |
| White | 169 (98.8) | 82 (100.0) | 37 (94.9) | 42 (100.0) | 8 (100.0) |
| Black or AA | 2 (1.2) | 0 (0.0) | 2 (5.1) | 0 (0.0) | 0 (0.0) |
| **Ethnicity, N (%)** |  |  |  |  |  |
| Not Hispanic or Latino | 171 (100) | 82 (100) | 39 (100) | 42 (100) | 8 (100) |
| **Education, N (%)** |  |  |  |  |  |
| ≤ 12 years | 29 (17.0) | 8 (9.8) | 9 (23.1) | 9 (21.4) | 3 (37.5) |
| 13 – 16 years | 75 (43.9) | 41 (50.0) | 12 (30.8) | 20 (47.6) | 2 (25.0) |
| 17+ years | 67 (39.2) | 33 (40.2) | 18 (46.2) | 13 (31.0) | 3 (37.5) |

The mean age of this cohort was 65.5 ± 9.7 years old, and 56.7% were women. A majority of the cohort identify as White (98.8%) and all were not Hispanic or Latino (100%). 17% of the participants had 12 years or fewer of education, 43.9% had 13-16 years of education, and 39.2% had attained 17 years or more of education. In terms of CSF Alzheimer’s disease biomarker group, 48% (*n* = 82) were control (i.e., A-T- & cognitively unimpaired), 22.8% (*n* = 39) of the patients were A+T-, 24.6% (*n* = 42) were A+T+, and 4.7% (*n* = 8) were A-T-&CI. AA: African American. A+T-: amyloid positive, tau negative. A+T+: amyloid positive tau positive. A-T- & CI: amyloid negative, tau negative, and cognitive impaired. NACC: National Alzheimer’s Coordinating Center.

**Supplementary Table 1b. Summary statistics of the demographic characteristics in the ADNI dataset.**

| **Characteristics** | **Total (N = 1,363)** | **Control (N = 216, 15.8%)** | **A+T- (N = 280, 20.5%)** | **A+T+ (N = 572, 42%)** | **A-T- & CI (N = 295, 21.6%)** |
| --- | --- | --- | --- | --- | --- |
| **Age, mean (SD), years** |  |  |  |  |  |
|  | 72.8 (7.3) | 70.3 (6) | 73.3 (6.8) | 74.1 (7.3) | 71.6 (8) |
| **Sex, N (%)** |  |  |  |  |  |
| Female | 638 (46.8) | 121 (56.0) | 122 (43.6) | 271 (47.4) | 124 (42.0) |
| Male | 725 (53.2) | 95 (44.0) | 158 (56.4) | 301 (52.6) | 171 (58.0) |
| **Race, N (%)** |  |  |  |  |  |
| White | 1266 (92.9) | 189 (87.5) | 257 (91.8) | 551 (96.3) | 269 (91.2) |
| Black or AA | 54 (4.0) | 17 (7.9) | 12 (4.3) | 14 (2.4) | 11 (3.7) |
| Asian | 20 (1.5) | 5 (2.3) | 4 (1.4) | 4 (0.7) | 7 (2.4) |
| AI or AN | 2 (0.1) | 0 (0.0) | 0 (0.0) | 0 (0.0) | 2 (0.7) |
| NH and Other PI | 2 (0.1) | 0 (0.0) | 1 (0.4) | 0 (0.0) | 1 (0.3) |
| More Than One Race | 17 (1.2) | 5 (2.3) | 6 (2.1) | 2 (0.3) | 4 (1.4) |
| Not Available | 2 (0.1) | 0 (0.0) | 0 (0.0) | 1 (0.2) | 1 (0.3) |
| **Ethnicity, N (%)** |  |  |  |  |  |
| Not Hispanic or Latino | 1313 (96.3) | 202 (93.5) | 271 (96.8) | 555 (97.0) | 285 (96.6) |
| Hispanic or Latino | 43 (3.2) | 13 (6.0) | 4 (1.4) | 16 (2.8) | 10 (3.4) |
| Not Available | 7 (0.5) | 1 (0.5) | 5 (1.8) | 1 (0.2) | 0 (0.0) |
| **Education, N (%)** |  |  |  |  |  |
| ≤ 12 years | 194 (14.2) | 21 (9.7) | 42 (15.0) | 95 (16.6) | 36 (12.2) |
| 13 – 16 years | 604 (44.3) | 89 (41.2) | 117 (41.8) | 270 (47.2) | 128 (43.4) |
| 17+ years | 565 (41.5) | 106 (49.1) | 121 (43.2) | 207 (36.2) | 131 (44.4) |

The mean age of this cohort was 72.8 ± 7.3 years old, and 46.8% were women. A majority of the cohort identify as White (92.9%) and all were not Hispanic or Latino (96.3%). 14.2% of the participants had 12 years or fewer of education, 44.3% had 13-16 years of education, and 41.5% had attained 17 years or more of education. In terms of CSF Alzheimer’s disease biomarker group, 15.8% (*n* = 216) were control (i.e., A-T- & cognitively unimpaired), 20.5% (*n* = 280) of the patients were A+T-, 42% (*n* = 572) were A+T+, and 21.6% (*n* = 295) were A-T-&CI. AA: African American; AI: American Indian; AN: Alaska Native; NH: Native Hawaiian; PI: Pacific Islander. A+T-: amyloid positive, tau negative. A+T+: amyloid positive tau positive. A-T- & CI: amyloid negative, tau negative, and cognitive impaired. ADNI: Alzheimer’s Disease Neuroimaging Initiative.

**Supplementary Table 1c. Summary statistics of the demographic characteristics in the EPAD dataset.**

| **Characteristics** | **Total (N = 1,603)** | **Control (N = 823, 51.3%)** | **A+T- (N = 432, 26.9%)** | **A+T+ (N = 165, 10.3%)** | **A-T- & CI (N = 183, 11.4%)** |
| --- | --- | --- | --- | --- | --- |
| **Age, mean (SD), years** |  |  |  |  |  |
|  | 65.6 (7.4) | 64 (7) | 66 (7.6) | 70.9 (6.4) | 66.7 (7.3) |
| **Sex, N (%)** |  |  |  |  |  |
| Female | 888 (55.4) | 493 (59.1) | 226 (52.3) | 84 (50.9) | 85 (46.4) |
| Male | 715 (44.6) | 330 (40.1) | 206 (47.7) | 81 (49.1) | 98 (53.6) |
| **Race and Ethnicity*,**  **N (%)** |  |  |  |  |  |
| White | 1234 (77.0) | 681 (82.7) | 360 (83.3) | 116 (70.3) | 77 (42.1) |
| Black | 2 (0.1) | 1 (0.1) | 0 (0.0) | 1 (0.6) | 0 (0.0) |
| Asian | 8 (0.5) | 1 (0.1) | 3 (0.7) | 2 (1.2) | 2 (1.1) |
| Hispanic or Latino | 12 (0.7) | 6 (0.7) | 3 (0.7) | 1 (0.6) | 2 (1.1) |
| Other | 3 (0.2) | 3 (0.4) | 0 (0.0) | 0 (0.0) | 0 (0.0) |
| Not Available | 344 (21.5) | 131 (15.9) | 66 (15.3) | 45 (27.3) | 102 (55.7) |
| **Education, N (%)** |  |  |  |  |  |
| ≤ 12 years | 543 (33.9) | 253 (30.7) | 148 (34.3) | 70 (42.4) | 72 (39.3) |
| 13 – 16 years | 529 (33.0) | 293 (35.6) | 136 (31.5) | 51 (30.9) | 49 (26.8) |
| 17+ years | 531 (33.1) | 277 (33.7) | 148 (34.3) | 44 (26.7) | 62 (33.9) |

The mean age of this cohort was 65.6 ± 7.4 years old, and 55.4% were women. A majority of the cohort identify as White (77%) and 0.7% reported as Hispanic or Latino. 33.9% of the participants had 12 years or fewer of education, 33% had 13-16 years of education, and 33.1% had attained 17 years or more of education. In terms of CSF Alzheimer’s disease biomarker group, 51.3% (*n* = 823) were control (i.e., A-T- & cognitively unimpaired), 26.9% (*n* = 432) of the patients were A+T-, 10.3% (*n* = 165) were A+T+, and 11.4% (*n* = 183) were A-T-&CI. A+T-: amyloid positive, tau negative. A+T+: amyloid positive tau positive. A-T- & CI: amyloid negative, tau negative, and cognitive impaired. EPAD: European Prevention of Alzheimer’s Dementia. *Note that in the EPAD dataset, race and ethnicity were combined into one category; in France, the collection of ethnicity data is prohibited by law, therefore this information is not available for individuals at French sites.

**Supplementary Table 1d. Summary statistics of the clinical characteristics of the A-T-&CI (other dementia) group in the MGB dataset.**

| **Neurocognitive Category, N (%)** | **A-T-&CI (n = 100; 100%)** |
| --- | --- |
| **AD** |  |
| MCI AD | 3 (3.0) |
| Dementia AD | 0 (0.0) |
| **Mixed** |  |
| MCI AD Mixed | 2 (2.0) |
| Dementia AD Mixed | 1 (1.0) |
| **NPH** |  |
| MCI NPH | 17 (17.0) |
| Dementia NPH | 2 (2.0) |
| **Other** |  |
| MCI Other Neurodegeneration | 10 (10.0) |
| MCI Other/NOS | 49 (49.0) |
| Dementia Other/NOS | 9 (9.0) |
| Dementia Other Neurodegeneration | 7 (7.0) |

In the A-T-&CI group, only 6 percent (*n* = 6) of patients were clinically categorized as AD or mixed AD. NOS: not otherwise specified. NPH: normal pressure hydrocephalus. A-T- & CI: amyloid negative, tau negative, and cognitive impaired. AD: Alzheimer’s disease. MCI: mild cognitive impairment. MGB: Mass General Brigham.

**Supplementary Table 2. meta-analyses results of brain volumes associated with amyloid status.**

| **Feature** | **MGB beta** | **NACC beta** | **ADNI beta** | **EPAD beta** | **Meta beta** | **p value** | **p adjusted** |
| --- | --- | --- | --- | --- | --- | --- | --- |
| inferior lateral ventricle | 0.375 | 0.915 | 1.227 | 0.699 | 0.831 [0.472, 1.19] | **< .001** | **< .001** |
| lateral ventricle | 0.449 | 0.942 | 0.742 | 0.658 | 0.685 [0.561, 0.809] | **< .001** | **< .001** |
| amygdala | -0.869 | -0.967 | -0.567 | -0.458 | -0.554 [-0.713, -0.395] | **< .001** | **< .001** |
| 3^rd^ ventricle | 0.295 | 0.714 | 0.413 | 0.529 | 0.5 [0.384, 0.615] | **< .001** | **< .001** |
| accumbens area | -0.377 | -1.111 | -0.25 | -0.501 | -0.498 [-0.799, -0.196] | **0.001** | **0.003** |
| hippocampus | -0.463 | -0.3 | -0.664 | -0.394 | -0.479 [-0.662, -0.296] | **< .001** | **< .001** |
| thalamus | -0.885 | -0.859 | -0.512 | -0.389 | -0.474 [-0.625, -0.323] | **< .001** | **< .001** |
| pallidum | -0.635 | -1.16 | -0.089 | -0.042 | -0.406 [-0.901, 0.089] | 0.108 | 0.176 |
| inferiortemporal | -0.409 | -1.013 | -0.344 | -0.092 | -0.394 [-0.748, -0.04] | **0.029** | 0.075 |
| cerebral white matter | 0.01 | -1.175 | -0.384 | -0.139 | -0.393 [-0.825, 0.04] | 0.075 | 0.133 |
| inferiorparietal | -0.335 | -0.92 | -0.336 | -0.068 | -0.35 [-0.676, -0.023] | 0.036 | 0.088 |
| bankssts | -0.504 | -0.902 | -0.261 | 0.078 | -0.331 [-0.735, 0.073] | 0.108 | 0.176 |
| middletemporal | -0.274 | -0.835 | -0.414 | 0.012 | -0.331 [-0.675, 0.013] | 0.06 | 0.123 |
| precuneus | -0.276 | -0.883 | -0.246 | -0.283 | -0.296 [-0.397, -0.196] | **< .001** | **< .001** |
| lingual | -0.222 | -0.792 | -0.062 | -0.216 | -0.27 [-0.542, 0.002] | 0.052 | 0.119 |
| ventral dc | -0.888 | -0.04 | -0.352 | -0.214 | -0.264 [-0.387, -0.142] | **< .001** | **< .001** |
| rostralanteriorcingulate | -0.195 | -1.07 | 0.013 | -0.042 | -0.263 [-0.706, 0.18] | 0.244 | 0.307 |
| supramarginal | -0.229 | -0.748 | -0.276 | -0.024 | -0.257 [-0.536, 0.021] | 0.07 | 0.132 |
| caudalmiddlefrontal | 0.101 | -0.647 | -0.435 | -0.018 | -0.245 [-0.557, 0.067] | 0.124 | 0.193 |
| fusiform | -0.265 | -0.408 | -0.298 | -0.192 | -0.232 [-0.333, -0.131] | **< .001** | **< .001** |
| posteriorcingulate | 0.326 | -0.807 | -0.046 | -0.285 | -0.208 [-0.59, 0.173] | 0.285 | 0.337 |
| entorhinal | 0.148 | -0.86 | 0.022 | -0.165 | -0.192 [-0.561, 0.177] | 0.309 | 0.354 |
| putamen | -0.712 | -0.672 | -0.185 | -0.149 | -0.189 [-0.294, -0.084] | **< .001** | **< .001** |
| lateraloccipital | -0.137 | -0.766 | 0.057 | -0.114 | -0.184 [-0.487, 0.119] | 0.234 | 0.304 |
| pericalcarine | -0.091 | -0.417 | -0.046 | -0.213 | -0.171 [-0.302, -0.039] | **0.011** | **0.031** |
| superiorparietal | -0.072 | -0.385 | -0.145 | -0.163 | -0.166 [-0.263, -0.069] | **0.001** | **0.003** |
| caudate | -0.548 | -0.428 | 0.085 | -0.145 | -0.162 [-0.41, 0.086] | 0.2 | 0.279 |
| caudalanteriorcingulate | 0.296 | -0.931 | -0.07 | 0.022 | -0.155 [-0.607, 0.296] | 0.499 | 0.541 |
| cerebellum cortex | -0.892 | -0.335 | -0.162 | -0.119 | -0.154 [-0.255, -0.054] | **0.003** | **0.009** |
| transversetemporal | -0.002 | -0.807 | 0.055 | 0.032 | -0.137 [-0.499, 0.224] | 0.457 | 0.509 |
| medialorbitofrontal | -0.427 | -0.395 | 0.079 | -0.156 | -0.131 [-0.341, 0.078] | 0.219 | 0.295 |
| prefrontal | 0.36 | -0.389 | -0.302 | -0.026 | -0.13 [-0.358, 0.098] | 0.264 | 0.322 |
| cuneus | 0.105 | -0.462 | 0.009 | -0.181 | -0.124 [-0.291, 0.042] | 0.143 | 0.214 |
| parahippocampal | 0.151 | -0.587 | -0.062 | -0.096 | -0.101 [-0.204, 0.003] | 0.056 | 0.121 |
| brain stem | -0.641 | 0.303 | -0.131 | -0.079 | -0.09 [-0.188, 0.008] | 0.071 | 0.132 |
| cerebellum white matter | -1.201 | 0.101 | -0.111 | -0.039 | -0.065 [-0.162, 0.032] | 0.191 | 0.276 |
| paracentral | 0.423 | 0.11 | -0.311 | 0.031 | -0.024 [-0.283, 0.234] | 0.855 | 0.901 |
| csf | -0.038 | 0.606 | 0.002 | -0.153 | 0.017 [-0.225, 0.259] | 0.889 | 0.912 |
| 4^th^ ventricle | -0.51 | -0.007 | -0.091 | 0.269 | -0.003 [-0.282, 0.276] | 0.983 | 0.983 |

15 brain regions showed significant association with amyloid (p values bolded) in the meta-analyses and 14 regions showed significance after multiple comparison, with false discovery rate (*FDR*) adjusted p values bolded. MGB: Mass General Brigham. NACC: National Alzheimer’s Coordinating Center. ADNI: Alzheimer’s Disease Neuroimaging Initiative. EPAD: European Prevention of Alzheimer’s Dementia.

**Supplementary Table 3. meta-analyses results of brain volumes associated with tau status in the presence of amyloid.**

| **Feature** | **MGB**  **beta** | **NACC**  **beta** | **ADNI**  **beta** | **EPAD**  **beta** | **Meta beta** | **p value** | **p adjusted** |
| --- | --- | --- | --- | --- | --- | --- | --- |
| lateral ventricle | -0.581 | -0.166 | -0.393 | -0.546 | -0.427 [-0.548, -0.305] | **< .001** | **< .001** |
| csf | 0.694 | 0.23 | 0.326 | 0.522 | 0.418 [0.259, 0.577] | **< .001** | **< .001** |
| middletemporal | -0.432 | -0.06 | -0.277 | -0.509 | -0.343 [-0.512, -0.175] | **< .001** | **< .001** |
| 3rd ventricle | -0.398 | 0.01 | -0.355 | -0.344 | -0.331 [-0.453, -0.208] | **< .001** | **< .001** |
| 4th ventricle | -0.368 | -0.28 | -0.229 | -0.287 | -0.256 [-0.367, -0.144] | **< .001** | **< .001** |
| hippocampus | -0.453 | -0.842 | -0.093 | -0.005 | -0.234 [-0.537, 0.069] | 0.131 | 0.196 |
| cuneus | 0.07 | 0.312 | 0.235 | 0.205 | 0.219 [0.108, 0.33] | **< .001** | **< .001** |
| pericalcarine | 0.179 | -0.065 | 0.21 | 0.294 | 0.219 [0.109, 0.33] | **< .001** | **< .001** |
| bankssts | -0.383 | -0.169 | -0.146 | -0.253 | -0.194 [-0.304, -0.083] | **0.001** | **0.002** |
| superiortemporal | 0.058 | -0.037 | -0.227 | -0.188 | -0.187 [-0.299, -0.076] | **0.001** | **0.002** |
| lingual | 0.173 | 0.166 | 0.192 | 0.182 | 0.186 [0.075, 0.297] | **0.001** | **0.002** |
| precentral | 0.223 | 0.26 | 0.146 | 0.235 | 0.186 [0.075, 0.297] | **0.001** | **0.002** |
| postcentral | -0.001 | 0.671 | 0.153 | 0.135 | 0.158 [0.045, 0.27] | **0.006** | **0.012** |
| inferior lateral ventricle | -0.335 | -0.005 | -0.172 | -0.104 | -0.151 [-0.263, -0.039] | **0.008** | **0.015** |
| caudate | 0.159 | -0.441 | -0.266 | -0.031 | -0.145 [-0.34, 0.049] | 0.143 | 0.202 |
| inferiorparietal | -0.215 | 0.133 | 0.019 | -0.377 | -0.128 [-0.37, 0.114] | 0.3 | 0.36 |
| medialorbitofrontal | 0.012 | 0.089 | 0.089 | 0.218 | 0.123 [0.012, 0.235] | **0.03** | 0.051 |
| paracentral | 0.101 | 0.087 | 0.22 | -0.02 | 0.11 [-0.051, 0.272] | 0.18 | 0.24 |
| parstriangularis | -0.199 | 0.034 | -0.209 | 0.036 | -0.1 [-0.268, 0.068] | 0.242 | 0.306 |
| superiorparietal | -0.194 | -0.028 | 0.148 | 0.072 | 0.096 [-0.013, 0.205] | 0.085 | 0.136 |
| caudalmiddlefrontal | 0.037 | 0.094 | 0.166 | -0.049 | 0.073 [-0.074, 0.22] | 0.329 | 0.376 |
| insula | -0.043 | 0.106 | -0.187 | 0.062 | -0.053 [-0.227, 0.121] | 0.553 | 0.603 |
| brain stem | -0.055 | -0.65 | 0.037 | 0.121 | -0.035 [-0.264, 0.195] | 0.767 | 0.8 |
| ventral dc | 0.067 | -0.597 | -0.014 | 0.097 | 0.006 [-0.108, 0.12] | 0.919 | 0.919 |

14 brain regions showed significant association with tau in the presence of amyloid (p values bolded) in the meta-analyses and 13 regions showed significance after multiple comparison, with false discovery rate (*FDR*) adjusted p values bolded. MGB: Mass General Brigham. NACC: National Alzheimer’s Coordinating Center. ADNI: Alzheimer’s Disease Neuroimaging Initiative. EPAD: European Prevention of Alzheimer’s Dementia.

**Supplementary Table 4a. Global network metrics of sex differences in brain volumes across CSF biomarker groups.**

| **Group (N)** | **Global clustering coefficients** | **Path length** | **Global efficiency** |  |
| --- | --- | --- | --- | --- |
|  |  |  |  |  |
|  |  |  |  |  |
| **Control (N=60)** |  |  |  |  |
| Female (N = 34) | 0.655 | 1.879 | 0.634 |  |
| Male (N = 26) | 0.667 | 1.885 | 0.633 |  |
| **A+T- (N = 25）** |  |  |  |  |
| Female (N = 12) | 0.534 | 1.731 | 0.657 |  |
| Male (N = 13) | 0.567 | 1.72 | 0.659 |  |
| **A+T+ (N = 121)** |  |  |  |  |
| Female (N = 46) | 0.625 | 1.756 | 0.653 |  |
| Male (N = 75) | 0.596 | 1.722 | 0.659 |  |
| **A-T-&CI (N = 100）** |  |  |  |  |
| Female (N = 40) | 0.618 | 1.698 | 0.663 |  |
| Male (N = 60) | 0.605 | 1.693 | 0.664 |  |

**Supplementary Table 4b. Local network metrics and small-worldness of sex differences in brain volumes across each CSF biomarker group.**

| **Group (N)** | **Nodal degree (Mean±SD)** | | **Nodal clustering coefficients (Mean±SD)** | | **Nodal efficiency (Mean±SD)** | | **Small-worldness (Mean±SD)** | |
| --- | --- | --- | --- | --- | --- | --- | --- | --- |
|  |  |  |  |  |  |  |  |  |
|  |  |  |  |  |  |  |  |  |
| **Control (N=60)** |  |  |  |  |  |  |  |  |
| Female (N = 34) | 17.23±9.65 | p = 0.975 | 0.655±0.185 | p = 0.677 | 0.773±0.294 | p = 0.458 | 0.348±0.098 | p = 0.695 |
| Male (N = 26) | 17.23±9.61 |  | 0.667±0.158 |  | 0.796±0.158 |  | 0.354±0.084 |  |
| **A+T- (N = 25）** |  |  |  |  |  |  |  |  |
| Female (N = 12) | 17.23±6.69 | p = 0.975 | 0.534±0.153 | p = 0.931 | 0.709±0.193 | p = 0.458 | 0.309±0.088 | p = 0.816 |
| Male (N = 13) | 17.23±7.02 |  | 0.567±0.11 |  | 0.765±0.091 |  | 0.329±0.064 |  |
| **A+T+ (N = 121)** |  |  |  |  |  |  |  |  |
| Female (N = 46) | 17.23±7.86 | p = 0.975 | 0.625±0.157 | p = 0.674 | 0.787±0.148 | p = 0.458 | 0.356±0.09 | p=0.695 |
| Male (N = 75) | 17.23±7.06 |  | 0.596±0.135 |  | 0.779±0.089 |  | 0.346±0.079 |  |
| **A-T-&CI (N = 100）** |  |  |  |  |  |  |  |  |
| Female (N = 40) | 17.23±5.34 | p = 0.975 | 0.618±0.126 | p = 0.674 | 0.795±0.091 | p = 0.458 | 0.364±0.074 | p = 0.695 |
| Male (N = 60) | 17.23±5.83 |  | 0.605±0.122 |  | 0.788±0.077 |  | 0.357±0.072 |  |

The network analysis did not reveal sex differences in any groups with different CSF biomarker categories. A+T-: amyloid positive, tau negative. A+T+: amyloid positive tau positive. A-T- & CI: amyloid negative, tau negative, and cognitive impaired. SD: standard deviation.

**Supplementary Table 5. meta-analyses results of sex-differentiated brain volumes in A+T-.**

| **Feature** | **MGB beta** | **NACC beta** | **ADNI beta** | **EPAD beta** | **Meta beta** | **p value** | **p adjusted** |
| --- | --- | --- | --- | --- | --- | --- | --- |
| parahippocampal | -0.122 | 0.801 | 0.702 | 0.84 | 0.754 [0.579, 0.929] | **< .001** | **< .001** |
| thalamus | 0.54 | 0.533 | 0.666 | 0.695 | 0.67 [0.489, 0.851] | **< .001** | **< .001** |
| temporalpole | 0.297 | 0.572 | 0.622 | 0.602 | 0.597 [0.433, 0.762] | **< .001** | **< .001** |
| entorhinal | 0.022 | 0.418 | 0.507 | 0.758 | 0.584 [0.356, 0.811] | **< .001** | **< .001** |
| brain stem | 0.179 | 1.01 | 0.532 | 0.615 | 0.58 [0.419, 0.742] | **< .001** | **< .001** |
| posteriorcingulate | -0.124 | 0.492 | 0.48 | 0.798 | 0.557 [0.274, 0.841] | **< .001** | **< .001** |
| caudate | 0.326 | 1.03 | 0.335 | 0.674 | 0.538 [0.268, 0.808] | **< .001** | **< .001** |
| cerebellum cortex | -0.09 | 1.051 | 0.555 | 0.537 | 0.537 [0.375, 0.699] | **< .001** | **< .001** |
| parsorbitalis | 1.266 | 0.761 | 0.203 | 0.609 | 0.521 [0.181, 0.862] | **0.003** | **0.004** |
| middletemporal | -0.218 | -0.241 | -0.43 | -0.702 | -0.514 [-0.747, -0.281] | **< .001** | **< .001** |
| 3rd ventricle | -1.011 | -0.08 | -0.573 | -0.491 | -0.508 [-0.679, -0.337] | **< .001** | **< .001** |
| superiortemporal | -0.056 | -1.282 | -0.363 | -0.592 | -0.496 [-0.71, -0.281] | **< .001** | **< .001** |
| superiorfrontal | -0.012 | -1.239 | -0.298 | -0.62 | -0.478 [-0.77, -0.185] | **0.001** | **0.002** |
| accumbens area | -0.033 | 1.144 | 0.481 | 0.37 | 0.422 [0.258, 0.586] | **< .001** | **< .001** |
| inferior lateral ventricle | -0.154 | -0.131 | -0.542 | -0.389 | -0.417 [-0.603, -0.231] | **< .001** | **< .001** |
| fusiform | 0.258 | 0.116 | 0.445 | 0.432 | 0.415 [0.259, 0.571] | **< .001** | **< .001** |
| lateraloccipital | -0.362 | -1.106 | -0.147 | -0.505 | -0.413 [-0.725, -0.101] | **0.009** | **0.011** |
| supramarginal | -0.062 | -0.858 | -0.416 | -0.373 | -0.396 [-0.552, -0.24] | **< .001** | **< .001** |
| transversetemporal | 0.484 | 0.204 | 0.41 | 0.399 | 0.394 [0.243, 0.546] | **< .001** | **< .001** |
| isthmuscingulate | 0.145 | 0.041 | 0.377 | 0.454 | 0.393 [0.237, 0.549] | **< .001** | **< .001** |
| insula | 0.051 | -0.787 | -0.189 | -0.528 | -0.373 [-0.645, -0.102] | **0.007** | **0.009** |
| hippocampus | 0.273 | 0.516 | 0.26 | 0.439 | 0.368 [0.212, 0.524] | **< .001** | **< .001** |
| postcentral | 0.059 | -0.806 | -0.178 | -0.544 | -0.367 [-0.657, -0.078] | **0.013** | **0.015** |
| lingual | -0.031 | -0.032 | 0.444 | 0.351 | 0.352 [0.198, 0.506] | **< .001** | **< .001** |
| prefrontal | 0.296 | -0.64 | -0.361 | -0.314 | -0.321 [-0.476, -0.166] | **< .001** | **< .001** |
| cerebellum white matter | -0.639 | 0.226 | 0.449 | 0.277 | 0.317 [0.167, 0.468] | **< .001** | **< .001** |
| csf | -0.031 | -0.245 | -0.187 | -0.391 | -0.288 [-0.46, -0.115] | **0.001** | **0.002** |
| precentral | 0.134 | -0.65 | -0.123 | -0.436 | -0.285 [-0.538, -0.033] | **0.027** | **0.029** |
| pericalcarine | 0.519 | -0.1 | 0.383 | 0.235 | 0.274 [0.124, 0.423] | **< .001** | **< .001** |
| bankssts | -0.044 | 1.136 | 0.281 | 0.233 | 0.27 [0.119, 0.42] | **< .001** | **< .001** |
| caudalanteriorcingulate | 0.009 | 0.015 | 0.179 | 0.405 | 0.265 [0.067, 0.463] | **0.009** | **0.011** |
| rostralanteriorcingulate | 0.289 | 0.03 | 0.186 | 0.31 | 0.247 [0.094, 0.4] | **0.002** | **0.003** |
| lateral ventricle | -0.231 | -0.088 | -0.426 | -0.131 | -0.244 [-0.468, -0.019] | **0.033** | **0.035** |
| inferiorparietal | -0.499 | -0.121 | -0.139 | -0.267 | -0.217 [-0.369, -0.064] | **0.005** | **0.007** |
| parstriangularis | 0.673 | 0.662 | 0.093 | 0.212 | 0.191 [0.04, 0.343] | **0.013** | **0.015** |
| cerebral white matter | 0.125 | -0.729 | -0.079 | -0.234 | -0.182 [-0.339, -0.024] | **0.024** | **0.027** |
| pallidum | -0.481 | 0.582 | -0.036 | 0.237 | 0.116 [-0.134, 0.366] | 0.363 | 0.373 |
| superiorparietal | 0.413 | -0.883 | 0.078 | -0.031 | -0.009 [-0.154, 0.137] | 0.909 | 0.909 |

36 brain regions showed significant association with sex in A+T- (p values bolded) in the meta-analyses and all of these regions showed significance after multiple comparison, with false discovery rate (*FDR*) adjusted p values bolded. MGB: Mass General Brigham. NACC: National Alzheimer’s Coordinating Center. ADNI: Alzheimer’s Disease Neuroimaging Initiative. EPAD: European Prevention of Alzheimer’s Dementia. A+T-: amyloid positive, tau negative.

**Supplementary Table 6. meta-analyses results of sex-differentiated brain volumes in A+T+.**

| **Feature** | **MGB**  **beta** | **NACC**  **beta** | **ADNI**  **beta** | **EPAD**  **beta** | **Meta beta** | **p value** | **p adjusted** |
| --- | --- | --- | --- | --- | --- | --- | --- |
| thalamus | 0.367 | 1.191 | 0.829 | 0.636 | 0.683 [0.429, 0.936] | **< .001** | **< .001** |
| 3rd ventricle | -0.661 | -0.052 | -0.691 | -0.856 | -0.678 [-0.839, -0.516] | **< .001** | **< .001** |
| parahippocampal | 0.228 | 1.456 | 0.858 | 0.442 | 0.64 [0.24, 1.04] | **0.002** | **0.003** |
| caudate | -0.022 | 1.251 | 0.623 | 0.944 | 0.63 [0.13, 1.13] | **0.013** | **0.015** |
| inferior lateral ventricle | -0.488 | -0.689 | -0.572 | -0.506 | -0.554 [-0.724, -0.385] | **< .001** | **< .001** |
| accumbens area | 0.22 | 0.577 | 0.587 | 0.74 | 0.539 [0.333, 0.746] | **< .001** | **< .001** |
| posteriorcingulate | 0.254 | 0.37 | 0.571 | 0.726 | 0.537 [0.385, 0.689] | **< .001** | **< .001** |
| entorhinal | 0.281 | 1.343 | 0.519 | 0.607 | 0.52 [0.371, 0.669] | **< .001** | **< .001** |
| cerebellum cortex | 0.071 | 1.52 | 0.613 | 0.502 | 0.512 [0.155, 0.87] | **0.005** | **0.006** |
| brain stem | -0.002 | 1.041 | 0.567 | 0.595 | 0.485 [0.137, 0.832] | **0.006** | **0.007** |
| lateraloccipital | -0.372 | -0.413 | -0.562 | -0.341 | -0.479 [-0.631, -0.326] | **< .001** | **< .001** |
| temporalpole | 0.524 | 0.808 | 0.42 | 0.565 | 0.473 [0.328, 0.618] | **< .001** | **< .001** |
| middletemporal | -0.304 | -0.333 | -0.436 | -0.484 | -0.422 [-0.565, -0.28] | **< .001** | **< .001** |
| lateral ventricle | -0.427 | -0.707 | -0.404 | -0.348 | -0.408 [-0.561, -0.256] | **< .001** | **< .001** |
| medialorbitofrontal | 0.343 | 0.226 | 0.222 | 0.844 | 0.406 [0.103, 0.709] | **0.009** | **0.011** |
| superiortemporal | -0.248 | -0.342 | -0.416 | -0.479 | -0.402 [-0.546, -0.259] | **< .001** | **< .001** |
| transversetemporal | 0.24 | 0.064 | 0.444 | 0.39 | 0.386 [0.243, 0.529] | **< .001** | **< .001** |
| fusiform | 0.171 | 1.063 | 0.39 | 0.416 | 0.383 [0.239, 0.526] | **< .001** | **< .001** |
| cerebellum white matter | 0.029 | 0.641 | 0.525 | 0.383 | 0.381 [0.135, 0.627] | **0.002** | **0.003** |
| bankssts | 0.282 | 1.05 | 0.36 | 0.424 | 0.38 [0.239, 0.521] | **< .001** | **< .001** |
| rostralmiddlefrontal | -0.151 | -0.388 | -0.356 | -0.488 | -0.351 [-0.492, -0.21] | **< .001** | **< .001** |
| csf | -0.051 | -0.545 | -0.36 | -0.52 | -0.345 [-0.503, -0.186] | **< .001** | **< .001** |
| isthmuscingulate | 0.15 | 0.735 | 0.356 | 0.341 | 0.339 [0.196, 0.482] | **< .001** | **< .001** |
| lingual | 0.26 | 0.464 | 0.359 | 0.286 | 0.336 [0.194, 0.478] | **< .001** | **< .001** |
| frontalpole | 0.439 | 0.101 | 0.246 | 0.569 | 0.334 [0.153, 0.515] | **< .001** | **< .001** |
| parsorbitalis | 0.168 | 0.687 | 0.341 | 0.346 | 0.331 [0.189, 0.474] | **< .001** | **< .001** |
| putamen | 0.284 | 0.711 | 0.35 | 0.187 | 0.321 [0.176, 0.466] | **< .001** | **< .001** |
| hippocampus | 0.275 | 0.899 | 0.33 | 0.17 | 0.31 [0.168, 0.452] | **< .001** | **< .001** |
| superiorfrontal | -0.151 | -0.761 | -0.403 | -0.134 | -0.307 [-0.492, -0.122] | **0.001** | **0.002** |
| insula | -0.443 | -1.043 | -0.287 | -0.184 | -0.306 [-0.448, -0.164] | **< .001** | **< .001** |
| caudalmiddlefrontal | 0.64 | 0.033 | 0.251 | 0.367 | 0.294 [0.152, 0.436] | **< .001** | **< .001** |
| caudalanteriorcingulate | 0.313 | 0.061 | 0.295 | 0.24 | 0.275 [0.133, 0.417] | **< .001** | **< .001** |
| pallidum | 0.044 | 0.704 | 0.268 | 0.242 | 0.245 [0.096, 0.394] | **0.001** | **0.002** |
| pericalcarine | 0.185 | 0.18 | 0.223 | 0.281 | 0.226 [0.088, 0.365] | **0.001** | **0.002** |
| postcentral | 0.209 | -0.942 | -0.16 | -0.385 | -0.224 [-0.564, 0.116] | 0.197 | 0.202 |
| inferiortemporal | -0.081 | -0.342 | -0.229 | -0.288 | -0.223 [-0.362, -0.085] | **0.002** | **0.003** |
| prefrontal | 0.016 | -0.658 | -0.276 | -0.126 | -0.214 [-0.363, -0.065] | **0.005** | **0.006** |
| ventral dc | 0.137 | 0.926 | 0.336 | -0.154 | 0.204 [-0.104, 0.512] | 0.194 | 0.202 |
| supramarginal | -0.336 | -0.085 | -0.008 | -0.365 | -0.177 [-0.399, 0.045] | 0.119 | 0.132 |
| parstriangularis | -0.193 | 0.565 | 0.194 | 0.221 | 0.149 [-0.034, 0.332] | 0.111 | 0.126 |
| rostralanteriorcingulate | -0.054 | 0.199 | 0.024 | 0.371 | 0.101 [-0.091, 0.292] | 0.302 | 0.302 |
| 4th ventricle | -0.187 | 1.095 | 0.112 | 0.074 | 0.093 [-0.044, 0.23] | 0.182 | 0.196 |

36 brain regions showed significant association with sex in A+T+ (p values bolded) in the meta-analyses and all of these regions showed significance after multiple comparison, with false discovery rate (*FDR*) adjusted p values bolded. MGB: Mass General Brigham. NACC: National Alzheimer’s Coordinating Center. ADNI: Alzheimer’s Disease Neuroimaging Initiative. EPAD: European Prevention of Alzheimer’s Dementia. A+T+: amyloid positive, tau positive.

## Supplementary Methods

Methods – validation datasets

#### NACC (National Alzheimer's Coordinating Center) dataset

Our initial validation utilized statistical modeling and machine learning techniques on the NACC (National Alzheimer's Coordinating Center) dataset. We examined participants whose CSF samples were analyzed using ELISA kits to determine amyloid and tau statuses, as have used in one previous study^1^. Amyloid status was defined using ATI (Aβ42/t-tau index), calculated as ATI = Aβ42/(240 + 1.18 × t-tau). Participants with ATI (Aβ42/t-tau index) < 1 were classified as A+ (amyloid positive), while those with ATI (Aβ42/t-tau index) ≥ 1 were classified as A- (amyloid negative). Tau status was determined using p-tau 181 concentrations, with values ≥ 61 pg/mL indicating T+ (tau positive) and values < 61 pg/mL indicating T- (tau negative), following the criteria from a prior study. Controls were defined as cognitively unimpaired (CDR® Dementia Staging Instrument with score of 0) with an A-T- (amyloid negative-tau negative) biomarker profile. This analysis used data from 3 ADRCs (Alzheimer’s Disease Research Centers) across 171 unique participants with distinct UDS (uniform data set) visits, conducted between September 2005 and March 2024.

#### ADNI (Alzheimer’s Disease Neuroimaging Initiative) dataset

The second phase of validation used data were from the ADNI (Alzheimer’s Disease Neuroimaging Initiative) database (adni.loni.usc.edu). The ADNI was launched in 2003 as a public-private partnership, led by Principal Investigator Michael W. Weiner, MD. The primary goal of ADNI has been to test whether serial MRI, PET, other biological markers, and clinical and neuropsychological assessment can be combined to measure the progression of MCI (mild cognitive impairment) and early Alzheimer’s disease. For this dataset, all CSF samples evaluated with Roche's Elecsys assays were used. For the ADNI dataset (*n* = 1,363), amyloid status was determined using the ratio of p-tau 181 to Aβ42, with a cutoff value set at 0.025^2–4^. The cutoff for p-tau 181 was established at 26.64 pg/mL^5,6^. Control was defined as cognitively unimpaired, determined by CDR (clinical dementia rating) score of 0, with an A-T- (amyloid negative-tau negative) biomarker profile.

#### EPAD (European Prevent of Alzheimer’s Dementia) dataset

A third phase of validation was conducted using the European Prevent of Alzheimer’s Dementia EPAD LCS data set V.IMI (doi:10.34688/epadlcs_v.imi_20.10.30) on the ADDI (Alzheimer's Disease Data Initiative) AD workbench^7^. EPAD LCS is registered at www.clinicaltrials.gov Identifier: NCT02804789. The EPAD LCS was launched in 2015 as a public private partnership, led by Chief Investigator Professor Craig Ritchie MB BS. The primary research goal of the EPAD LCS is to provide a well-phenotyped probability-spectrum population for developing and continuously improving disease models for AD in individuals without dementia. For this dataset, all CSF samples were evaluated using the Roche Elecsys System. For the EPAD dataset (*n* = 1,603), amyloid status was determined using Aβ42, with levels < 1,000 pg/mL classified as A+ (amyloid positive) and ≥ 1,000 pg/mL as A- (amyloid positive). Tau status was determined based on p-tau 181, with levels > 27 pg/mL indicating T+ (tau positive) and ≤ 27 pg/mL indicating T- (tau negative)^8^. Control was defined as cognitively unimpaired, determined by CDR (clinical dementia rating) score of 0, with an A-T- (amyloid negative-tau negative) biomarker profile.

**References**

1. de Souza LC, Chupin M, Lamari F, et al. CSF tau markers are correlated with hippocampal volume in Alzheimer’s disease. *Neurobiol Aging*. 2012;33(7). doi:10.1016/j.neurobiolaging.2011.02.022

2. Blennow K, Shaw LM, Stomrud E, et al. Predicting clinical decline and conversion to Alzheimer’s disease or dementia using novel Elecsys Aβ(1–42), pTau and tTau CSF immunoassays. *Sci Rep*. 2019;9(1). doi:10.1038/s41598-019-54204-z

3. Hansson O, Seibyl J, Stomrud E, et al. CSF biomarkers of Alzheimer’s disease concord with amyloid-β PET and predict clinical progression: A study of fully automated immunoassays in BioFINDER and ADNI cohorts. *Alzheimer’s and Dementia*. 2018;14(11). doi:10.1016/j.jalz.2018.01.010

4. Mattsson-Carlgren N, Grinberg LT, Boxer A, et al. Cerebrospinal fluid biomarkers in autopsy-confirmed Alzheimer disease and frontotemporal lobar degeneration. *Neurology*. 2022;98(11). doi:10.1212/WNL.0000000000200040

5. Meyer PF, Pichet Binette A, Gonneaud J, Breitner JCS, Villeneuve S. Characterization of Alzheimer Disease Biomarker Discrepancies Using Cerebrospinal Fluid Phosphorylated Tau and AV1451 Positron Emission Tomography. *JAMA Neurol*. 2020;77(4). doi:10.1001/jamaneurol.2019.4749

6. Guo Y, Huang YY, Shen XN, et al. Characterization of Alzheimer’s tau biomarker discordance using plasma, CSF, and PET. *Alzheimers Res Ther*. 2021;13(1). doi:10.1186/s13195-021-00834-3

7. Alzheimer’s Disease Data Initiative. AD Workbench. https://www.alzheimersdata.org/.

8. Ingala S, De Boer C, Masselink LA, et al. Application of the ATN classification scheme in a population without dementia: Findings from the EPAD cohort. *Alzheimer’s and Dementia*. 2021;17(7). doi:10.1002/alz.12292
